# A Novel Prodrug Strategy Based on Reversibly Degradable Guanidine Imides for High Oral Bioavailability and Prolonged Pharmacokinetics of Broad-Spectrum Anti-influenza Agents

**DOI:** 10.1021/acscentsci.4c00548

**Published:** 2024-07-04

**Authors:** Yujeong Jung, Soo Bin Ahn, Taeyang An, Hyeon-Min Cha, Minjae Kim, Hyunjin Cheon, Yejin Jang, Haemi Lee, Byungil Kim, Meehyein Kim, Yan Lee

**Affiliations:** † Department of Chemistry, College of Natural Sciences, Seoul National University, Seoul 08826, Republic of Korea; ‡ Infectious Diseases Therapeutic Research Center, Korea Research Institute of Chemical Technology (KRICT), Daejeon 34114, Republic of Korea; § Graduate School of New Drug Discovery and Development, Chungnam National University, Daejeon 34134, Republic of Korea; ∥ Department of Chemistry and Biochemistry, University of California, San Diego, La Jolla, California 92093, United States; ⊥ School of Transdisciplinary Innovations, Seoul National University, Seoul 08826, Republic of Korea

## Abstract

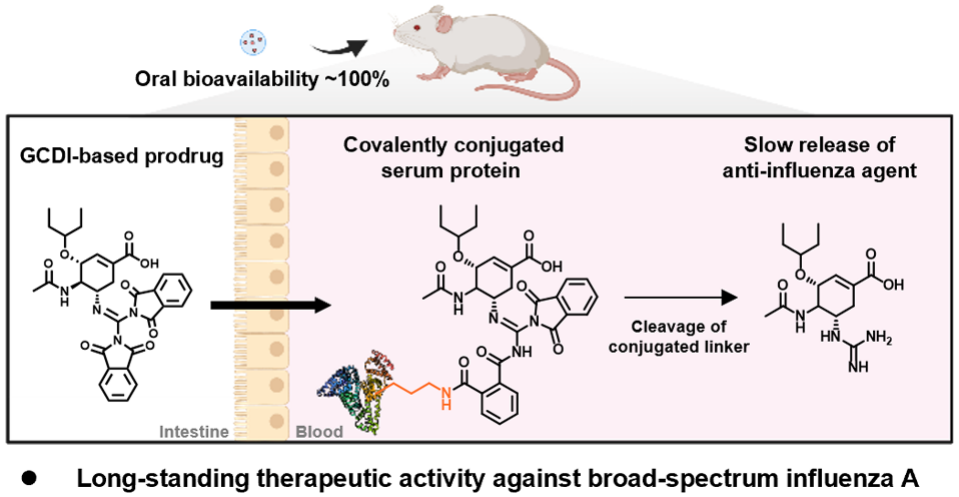

We present orally administrable prodrugs (**OSC-GCDI**s) of guanidino oseltamivir carboxylate (**GOC**) based on guanidine cyclic diimide (GCDI) to treat influenza viruses. By concealing the guanidine group, which significantly limits the intestinal absorption, its prodrugs **OSC-GCDI**s demonstrate dramatic improvement of oral bioavailability. The most promising antiviral substance **OSC-GCDI(P)** readily forms covalent adducts with serum proteins via a degradable linker after the intestinal absorption. Subsequently, the active species, **GOC**, is released from the conjugate in a sustained manner, which greatly contributes to improving pharmacokinetic properties. Because of the remarkable improvements in both oral bioavailability and longevity of its active metabolite, **OSC-GCDI(P)** demonstrates outstanding therapeutic efficacy against both wild-type and oseltamivir-resistant (H275Y) influenza virus strains in a mouse infection model, even with a single oral administration.

## Abstract 2

Guanidine cyclic diimide-based prodrugs dramatically enhance oral bioavailability and pharmacokinetic half-life to show long-time therapeutic activity against broad-spectrum influenza viruses.

## Introduction

**Figure 1:**
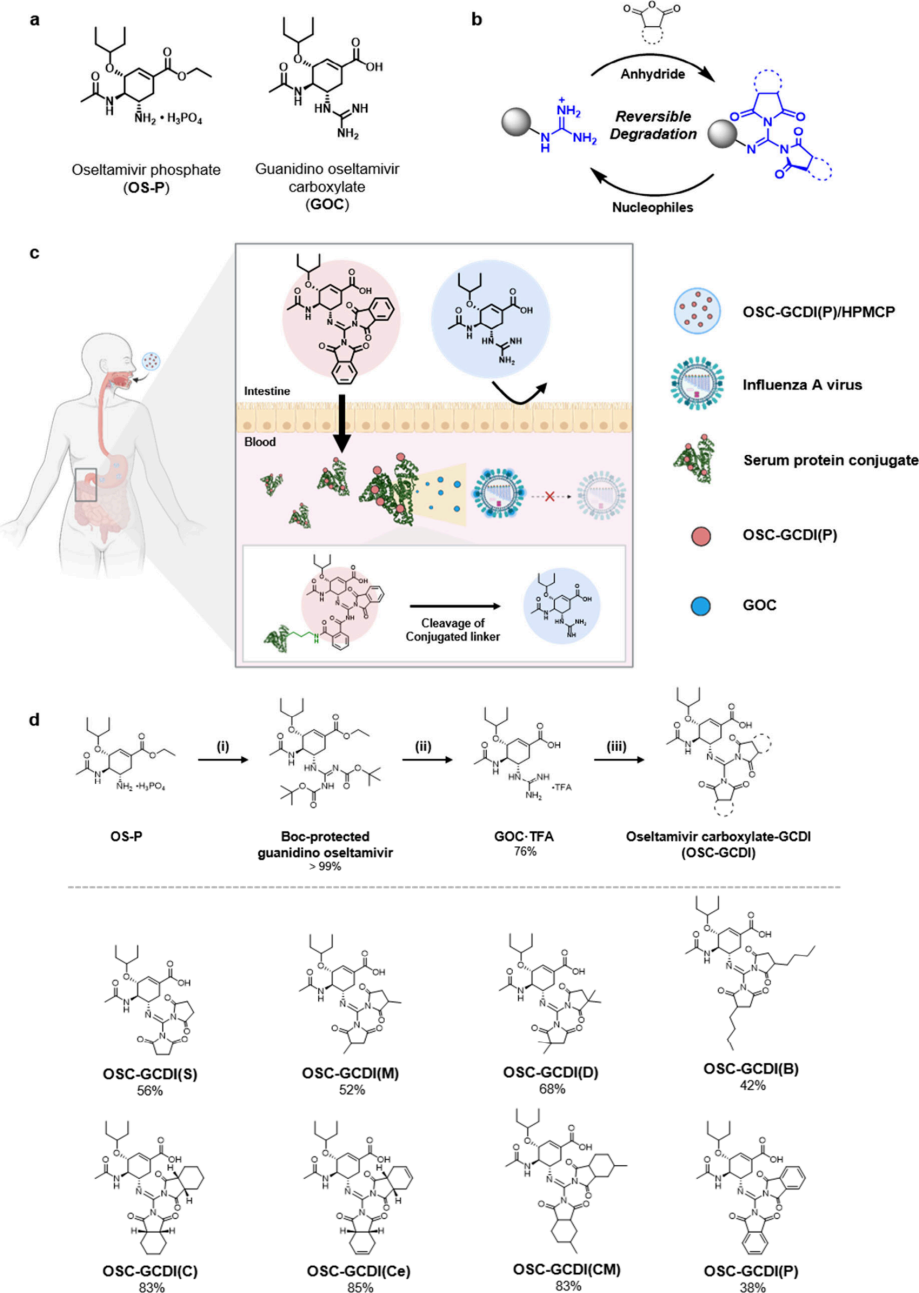
Novel prodrug strategy to enable oral administration of guanidine drugs utilizing the guanidine cyclic diimide (GCDI) structure. (a) Chemical structures of oseltamivir phosphate (**OS-P**) and guanidino oseltamivir carboxylate (**GOC**). (b) Reversible formation and degradation of GCDI. (c) Schematic illustration of the prodrug strategy to enhance oral bioavailability and pharmacokinetic half-lives of the guanidine drugs via installation of the GCDI moieties. (d) Synthesis and chemical structures of **OSC-GCDI**s.

Oseltamivir phosphate (**OS-P**; Tamiflu), an ethyl ester prodrug of oseltamivir carboxylate (**OSC**), is used as a first-line therapy against both influenza A and B viruses, due to its potent antiviral efficacy and high oral bioavailability (>80%) (Figure 1a).^1−5^ However, the regimen for **OS-P** necessitates oral administration twice daily for 5 days, leading to inconvenience for patients. Furthermore, the emergence of **OS**-resistant strains, notably with the H275Y mutation in neuraminidase (NA), presents a great challenge and has become a global concern.^6−13^ These circumstances highlight the urgent need for novel therapeutics effective against wild-type and **OS**-resistant viruses, with reduced dosing frequency.

Among anti-influenza viral drug candidates, guanidino oseltamivir carboxylate (**GOC**) has been identified to be highly potent against wild-type and **OS**-resistant virus strains.^1,5,14−16^ The presence of the guanidine group provides additional interactions with the E226 residue in NA, resulting in a stronger binding affinity of **GOC** with the H275Y mutant NA when compared to **OSC**.^11,16^ However, **GOC** exhibits very low oral bioavailability (<4%), presenting a significant limitation in oral administration.^5,16^ Zanamivir, another guanidine-based antiviral agent, also faces challenges in oral administration and is consequently being developed as an inhalation drug.^5,17^

Bioactive guanidine compounds often encounter oral bioavailability issues and are rarely found in orally administrable drugs. This difficulty mainly arises from the highly polar nature of the charged guanidine group, which impedes penetration through the intestinal barrier.^18−21^ To address these concerns, several approaches have been suggested, including conjugation with ligands,^22−25^ ion pairing,^26−29^ and acylation^30−34^ or *N*-hydroxylation^16,35,36^ of guanidine groups. However, each strategy has its own drawbacks, such as poorly enhanced oral bioavailability, compromised effectiveness, and complex synthetic routes.

Previously, our group has reported a novel chemical structure termed guanidine cyclic diimides (GCDIs) (Figure 1b).^37^ GCDI structures are easily synthesized through reactions between guanidines and cyclic anhydrides under mild conditions. Notably, the GCDI formation effectively conceals the positive charge of the free guanidine group, thereby enhancing lipophilicity. Moreover, GCDIs can undergo reversible degradation into the original guanidine species in the presence of various nucleophiles.

On the basis of these findings, we anticipated that a novel prodrug strategy utilizing the GCDI structure could effectively address the issue of low oral bioavailability of guanidine drugs (Figure 1c). Because of a broad tolerance range of various functional groups under mild GCDI formation conditions, GCDI moieties can be directly installed on guanidine groups without necessitating *de novo* synthetic pathways. Furthermore, by controlling the GCDI structures, the lipophilicity of prodrugs can be delicately tuned for facilitating efficient intestinal absorption. Once absorbed, the GCDI prodrugs can be readily degraded in the bloodstream to release the active species.

In this work, we synthesized eight GCDI-based prodrugs of **GOC**, termed oseltamivir carboxylate guanidine cyclic diimides (**OSC-GCDI**s). Our results demonstrate that their enhanced lipophilicity significantly improved intestinal absorption *in vitro* and *in vivo*. More importantly, the absorbed **OSC-GCDI**s exhibited prolonged pharmacokinetic half-lives in the bloodstream, likely due to noncovalent or covalent interactions with serum proteins. The outstanding enhancement of both oral bioavailability and pharmacokinetics enabled **OSC-GCDI(P)** to exert excellent therapeutic effect against not only wild-type but also H275Y mutant influenza viruses in a mouse infection model, even with a single oral administration. We suggest **OSC-GCDI(P)** as a promising antiviral candidate against broad-spectrum influenza viruses. Moreover, the synthesis of GCDI-based prodrugs can serve as a robust platform to enhance the oral bioavailability and pharmacokinetics of various guanidine-based drugs.

## Results and Discussion

### Synthesis of GCDI Prodrugs

The GCDI structure was introduced onto the guanidine group of **GOC**, an effective compound against the wild-type as well as H275Y mutant influenza viruses.^1,5,14−16^ Through reactions with various cyclic anhydrides (Figure 1d),^37^**OSC-GCDI**s were obtained in reasonable isolated yields (38–85%). To modulate the lipophilicity of the prodrugs, four different 5-membered cyclic anhydrides, succinic anhydride (S), methyl succinic anhydride (M), 2,2-dimethyl succinic anhydride (D), and *n*-butyl succinic anhydride (B), were reacted with **GOC** to form **OSC-GCDI(S)**, **OSC-GCDI(M)**, **OSC-GCDI(D)**, and **OSC-GCDI(B)**, respectively. Moreover, other four cyclic anhydrides with another 6-membered fused ring, *cis*-1,2-cyclohexanedicarboxylic anhydride (C), *cis*-1,2-cyclohexenedicarboxylic anhydride (Ce), 4-methyl-1,2-cyclohexenedicarboxylic anhydride (CM), and phthalic anhydride (P), were reacted with **GOC** to form **OSC-GCDI(C)**, **OSC-GCDI(Ce)**, **OSC-GCDI(CM)**, and **OSC-GCDI(P)**, respectively. All the **OSC-GCDI**s could be directly synthesized from **GOC**, implying that the GCDI-based prodrug strategy can be generally applied to any guanidine-containing drugs at the final stages without requirement of *de novo* synthetic pathways for the prodrugs.

### Lipophilicity of OSC-GCDIs

Lipophilicity is highly correlated to intestinal absorption rate and is regarded as a critical parameter in the development of orally administered drugs.^38^ The octanol–water partition coefficient (log *P*) and distribution coefficient (log *D*) are common standards for assessing drug lipophilicity. Typically, log *P* and log *D* values at pH 7.4 (log *D*_7.4_) for orally administrable drugs range from −1 to 5.^39,40^

**Figure 2:**
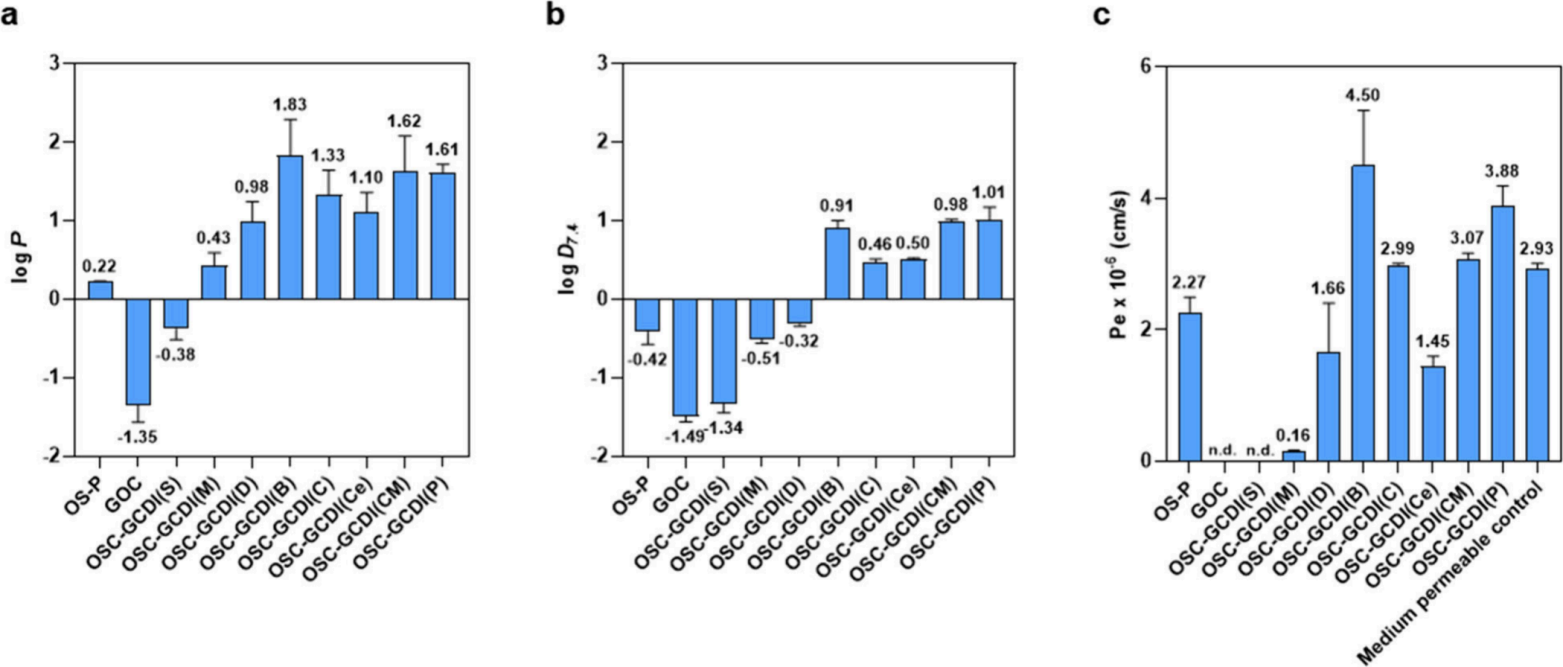
Lipophilicity and membrane permeability of **OSC-GCDI** prodrugs. (a) Octanol–water partition coefficients (log *P*) of **OS-P**, **GOC**, and the **OSC-GCDI** prodrugs. (b) Octanol-phosphate buffer distribution coefficients at pH 7.4 (log *D*_*7.4*_) of **OS-P**, **GOC**, and the **OSC-GCDI** prodrugs. (c) Effective permeability through the artificial membrane of **OS-P**, **GOC**, and the **OSC-GCDI** prodrugs. The data are presented as means ± standard deviations (SD) (*n* = 3). n.d., not detected.

All the **OSC-GCDI**s showed significantly increased log *P* values compared to **GOC**, which exhibited low log *P* values of −1.35 (Figure 2a). In terms of log *D*_7.4_, which reflects the ionizable nature of the free carboxyl group, all the **OSC-GCDI**s, except for **OSC-GCDI(S)**, showed notably increased log *D*_7.4_ values, ranging from −0.51 to 1.01, compared to **GOC** with a log *D*_7.4_ value of −1.49 (Figure 2b). The lipophilicity of **OSC-GCDI**s well correlates with the GCDI structure, increasing with the imide hydrophobicity (S < M < D < B). **OSC-GCDI**s synthesized from fused-ring anhydrides showed log *P* and log *D*_7.4_ values of 1.10–1.62 and 0.46–1.01, respectively. The lipophilicity of **GOC** increased from 8.8-fold in **OSC-GCDI(S)** to 2082-fold in **OSC-GCDI(B)** via the GCDI modification (based on *P*). Except for **OSC-GCDI(S)**, the lipophilicity values of all **OSC-GCDI**s fell within the typical range for orally administrable drugs, illustrating the effectiveness of our GCDI prodrug strategy in enhancing lipophilicity. Notably, all the **OSC-GCDI**s except **OSC-GCDI(S)** and **OSC-GCDI(M)** exhibited even higher log *P* and log *D*_7.4_ values than **OS-P**, which has excellent oral bioavailability.

### Parallel Artificial Membrane Permeability Assay (PAMPA) of OSC-GCDIs

While the precise absorption mechanism of the GCDI prodrugs remained unclear at that stage, it was plausible that these prodrugs would exhibit a higher passive diffusion rate through the duodenal mucosa compared to **GOC**. To assess this hypothesis, we conducted the parallel artificial membrane permeability assay (PAMPA) for simulating passive transcellular permeation.^41^

The permeation rate of each **OSC-GCDI** through the artificial membrane was calculated by measuring the effective permeability (*P*_e_). The permeability of **GOC** was almost negligible under these conditions; however, all **OSC-GCDI**s except **OSC-GCDI(S)** exhibited a remarkable increase in *P*_e_ (Figure 2c). In line with the lipophilicity tendency, **OSC-GCDI**s with bulkier imide groups tended to have higher *P*_e_ values. It is noteworthy that four **OSC-GCDI**s, specifically **OSC-GCDI(B)**, **OSC-GCDI(C)**, **OSC-GCDI(CM)**, and **OSC-GCDI(P)**, demonstrated *P*_e_ values that were comparable to or even exceeding those of **OS-P** (*P*_e_ = 2.27 × 10^–6^ cm/s) and a control compound with medium permeability (*P*_e_ = 2.93 × 10^–6^ cm/s). These findings led us to expect that GCDI derivatization could significantly enhance the intestinal absorption of guanidine drugs, potentially enabling their successful oral administration.

### Activation of OSC-GCDIs and Conjugation to Albumin

Our group has previously demonstrated that the GCDI structures are readily degraded, releasing their parent guanidine molecules in the presence of various nucleophiles.^37^ Consequently, we anticipated that GCDI prodrugs would regenerate the active drug, **GOC**, after intestinal absorption. Initially, we evaluated the stability or degradability of **OSC-GCDI**s (Table 1 and Figure S1) in phosphate buffers with different pHs. In general, **OSC-GCDI**s featuring bulkier imide groups exhibited greater stability against hydrolysis, likely due to reduced water accessibility to the imide carbonyl groups. Moreover, the hydrolysis rate of the GCDI prodrugs escalated with increasing pH. At pH 2.0 (mimicking stomach conditions), all **OSC-GCDI**s, except **OSC-GCDI(P)** with a half-life of 94 min, maintained excellent stability with half-lives longer than 24 h. At pH 6.5 (representing duodenal conditions), most prodrugs presented half-lives beyond 5 h, except for **OSC-GCDI(P)** with a half-life of 88 min. The least stable **OSC-GCDI(P)** exhibited half-life of 14.1 min at pH 7.4, whereas the most stable **OSC-GCDI(D)** demonstrated a half-life exceeding 8 h at pH 7.4. Other prodrugs showed half-lives ranging from 1 to 8 h at pH 7.4, and from 3 min to 3 h at pH 8.0. As suggested in previous studies,^42^ we suspect that the exceptional hydrolysis rate of **OSC-GCDI(P)** can be attributed to additional ring strain of the phthalimide structure to maximize the electron delocalization, which may lead to intrinsic instability of **OSC-GCDI(P)**. Additionally, given that aromatic carboxylic acids generally have higher acidity than aliphatic acids, the negative charge of the amic acid intermediate, which is generated by the degradation of **OSC-GCDI(P)**, is expected to be stabilized more efficiently in the aromatic ring compared to those from other **OSC-GCDI**s with the aliphatic imide structures. These characteristics may contribute to the faster hydrolysis of **OSC-GCDI(P)**.

**Table 1:** Half-Lives of the **OSC-GCDI** Prodrugs in Phosphate Buffer and Serum

	*T*_1/2_ (min)^a^					
	Phosphate buffer				Serum	
Compound	pH 2.0^b^	pH 6.5^b^	pH 7.4^b^	pH 8.0^b^	BALB/c^c^	Human^c^
OSC-GCDI(S)	>1440	770.2	101.9	42.0	17.5	16.7
OSC-GCDI(M)	>1440	866.4	108.3	39.5	13.5	18.4
OSC-GCDI(D)	>1440	>1440	495.1	161.2	158	117
OSC-GCDI(B)	>1440	>1440	462.1	157.5	17.1	52.5
OSC-GCDI(C)	>1440	630.1	81.6	26.8	23.8	26.2
OSC-GCDI(Ce)	>1440	1155	105.0	54.2	35.7	53.3
OSC-GCDI(CM)	>1440	693.1	99.0	3.92	13.3	31.2
OSC-GCDI(P)	93.7	87.7	14.1	<1	1.3	1.3

a Determined by HPLC analysis of the remaining prodrugs at various time points. The calculation of the half-lives was based on the assumption of pseudo-first-order kinetics of the degradation.

b Examined in phosphate-buffered saline (50 mM phosphate, 154 mM ionic strength, 37 °C).

c Examined in serum at a prodrug concentration of 200 μM at 37 °C.

To ascertain if **OSC-GCDI**s convert into their active form, **GOC**, after absorption into the bloodstream, we incubated the **OSC-GCDI**s in BALB/c mouse and human serum and then quantified both unhydrolyzed **OSC-GCDI**s and hydrolyzed **GOC** (Table 1 and Figure S2). **OSC-GCDI**s underwent significantly faster degradation in serum than in phosphate buffers. Most **OSC-GCDI**s showed half-lives of approximately 10–30 min, with bulkier imide groups generally associated with longer half-lives. **OSC-GCDI(P)** displayed the shortest half-life (1.3 min), while **OSC-GCDI(D)** had the longest (>100 min) in serum. The degradation rate of **OSC-GCDI**s was not accelerated by Sprague–Dawley rat liver microsomes (Table S1). The results supported that the degradation of **OSC-GCDI**s or activation to **GOC** might be based primarily on reactions in serum, not by hepatic metabolism.

The liberation of **GOC** was notably slower than the degradation of GCDI species in serum (Figure S2). Particularly, **OSC-GCDI(P)**, which exhibited only a half-life of 1.3 min in serum, released 37% of **GOC** after 30 min and 68% after 24 h. Similarly, **OSC-GCDI(M)** and **OSC-GCDI(C)** released **GOC** in a sustained and gradual manner after the original compounds have completely disappeared, suggesting the existence of intermediates prior to the full hydrolysis into **GOC**. Given their structural features, the conversion of **OSC-GCDI**s to **GOC** theoretically requires four nucleophilic attacks on the imide rings, possibly yielding various guanidine amic acids as likely intermediates (Figure S3).

**Figure 3:**
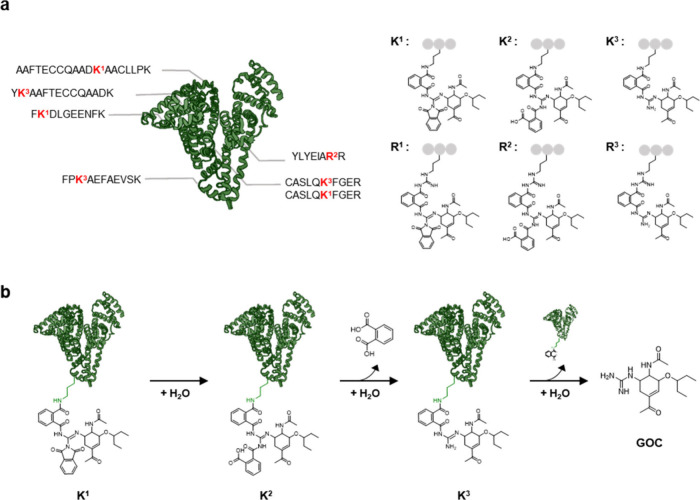
Covalent conjugation of **OSC-GCDI**s to human serum albumin (HSA). (a) Amino acid residues conjugated with **OSC-GCDI** in HSA, which were identified by MALDI-TOF MS. Suggested structures of the covalent linkage between the sequence and **OSC-GCDI(P)** are shown. (b) A proposed mechanism of the **GOC** release from HSA. The lysine adduct of **OSC-GCDI(P)**, K^1^, can be successively hydrolyzed to K^2^, K^3^, and finally, **GOC**.

The more rapid degradation of **OSC-GCDI**s in serum compared to buffers implies the possibility of nucleophilic attacks on the GCDI structure by nonwater nucleophiles. We suspected that the nucleophilic residues of serum proteins could attack the GCDI imide rings. The increased lipophilicity of **OSC-GCDI**s might facilitate their noncovalent association with albumin, thereby increasing the probability of nucleophilic attacks on the GCDI structures. Corroborating this hypothesis, MALDI-TOF MS analysis of the mixture of **OSC-GCDI(P)** and human serum albumin (HSA) provided the evidence of covalent adducts between them (Figures 3a and S4). Several lysine and arginine residues in HSA were covalently conjugated to the prodrug via amide or acyl guanidine linkages.

The covalent conjugation of the GCDI prodrugs onto serum proteins can provide a new strategy to increase pharmacokinetic half-lives. Similar to other albumin-conjugated drugs,^43−46^ the GCDI prodrugs are likely to remain in the bloodstream in their conjugated form with albumin, thereby reducing their hepatic intracellular metabolism and excretion rates. Considering the vulnerability of the guanidine amide linkage between **GOC** and albumin to nucleophilic attacks, it was postulated that **GOC** could be gradually released from albumin via hydrolytic intermediates (Figure 3b). Indeed, incubation of **OSC-GCDI(P)**-HSA and **OSC-GCDI(C)**-HSA conjugates resulted in the release of **GOC** in a sustained manner for at least 72 h (Figure S5).

### *In Vitro* Antiviral Activity of OSC-GCDIs

To evaluate the antiviral effectiveness of **OSC-GCDI**s and **GOC**, we conducted a cell-based assay to assess the cytopathic effects resulting from the infection of different influenza virus (sub)types, such as A/Puerto Rico/8/1934 (PR8; H1N1), A/Hong Kong/8/1968 (HK; H3N2), and B/Lee/1940 (Lee) (Table 2). **OSC-GCDI**s exhibited comparable activity against PR8, with EC_50_ values ranging from 0.01 to 0.09 μM. For HK-infected cells, the EC_50_ values was below 0.04 μM, with **OSC-GCDI(C)** showing the most potent inhibition (EC_50_, < 0.005 μM). These compounds demonstrated variable antiviral efficacy against the influenza B virus strain, Lee, with EC_50_ values ranging from 2.21 to 72.38 μM, and the lowest values observed for **OSC-GCDI(C)** and **OSC-GCDI(P)**. Collectively, among the **OSC-GCDI**s tested, **OSC-GCDI(C)** and **OSC-GCDI(P)** demonstrated relatively more potent antiviral activity against influenza A and B viruses, *in vitro*.

**Table 2:** Antiviral Activity of **OSC-GCDI** Prodrugs against Influenza A and B Viruses in MDCK Cells

		EC_50_ (μM)^b^(S.I.)^c^						
Compound	CC_50_ (μM)^a^	PR8^d^	HK^e^	Lee^f^	rgPR8^g^	rgPR8 (H275Y)^h^	KR2785 (H275Y)^i^	rgKR09 (H275Y)^j^
OSC-GCDI(S)	>100	0.06 ± 0.01	0.03 ± 0.01	11.10 ± 1.20	0.07 ± 0.01	0.99 ± 0.01	8.55 ± 2.15	2.20 ± 0.10
		(>1667)	(>3333)	(>9.01)	(>1429)	(>101)	(>11.7)	(>45.5)
OSC-GCDI(M)	>100	0.04 ± 0.01	0.02 ± 0.01	5.55 ± 1.65	0.02 ± 0.01	0.74 ± 0.05	4.61 ± 0.35	1.96 ± 0.15
		(>2500)	(>5000)	(>18.0)	(>5000)	(>135.1)	(>21.7)	(>51.0)
OSC-GCDI(D)	>100	0.07 ± 0.01	0.03 ± 0.01	13.70 ± 2.85	0.05 ± 0.01	1.52 ± 0.20	9.25 ± 2.40	4.37 ± 1.10
		(>1429)	(>3333)	(>7.3)	(>2000)	(>65.8)	(>10.8)	(>22.9)
OSC-GCDI(B)	>100	0.09 ± 0.01	0.04 ± 0.01	72.38 ± 1.35	0.08 ± 0.01	3.41 ± 0.01	17.59 ± 2.90	12.86 ± 0.65
		(>1111)	(>2500)	(>1.4)	(>1250)	(>29.3)	(>5.7)	(>7.8)
OSC-GCDI(C)	>100	0.01 ± 0.01	<0.005	2.21 ± 0.20	0.01 ± 0.01	0.49 ± 0.01	5.91 ± 0.85	3.70 ± 0.50
		(>10,000)	(>20,000)	(>45.2)	(>10,000)	(>204.1)	(>16.9)	(>27.0)
OSC-GCDI(Ce)	>100	0.03 ± 0.01	0.02 ± 0.01	11.96 ± 4.85	0.01 ± 0.01	1.90 ± 0.10	13.22 ± 0.10	5.21 ± 0.02
		(>3333)	(>5000)	(>8.4)	(>10,000)	(>52.6)	(>7.6)	(>19.2)
OSC-GCDI(CM)	>100	0.03 ± 0.01	0.01 ± 0.01	8.36 ± 1.00	0.01 ± 0.01	1.20 ± 0.05	13.98 ± 5.15	4.43 ± 0.25
		(>3333)	(>10,000)	(>12.0)	(>10,000)	(>83.3)	(>7.2)	(>22.7)
OSC-GCDI(P)	>100	0.03 ± 0.01	0.01 ± 0.01	2.26 ± 0.40	0.01 ± 0.01	0.48 ± 0.01	4.61 ± 0.35	4.61 ± 0.05
		(>3333)	(>10,000)	(>44.2)	(>16,667)	(>208.3)	(>21.7)	(>21.7)
GOC	>100	0.005 ± 0.001	<0.005	0.70 ± 0.15	0.005 ± 0.001	0.09 ± 0.01	0.78 ± 0.01	0.11 ± 0.01
		(>20,000)	(>20,000)	(>142.9)	(20,000)	(>1111)	(>128.2)	(>909.1)
OSC	>100	0.10 ± 0.01	<0.005	1.29 ± 0.25	0.05 ± 0.01	13.71 ± 2.70	56.61 ± 0.30	25.87 ± 0.65
		(>1000)	(>20,000)	(>77.5)	(>2000)	(>7.3)	(>1.8)	(>3.9)
RBV^k^	>100	11.1 ± 0.01	21.78 ± 1.10	13.41 ± 0.95	15.95 ± 1.55	16.43 ± 0.45	18.26 ± 1.30	15.79 ± 0.15
		(>9.0)	(>4.6)	(>7.5)	(>6.3)	(>6.1)	(>6.6)	(>6.3)
AMT^l^	>100	>100	1.56 ± 0.40	>100	>100	>100	>100	>100
		(n.d.)^m^	(>64.1)	(n.d.)	(n.d.)	(n.d.)	(n.d.)	(n.d.)

a Fifty percentage cytotoxic concentration to MDCK cells.

b Fifty percent effective concentration against influenza virus.

c Selectivity index, the ratio of CC_50_ to EC_50_.

d A/Puerto Rico/8/1934 (H1N1).

e A/Hong Kong/8/1968 (H3N2).

f B/Lee/1940.

g Reverse genetically generated A/Puerto Rico/8/1934.

h Reverse genetically generated A/Puerto Rico/8/1934 with the H275Y mutation in NA.

i A/Korea/2785/2009 (H1N1) with the H275Y mutation in NA.

j Reverse genetically generated A/Korea/09/2009Δ53–60 with the H275Y mutation and amino acid deletion between 53 and 60 in NA.

k Ribavirin.

l Amantadine.

m Not determined. The data are presented as means ± standard deviations (SD) (*n* = 3).

To evaluate broad-spectrum antiviral efficacy, particularly against **OS**-resistant viruses with the H275Y mutation in NA, we further evaluated the change in antiviral effectiveness against the reverse genetically rescued PR8 virus (rgPR8) and its corresponding H275Y mutant strain (rgPR8(H275Y)) (Table 2). The calculated resistance factors (RFs), defined as the ratio of EC_50_ values against rgPR8(H275Y) to wild-type rgPR8, indicated a substantial reduction in antiviral resistance for all **OSC-GCDI**s (RFs, 14.1–190) and **GOC** (RF, 18.0), both of which were discriminated from that of **OSC** (RF, 274). Additionally, we investigated the antiviral activity of these **OSC-GCDI**s against other H275Y mutants, A/Korea/2785/2009 (KR2785) and rgA/Korea/09/2009Δ53–60 (rgKR09). The **OSC-GCDI**s surpassed **OSC** in antiviral activity against the H275Y mutant viruses, although they were not as potent as **GOC**. Given that full activation from **OSC-GCDI**s to **GOC** take a substantial amount of time in buffers or serum (Table 1 and Figures S1–S3), the observed reduced *in vitro* efficacy of the GCDI prodrugs is understandable. Among the **OSC-GCDI**s, **OSC-GCDI(S)**, **OSC-GCDI(M)**, **OSC-GCDI(C)**, and **OSC-GCDI(P)** exhibited superior activity relative to the remaining four compounds. Taken together, GCDI formation from **OSC** appeared to contribute to recovering its reduced antiviral activity against the H275Y mutant viruses.

### *In Vivo* Pharmacokinetics and Antiviral Activity of OSC-GCDIs

**GOC** is more potent than **OSC** both against wild-type and **OS**-resistant influenza viruses (Table 2).^16,47^ However, the limited oral bioavailability of **GOC** has hampered its effectiveness for clinical development. Encouraged by the improved *in vitro* permeability of **OSC-GCDI**s and their successful conversion to active **GOC** in serum, we explored the *in vivo* pharmacokinetic (PK) properties and antiviral activity of **OSC-GCDI(C)** and **OSC-GCDI(P)**, which exhibited the most potent and broad-spectrum antiviral activity *in vitro* (Table 2).

In preliminary experiments, **GOC** as a trifluoroacetate salt (**GOC·TFA**), **OSC-GCDI(C)**, and **OSC-GCDI(P)** were orally administered to mice. Pharmacokinetic analysis over 24 h revealed that **GOC·TFA** had an oral bioavailability (*F*_t_) of 9.69% and a plasma half-life (*T*_1/2_) of 4 h (Figure S6a). However, the *F*_t_ values for **OSC-GCDI(C)** and **OSC-GCDI(P)** were 0.48% and 5.95%, respectively (Figure S7), less than that of **GOC·TFA**. Notably, we found that **OSC-GCDI(P)** exhibits a remarkably longer *T*_1/2_ (6.93 h) compared to **GOC·TFA**, which has a *T*_1/2_ of 4.00 h, and **OSC-GCDI(C)**, for which *T*_1/2_ could not be estimated. These results suggest that although orally administered **OSC-GCDI(P)** has limited bioavailability, it facilitates sustained release of the active metabolite, **GOC**, in mouse blood.

On the basis of the extended half-life of **OSC-GCDI(P)**, we hypothesized that consistent concentrations of **GOC** metabolized from orally administered **OSC-GCDI(P)** could effectively protect mice from influenza virus infection. Nevertheless, the therapeutic efficacy of **OSC-GCDI(P)** in a mouse infection model was inferior to those of **OS-P** and **GOC**, as measured by dose response in survival rates and mortality (Figure S8), likely attributed to suboptimal *C*_max_ and resulting insufficient oral bioavailability. We assumed that these relatively poor antiviral activity of **OSC-GCDI(P)** in oral administration especially at lower doses could result from its vulnerability against nucleophiles prior to intestinal adsorption. Although **OSC-GCDI(P)** is stable in acidic buffers simulating stomach pH, it is prone to hydrolysis into **GOC** under neutral or basic conditions (Table 1), and to covalent binding with proteins by their nucleophilic attack to **OSC-GCDI(P)** along the gastrointestinal track (Figure 3). Such reactions may result in the accumulation of metabolites or metabolic intermediates from **OSC-GCDI(P)** with reduced intestinal absorption, underscoring the need of formulation for protecting it from premature degradation.

### *In Vivo* Pharmacokinetics and Antiviral Activity of OSC-GCDIs Formulated with HPMCP

To minimize the gastrointestinal degradation before intestinal absorption, **OSC-GCDI**s were formulated with a gastro-retentive drug delivery vehicle, hydroxypropyl methylcellulose phthalate (HPMCP), designated as **OSC-GCDI**/HPMCP. **OSC-GCDI(C)** and **OSC-GCDI(P)** were successfully encapsulated within HPMCP through coprecipitation in an acidic solution (Figure S9). The HPMCP encapsulation itself has no significant effect on the passive diffusion into membranes as shown in the PAMPA results of **GOC**/HPMCP and **OSC-GCDI(P)**/HPMCP (Table S2).

**Figure 4:**
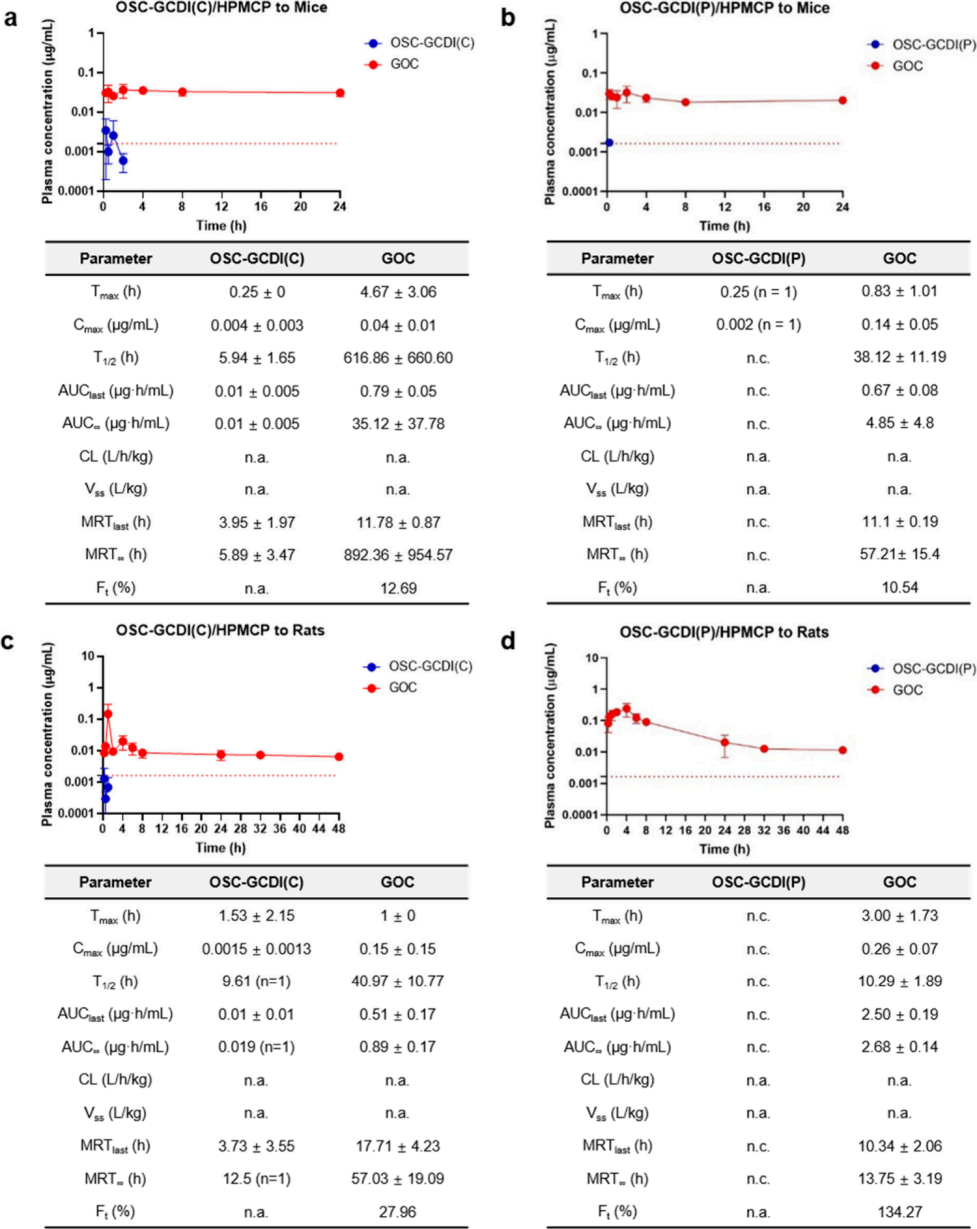
Pharmacokinetic properties of **OSC-GCDI**s/HPMCP in mice and rats. (a, b) Plasma concentrations and pharmacokinetic parameters of **OSC-GCDI**s (blue ●) and **GOC** (red ●) at various time points after oral administration of (a) **OSC-GCDI(C)**/HPMCP and (b) **OSC-GCDI(P)**/HPMCP to mice at doses of 5 mg/kg based on the amount of **OSC-GCDI**s within the complex. (c, d) Plasma concentrations and pharmacokinetic parameters of **OSC-GCDI**s (blue ●) and **GOC** (red ●) at various time points after oral administration of (c) **OSC-GCDI(C)**/HPMCP and (d) **OSC-GCDI(P)**/HPMCP to rats at doses of 10 mg/kg based on the amount of **OSC-GCDI**s within the complex. The data are presented as means ± standard deviations (SD) from three mice or rats, except for those from **OSC-GCDI(C)** in panel (a) at 0.25 h and **OSC-GCDI(P)** in panel (b) at 0.25 h, which were detected in one mouse among the three. The **GOC** concentration in plasma equivalent to an EC_50_ value (0.005 μM) against PR8 (A/H1N1) in MDCK cells is indicated by red dashed lines. n.a., not applicable; n.c., not calculated.

In an initial mouse pharmacokinetics analysis for 24 h, the *F*_t_ values of **OSC-GCDI(C)**/HPMCP and **OSC-GCDI(P)**/HPMCP were determined to be 12.69% and 10.54%, respectively, which were notably higher than those of unformulated **OSC-GCDI(C)** and **OSC-GCDI(P)** (Figure 4a and b, and Figure S7a and b). More importantly, we found that plasma **GOC** concentrations were almost constant even 24 h postadministration, implying the possibility of prolonged **GOC** release in the bloodstream. Consequently, we extended our pharmacokinetic analysis to rats for a longer period of 48 h, observing that **OSC-GCDI(C)**/HPMCP and **OSC-GCDI(P)**/HPMCP exhibited remarkably higher *F*_t_ values of 27.96% and 134.27%, respectively (Figure 4c and d), compared to **GOC·TFA** with an *F*_t_ value of 2.36% (Figure S6b). The *F*_t_ exceeding 100% for **OSC-GCDI(P)**/HPMCP may be attributed to the slower clearance rate of **OSC-GCDI(P)** as a result of covalent interactions with plasma proteins.^48,49^ These findings proved enhanced intestinal adsorption of **OSC-GCDI**s when encapsulated with HPMCP. In addition, given that the *P*_e_ value of **OSC-GCDI(C)** is comparable to that of **OSC-GCDI(P)** (Figure 2c), we suspect that the lower *F*_t_ value of **OSC-GCDI(C)** compared to that of **OSC-GCDI(P)** probably did not result from poor intestinal absorption of **OSC-GCDI(C)**. Considering that *F*_t_ is defined as the molar ratio of detected active species (**GOC**) to orally administered prodrug (**OSC-GCDI**), not only intestinal absorption but also activation rate *in vivo* is a crucial factor. Therefore, the lower *F*_t_ of **OSC-GCDI(C)** may be due to slower activation of **OSC-GCDI(C)** into **GOC** in the bloodstream (Figures S2 and S5), which may lead to incomplete activation within 48 h in the rat model. Moreover, **OSC-GCDI(C)**/HPMCP and **OSC-GCDI(P)**/HPMCP exhibited *T*_1/2_ of 40.97 and 10.29 h, respectively, which were significantly longer than that of **GOC·TFA** (3.57 h). The four times longer *T*_1/2_ of **OSC-GCDI(C)** than that of **OSC-GCDI(P)** also supports our argument for the slower activation of **OSC-GCDI(C)** than **OSC-GCDI(P)**. Furthermore, **OSC-GCDI**/HPMCP formulations maintained the **GOC** concentrations above the EC_50_ value (5.0 nM equal to 1.63 ng/mL) for at least 2 days. The higher lipophilicity of **OSC-GCDI**s could facilitate noncovalent binding to albumin, thereby prolonging circulation.^50−52^ Taken together, the result suggested that **OSC-GCDI**s circulates as a covalently conjugated form with serum proteins in the bloodstream, releasing **GOC** in a sustained manner to increase *T*_1/2_ (Figure 3).

**Figure 5:**
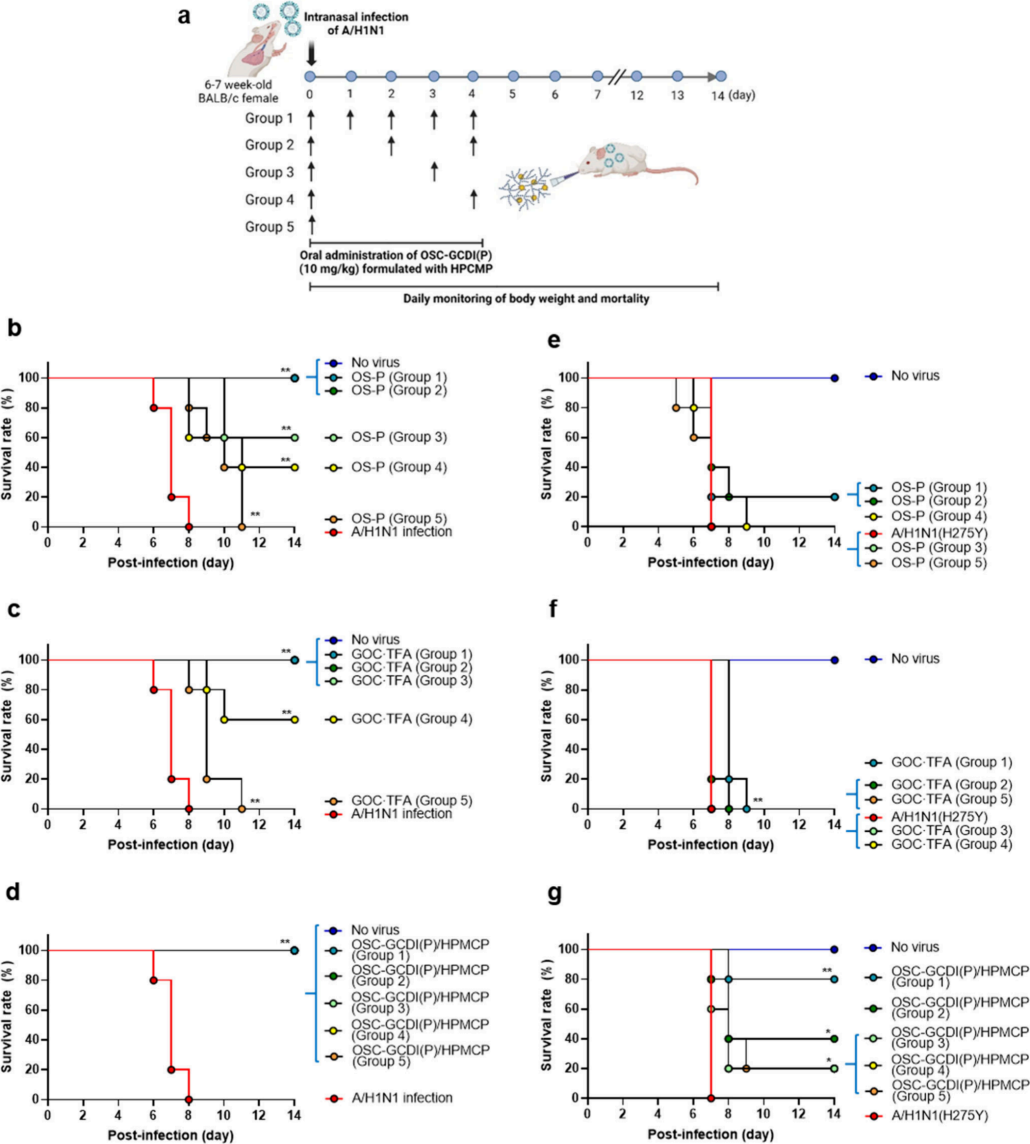
Effect of oral administration frequency on therapeutic efficacy of **OSC-GCDI(P)**/HPMCP in mice infected with a wild-type influenza virus and an **OS**-resistant influenza virus harboring the H275Y mutation in NA. (a) Schematic representation illustrating the administration frequency of **OSC-GCDI(P)** formulated with HPMCP to mice infected by wild-type mouse-adapted virus (maPR8) or the **OS**-resistant A/H1N1 virus (rgA/Korea/09/2009Δ53–60(H275Y), referred to as A/H1N1(H275Y)). Each compound was additionally administered at different intervals: days 1 to 4 postinfection in group 1, days 2 and 4 in group 2, day 3 in group 3, and day 4 in group 4. In Group 5, there was no additional treatment after the single administration. (b–d) Survival rates of maPR8-infected mice after (b) **OS-P**, (c) **GOC·TFA**, or (d) **OSC-GCDI(P)** administration. (e–g) Survival rates of A/H1N1(H275Y)-infected mice after (e) **OS-P**, (f) **GOC·TFA**, or (g) **OSC-GCDI(P)** administration. Groups with overlapping survival rate curve are indicated with blue brackets. Statistical significance was determined by comparing time-course survival rates to the maPR8- or A/H1N1(H275Y)-infected group. *, *P* < 0.05; **, *P* < 0.01.

The pharmacokinetic analysis results motivated an exploration into whether **OSC-GCDI(P)**/HPMCP could extend the dosing interval of the antiviral agent (Figure 5). We orally administrated an identical dose, 10 mg/kg, of **OSC-GCDI(P)**/HPMCP, as well as **OS-P** or **GOC·TFA**, to mice before maPR8 infection at an MLD_50_ of 10. Subsequently, they were treated every day (group 1) or every other day (group 2) for 5 days. To impose more stringent conditions, additional treatments were confined on day 3 (group 3) or day 4 (group 4) or discontinued (group 5) after the first administration (Figure 5a).

When administered with **OS-P**, there were minimal body weight changes in group 1 with complete survival (Figures 5b and S10a). In groups 2, 3, and 4, body weights initially decreased but have recovered by day 9, resulting in survival rates of 100, 60, and 40%, respectively. However, all mice in group 5 were dead by day 10 after infection. The results well aligned with the short half-life of **OS-P** less than 1 day.^53^**GOC·TFA** showed antiviral responses comparable or slightly better than **OS-P**, resulting in complete survival in groups 1, 2, and 3, but 60 and 0% in groups 4 and 5, respectively (Figures 5c and S10b). Notably, when **OSC-GCDI(P)**/HPMCP was administered, mice survived in all groups (Figures 5d and S10c). Although body weight decreased transiently at the initial stage in group 5, it returned to nearly normal by day 14 postinfection. The data strongly suggested that consistent plasma concentration and extended retention time of **GOC** metabolized from **OSC-GCDI(P)** are further enhanced when formulated with HPMCP, making it orally available even after a single shot to treat influenza viral infection.

When **OSC-GCDI(P)**/HPMCP was administrated twice-a-day (b.i.d.) for over 5 days, similar to the standard dosing of **OS-P** in the clinic, **OSC-GCDI(P)**/HPMCP did not show the antiviral potency over those of **GOC·TFA** and **OS-P** (Figures S8 and S11). The results also supported that much stronger therapeutic effect of **OSC-GCDI(P)**/HPMCP than **GOC·TFA** and **OS-P** with a single oral administration was originated from the maintenance of the **GOC** concentration above the EC_50_ value for a prolonged period.

### *In Vivo* Antiviral Activity of OSC-GCDI(P)/HPCMP against OS-Resistant Virus

We further explored whether **OSC-GCDI(P)**/HPMCP is effective against an **OS**-resistant influenza virus carrying an H275Y mutation, rgKR09, in a mouse model. Following the regimen illustrated in Figure 5a, mice infected with an MLD_50_ of 5 of the virus were orally administered with **OS-P**, **GOC·TFA**, and **OSC-GCDI(P)**/HPMCP.

When administered with **OS-P**, both daily treatment (group 1) and every-other-day treatment (group 2) for 5 days exhibited drastic body weight loss and a survival rate of 20%, which was not statistically significant compared to the virus-only group (A/H1N1(H275Y)) (Figures 5e and S12a). In other groups (groups 3–5), it failed to improve body weight changes and survival rates. In another control set with **GOC·TFA**, all mice were eventually dead, though statistical significance was found in group 1 with a delayed mean survival date (Figures 5f and S12b).

In contrast, when the mice were treated with **OSC-GCDI(P)**/HPMCP, the alleviation of body weight decrease was particularly evident in group 1 (Figures 5g and S12c). As a result, survival rates improved to 80% in group 1, 40% in group 2, and 20% in groups 3 to 5, demonstrating statistical significance. Although the complete therapeutic efficacy of **OSC-GCDI(P)**/HPMCP with reduced treatment intervals was not achieved, presumably due to the use of a highly lethal virus dose, therapeutic effectiveness of **OSC-GCDI(P)**/HPMCP against the **OS**-resistant virus surpassed that of **OS-P** and **GOC·TFA**. Taken together, the results suggest that oral administration of **OSC-GCDI(P)**/HPMCP not only offers protection against a wild-type influenza virus but also against an **OS**-resistant variant with NA(H275Y).

## Conclusion

In the present study, we have developed novel GCDI-based prodrugs of **GOC** with high oral bioavailability and extended pharmacokinetics, targeting both wild-type and **OS**-resistant influenza virus strains. The charge of the guanidine group is effectively and temporarily concealed by the GCDI group, increasing the lipophilicity and facilitating the drug absorption through the intestinal barrier. This is the first study of GCDI-based prodrugs with moderate reactivity with nucleophiles, allowing them to be covalently conjugated to serum proteins via a biodegradable linker. **GOC**, the active compound, can be regenerated in serum in a sustained manner through the hydrolysis of the linker.

Leveraging synergetic effects on intestinal adsorption and slow metabolization, **OSC-GCDI(P)** has significantly enhanced bioavailability and pharmacokinetic half-life when administered orally. Furthermore, **OSC-GCDI(P)** showed markedly stronger therapeutic effects compared to **OS-P** and **GOC** against both wild-type and H275Y mutant influenza virus infections with just a single oral dose. **OSC-GCDI**s hold potential as a promising new option against emerging or re-emerging drug-resistant influenza viruses. Additionally, the GCDI-based prodrug design strategy represents a versatile platform technology to enhance the oral bioavailability and serum longevity of various drug candidates containing guanidine groups.

## Supporting Information Available

The Supporting Information is available free of charge on the ACS Publications Web site. . The Supporting Information is available free of charge at https://pubs.acs.org/doi/10.1021/acscentsci.4c00548.

Stability and activation profiles of **OSC-GCDI**s, proposed activation mechanism of **OSC-GCDI**s, analysis data of the **OSC-GCDI**-HSA conjugates, pharmacokinetic properties, therapeutic efficacy, HPMCP particle characterization, detailed experimental procedures, synthetic procedures, NMR spectra of compounds, and supplemental references (PDF)
Transparent Peer Review report available (PDF)
