# Supplementary material for: A Novel Prodrug Strategy Based on Reversibly Degradable Guanidine Imides for High Oral Bioavailability and Prolonged Pharmacokinetics of Broad-Spectrum Anti-influenza Agents
Source: ACS Cent Sci. 2024 Jul 4;10(8):1573–84. doi: 10.1021/acscentsci.4c00548 (PMC11363325; doi:10.1021/acscentsci.4c00548)
Supplement: Supplementary file 1 — oc4c00548_si_001.pdf [file oc4c00548_si_001.pdf]

Supplemental Information

A Novel Prodrug Strategy Based on Reversibly Degradable Guanidine Imides for High Oral Bioavailability and Prolonged Pharmacokinetics of Broad-Spectrum Anti-Influenza Agents

Yujeong Jung[1,#], Soo Bin Ahn[2,3,#], Taeyang An[4,#,*], Hyeon-Min Cha[2,3], Minjae Kim[1], Hyunjin Cheon[1], Yejin Jang[2], Haemi Lee[2], Byungil Kim[2], Meehyein Kim[2,3,*], and Yan Lee[1,5,*]

[1]Department of Chemistry, College of Natural Sciences, Seoul National University, Seoul 08826, Republic of Korea

[2]Infectious Diseases Therapeutic Research Center, Korea Research Institute of Chemical Technology (KRICT), Daejeon 34114, Republic of Korea

[3]Graduate School of New Drug Discovery and Development, Chungnam National University, Daejeon 34134, Republic of Korea

[4]Department of Chemistry and Biochemistry, University of California, San Diego, La Jolla, California 92093, United States

[5]School of Transdisciplinary Innovations, Seoul National University, Seoul 08826, Republic of Korea

#These authors contributed equally to this work.

*Email: taan@ucsd.edu (Taeyang An), mkim@krict.re.kr (Meehyein Kim), gacn@snu.ac.kr (Yan Lee)

Contents

Supplemental Data

Fig. S1. Stability of OSC-GCDIs in buffers S3

Fig. S2. Stability of OSC-GCDIs in serum S4

Table S1. *In* vitro microsomal stability of GOC, OSC-GCDI(D) and OSC-GCDI(P)..........S5

Fig. S3. A proposed mechanism of the hydrolysis of OSC-GCDIs..........S6

Fig. S4. MALDI-TOF MS analysis of HSA incubated with OSC-GCDI(P)..........S7

Fig. S5. The release profiles of GOC from the OSC-GCDIs conjugated to human serum albumin (HSA)..........S9

Fig. S6. Pharmacokinetic properties of GOC·TFA in mice and rats..........S10

Fig. S7. Pharmacokinetic properties of OSC-GCDIs in mice..........S11

Fig. S8. Therapeutic efficacy of OS-P, GOC·TFA and non-formulated OSC-GCDI(P) with varying doses in mice infected with A/H1N1 influenza virus. S12

Fig. S9. Preparation of OSC-GCDI(C)/HPMCP and OSC-GCDI(P)/HPMCP..........S14

Table S2. Effective permeability through the artificial membrane of GOC and OSC-GCDI(P) with and without HPMCP encapsulation..........S15

Fig. S10. Effect of oral administration frequency on therapeutic efficacy of OSC-GCDI(P)/HPMCP in mice infected with wild-type influenza virus..........S16

Fig. S11. Antiviral efficacy of orally administered OSC-GCDI(P)/HPMCP in mice..........S17

Fig. S12. Effect of oral administration frequency on therapeutic efficacy of OSC-GCDI(P)/HPMCP in mice infected with an OS-resistant influenza virus harboring the H275Y mutation in NA..........S18

Supplemental Experimental Procedures..........S19

Synthetic Procedures..........S23

NMR Spectra of Compounds..........S27

Supplemental References..........S37

Supplemental Data

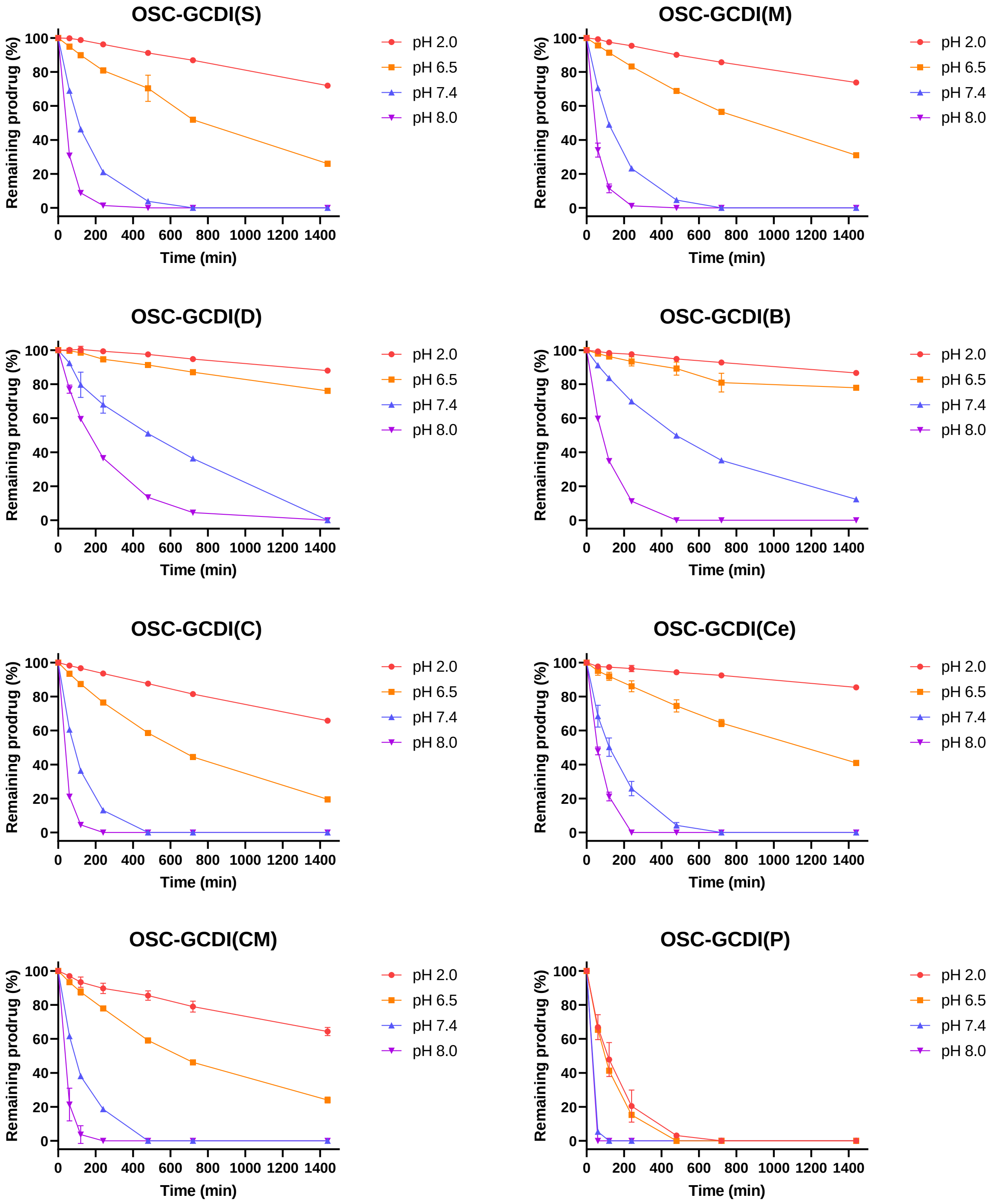

Fig. S1. Degradation profiles of the **OSC-GCDI** prodrugs in phosphate buffer solutions under various pH conditions at 37 ℃. Each data point represents the mean ± SD (*n* = 3).

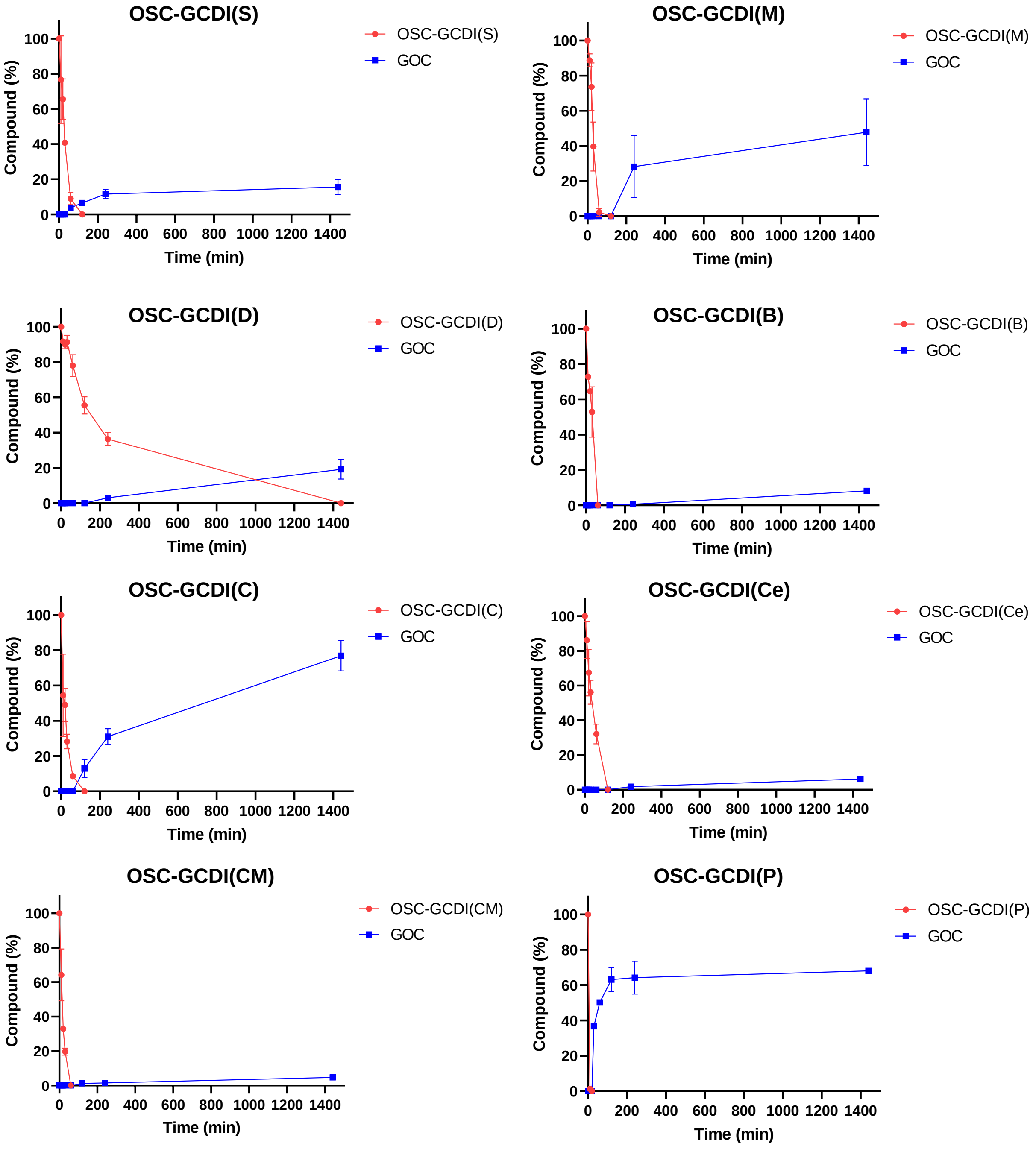

Fig. S2. Degradation profiles of the **OSC-GCDI** prodrugs and the release rate of **GOC** in BALB/c serum at 37 ℃. Each data point represents the mean ± SD (n = 3).

Table S1. *In vitro* microsomal stability of **GOC**, **OSC-GCDI(D)** and **OSC-GCDI(P)**.

		GOC	OSC-GCDI(D)	OSC-GCDI(P)
$T_{1/2}$ (min) [a]	Rat liver microsome [b]	6930	385	17.7
	pH 7.4 phosphate buffer [c]	n.t. [d]	495	14.1
CL_{int} (µL/mg/mg) [e]		0.200	3.60	78.2

[a] Determined by HPLC analysis of the remaining **GOC** or **OSC-GCDI** prodrugs at various time points. The calculation of the half-lives was based on the assumption of pseudo-first order kinetics of the degradation. [b] Examined in Sprague Dawley rat microsomes at a prodrug concentration of 100 µM at 37 °C. [b] Examined in phosphate buffered saline (50 mM phosphate, 154 mM ionic strength, 37 °C). [d] Not tested. [e] Intrinsic clearance.

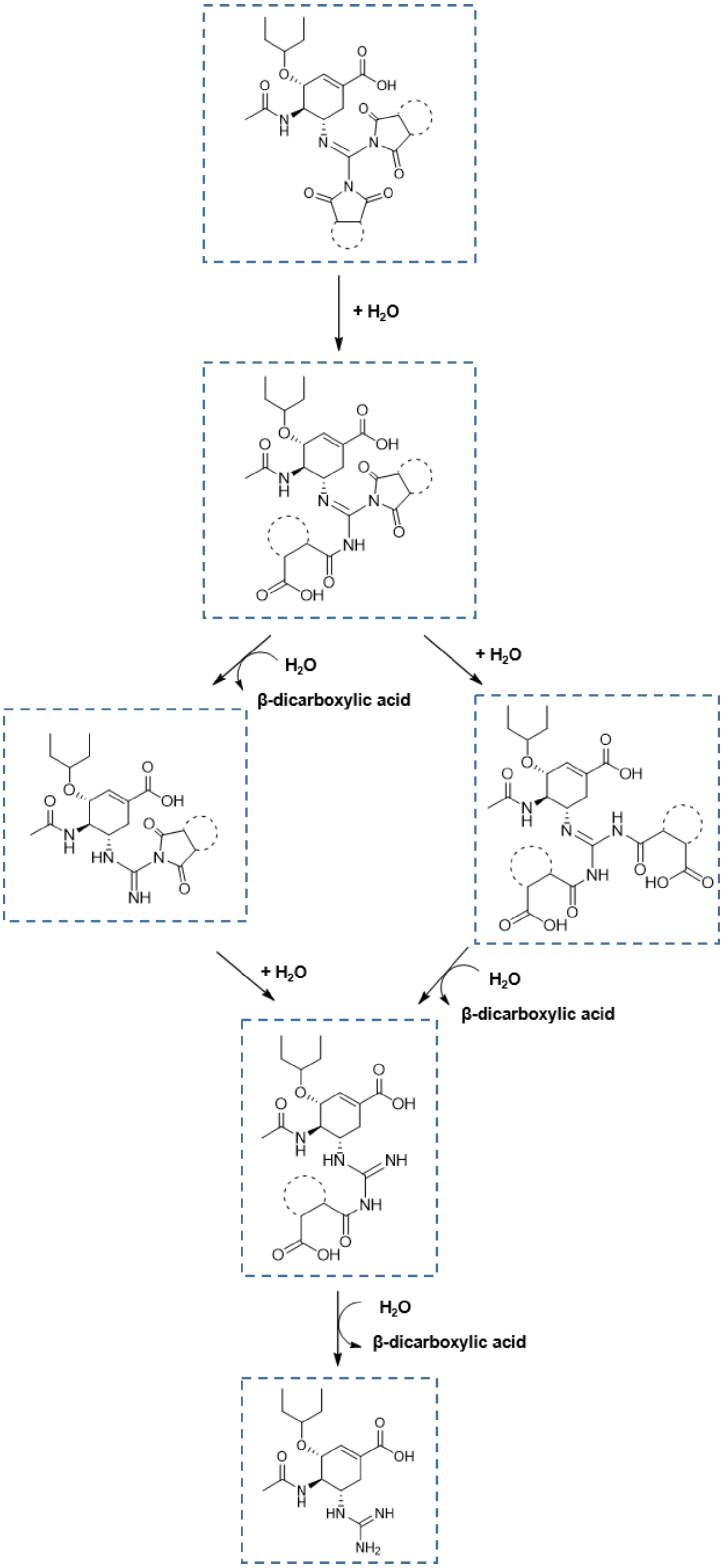

Fig. S3. A proposed mechanism of **OSC-GCDI** hydrolysis into **GOC**. Predicted possible intermediates are indicated as dashed blue squares.

a

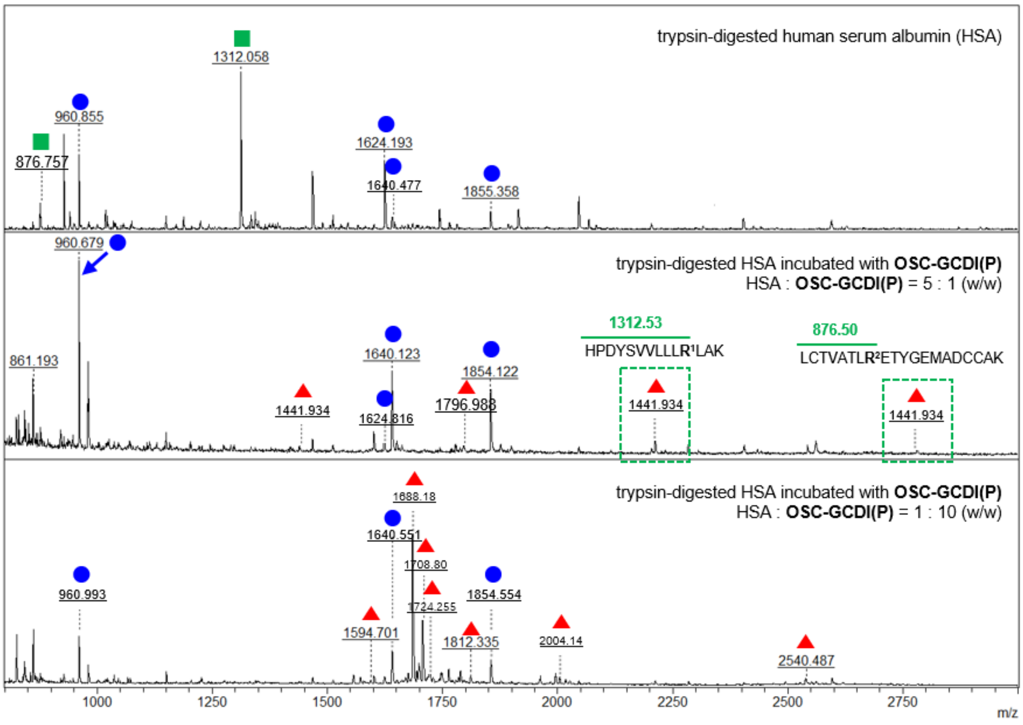

b

Observed *m/z*	Calculated *m/z* of $[M+H]^+$	Suggested Sequence
1441.9	1441.1	FGE**R**^{1}AFK
1594.8	1594.8	CASLQ**K**^{3}FGER
1688.2	1688.3	YLYEIA**R**^{2}R
1708.8	1708.9	FP**K**^{3}AEFAEVSK
1724.7	1724.8	CASLQ**K**^{1}FGER
1797.0	1797.7	AACLLP**K**^{3}LDELR
1812.8	1812.8	F**K**^{1}DLGEENFK
2004.1	2004.0	Y**K**^{3}AAFTECCQAADK
2211.1	2210.9	HPDYSVVLLL**R**^{1}LAK
2540.2	2540.6	AAFTECCQAAD**K**^{1}AACLLPK
2781.7	2782.6	LCTVATL**R**^{2}ETYGEMADCCAK

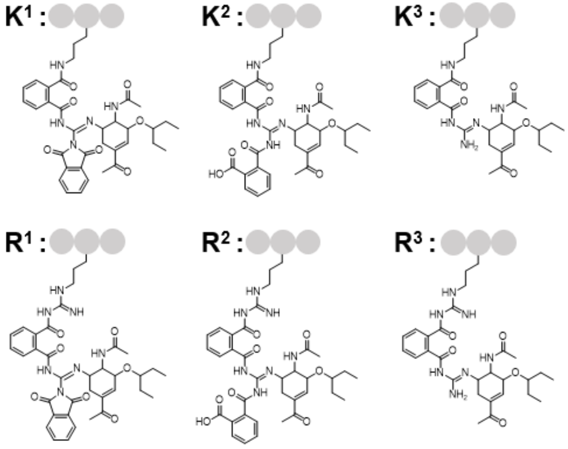

Fig. S4. MALDI-TOF MS analyses of trypsin-digested human serum albumin (HSA) incubated with OSC-GCDI(P). (**a**) MALDI-TOF MS spectra of trypsin-digested human serum albumin (HSA) only (up), HSA incubated with a limited amount of **OSC-GCDI(P)** (middle; HSA : **OSC-GCDI(P)** = 5 : 1 (w/w)), and HSA incubated with an excess amount of **OSC-GCDI(P)** (bottom; HSA : **OSC-GCDI(P)** = 1 : 10 (w/w)). Non-conjugated sequences derived from HSA are indicated as blue circles and green squares. **OSC-GCDI(P)**-conjugated sequences are indicated as red triangles. The green square peaks of HSA only were shifted to the red triangle peaks with dashed green squares by incubation with **OSC-GCDI(P)**. (**b**) Identification of some representative peaks in the spectra. Suggested structures of the covalent linkage between the sequence and **OSC-GCDI(P)** are shown.

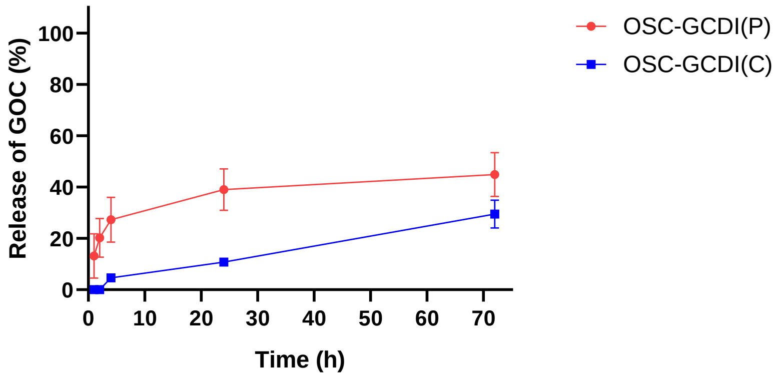

Fig. S5. The release profiles of **GOC** from the **OSC-GCDI**s conjugated to human serum albumin (HSA) at 37 ℃. Each data point represents the mean ± SD (*n* = 3).

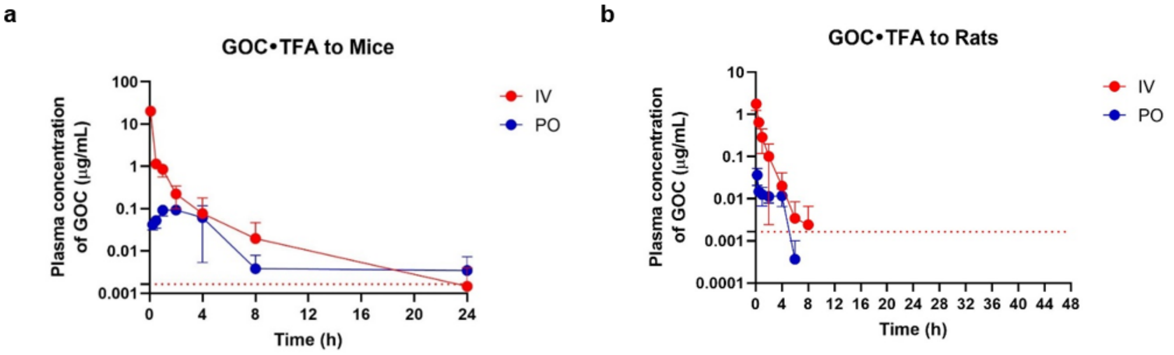

Parameter	IV (5 mg/kg)	PO (5 mg/kg)
T_{max} (h)	NA	2.00 ± 1.73
C_{max} (μg/mL)	NA	0.11 ± 0.01
$T_{1/2}$ (h)	1.80 ± 1.56	4.00 ± 3.90
AUC_{last} (μg·h/mL)	8.46 ± 0.86	0.82 ± 0.79
AUC_{∞} (μg·h/mL)	8.47 ± 0.87	0.70 ± 0.11
CL (L/h/kg)	0.44 ± 0.04	NA
V_{ss} (L/kg)	0.22 ± 0.19	NA
MRT_{last} (h)	0.51 ± 0.46	4.12 ± 1.81
MRT_{∞} (h)	0.54 ± 0.5	6.16 ± 5.12
F_t (%)	NA	9.69

Parameter	IV (5 mg/kg)	PO (10 mg/kg)
T_{max} (h)	NA	0.25 ± 0
C_{max} (μg/mL)	NA	0.04 ± 0.02
$T_{1/2}$ (h)	0.85 ± 0.22	3.57 ± 2.25
AUC_{last} (μg·h/mL)	1.24 ± 0.39	0.06 ± 0.00
AUC_{∞} (μg·h/mL)	1.24 ± 0.39	0.07 ± 0.01
CL (L/h/kg)	3.16 ± 0.9	NA
V_{ss} (L/kg)	2.09 ± 0.67	NA
MRT_{last} (h)	0.68 ± 0.32	2.02 ± 0.72
MRT_{∞} (h)	0.71 ± 0.35	5.1 ± 3.36
F_t (%)	NA	2.36

Fig. S6. Pharmacokinetic properties of GOC·TFA in mice and rats. (**a**) Plasma concentrations and the pharmacokinetic parameters of **GOC** after administration of **GOC·TFA** in mice. ICR mice were administered with **GOC·TFA** intravenously (red circles) or orally (blue circles) at a dose of 5 mg/kg. Blood sample collection via tail vein began at 0.083 h when intravenously administered or at 0.25 h when orally administered. Collection continued at 0.5, 1, 2, 4, 8, and 24 h after treatment in both bases. Plasma concentration of **GOC** from three mice was determined by LC-MS/MS. The **GOC** concentration equivalent to an EC_{50} value (0.005 μM) against PR8 (A/H1N1) in MDCK cells is indicated by a red dashed line. Plasma concentrations of **GOC** from three mice were determined by LC-MS/MS. (**b**) Plasma concentrations and the pharmacokinetic parameters of **GOC** after administration of **GOC·TFA** into rats. SD rats were administered with **GOC·TFA** intravenously (red circles) or orally (blue circles) at doses of 5 and 10 mg/kg, respectively (n = 3). Blood sample collection via tail vein began at 0.083 h when intravenously administered or at 0.25 h when orally administered. Collection continued at 0.5, 1, 2, 4, 6, 8, 24, 32 and 48 h after treatment in both cases. The GOC concentration in plasma equivalent to the EC_{50} value (0.005 μM) against PR8 (A/H1N1) in MDCK cells is indicated by red dashed lines. Plasma concentrations of **GOC** from three rats were determined by LC-MS/MS. The following pharmacokinetic parameters are defined; T_{max}, time point indicating maximum concentration; C_{max}, maximum plasma concentration; $T_{1/2}$, terminal half-life; AUC_{last}, area under the concentration-time curve to the last measurable time point; AUC_{∞}, area under the concentration-time curve extrapolated to infinity; CL, clearance; V_{ss}, steady-state volume of distribution; MRT_{last}, mean residence time within the detection period; MRT_{∞}, mean residence time extrapolated to infinity; and F_t, oral bioavailability ((AUC_{last} at oral administration/AUC_{last} at intravenous administration) × 100%). NA, not applicable; NC, not calculated. Values are presented as means ± SD from three different samples.

a

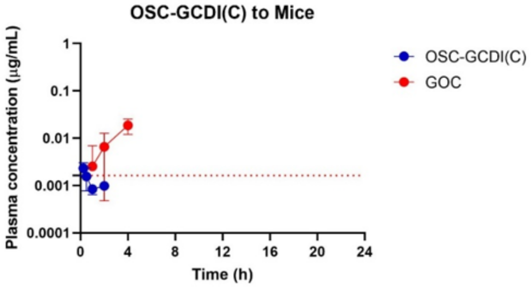

Parameter	OSC-GCDI(C)	GOC
T_{max} (h)	0.25 ± 0.00	4.00 ± 0.00
C_{max} (μg/mL)	0.019 ± 0.007	0.03 ± 0.01
$T_{1/2}$ (h)	3.06 ± 3.06	NC
AUC_{last} (μg·h/mL)	0.002 ± 0.001	0.04 ± 0.00
AUC_{∞} (μg·h/mL)	0.007 ± 0.004	NC
CL (L/h/kg)	NA	NA
V_{ss} (L/kg)	NA	NA
MRT_{last} (h)	0.92 ± 0.14	2.92 ± 1.69
MRT_{∞} (h)	4.70 ± 4.45	NC
F_t (%)	NC	0.48

b

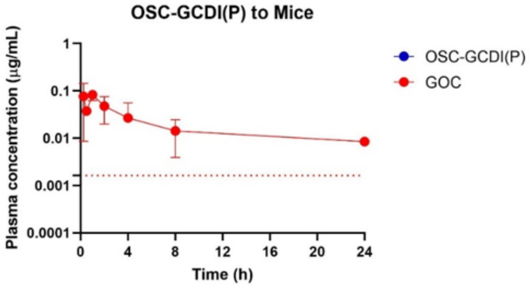

Parameter	OSC-GCDI(P)	GOC
T_{max} (h)	NC	0.75 ± 0.43
C_{max} (μg/mL)	NC	0.14 ± 0.05
$T_{1/2}$ (h)	NC	6.93 ± 0.91
AUC_{last} (μg·h/mL)	NC	0.51 ± 0.24
AUC_{∞} (μg·h/mL)	NC	0.69 ± 0.18
CL (L/h/kg)	NA	NA
V_{ss} (L/kg)	NA	NA
MRT_{last} (h)	NC	5.89 ± 2.38
MRT_{∞} (h)	NC	11.49 ± 3.65
F_t (%)	NC	5.95

Fig. S7. Pharmacokinetic properties of OSC-GCDIs in mice. (**a**) Plasma concentrations and the pharmacokinetic parameters of **OSC-GCDI(C)** (blue circles) and its metabolite **GOC** (red circles) in three different mice orally administered with **OSC-GCDI(C)**. (**b**) Plasma concentrations and the pharmacokinetic parameters of **OSC-GCDI(P)** (blue circles; below detection limit) and its metabolite **GOC** (red circles) in three different mice orally administered with **OSC-GCDI(P)**. The **GOC** concentration in plasma equivalent to an EC_{50} value (0.005 μM) against PR8 (A/H1N1) in MDCK cells is indicated by red dashed lines. The following pharmacokinetic parameters are defined: T_{max}, time point indicating maximum concentration; C_{max}, maximum plasma concentration; $T_{1/2}$, terminal half-life; AUC_{last}, area under the concentration-time curve to the last measurable time point; AUC_{∞}, area under the concentration-time curve extrapolated to infinity; CL, clearance; V_{ss}, steady-state volume of distribution; MRT_{last}, mean residence time within the detection period; and MRT_{∞}, mean residence time extrapolated to infinity. NA, not applicable; NC, not calculated. Values are presented as means ± SD from three different mouse samples.

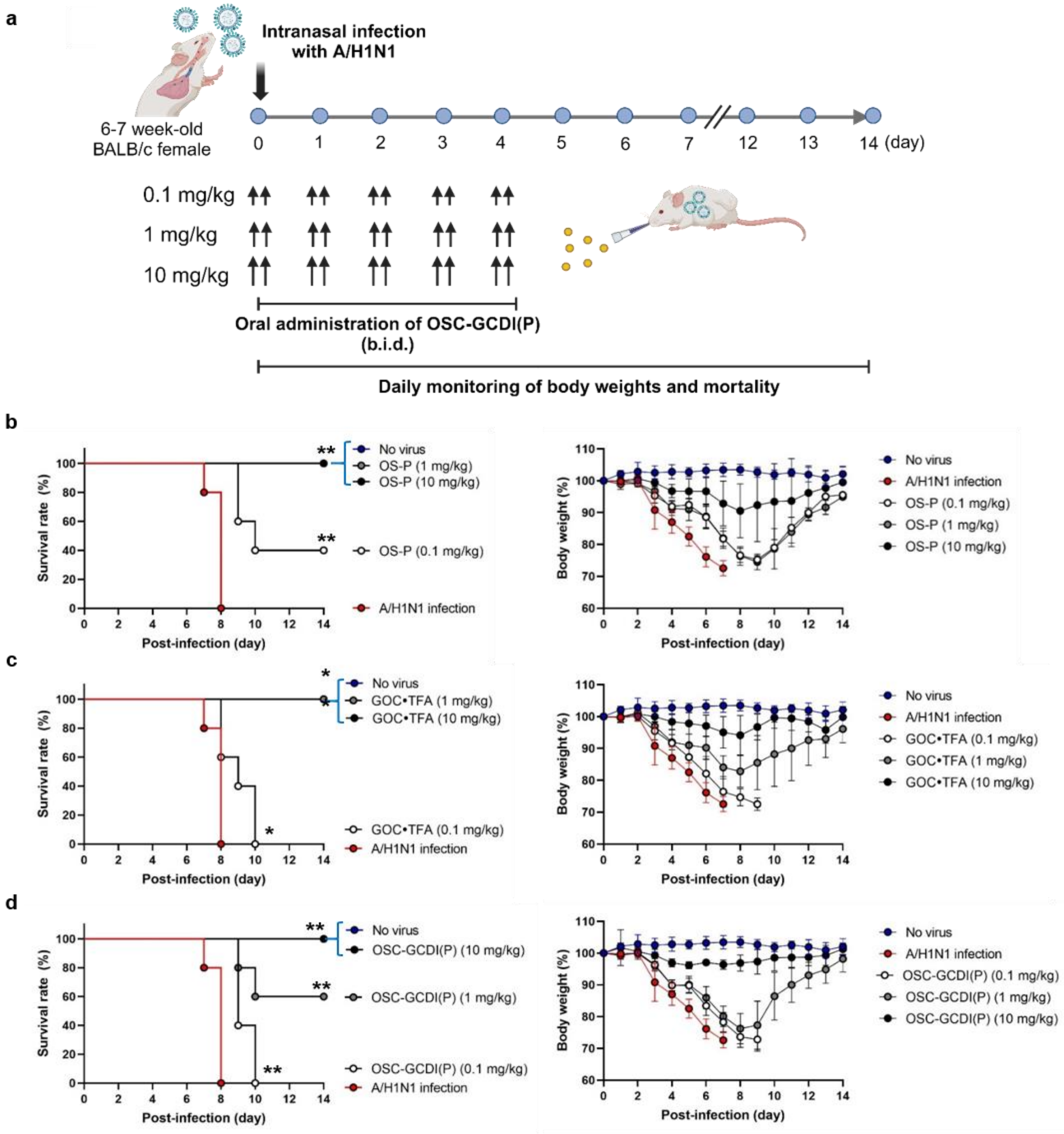
a
Intranasal infection with A/H1N1
6-7 week-old BALB/c female
0 1 2 3 4 5 6 7 12 13 14 (day)
0.1 mg/kg
1 mg/kg
10 mg/kg
Oral administration of OSC-GCDI(P) (b.i.d.)
Daily monitoring of body weights and mortality
b
Survival rate (%)
Post-infection (day)
No virus
OS-P (1 mg/kg)
OS-P (10 mg/kg)
OS-P (0.1 mg/kg)
A/H1N1 infection
Body weight (%)
c
GOC•TFA (1 mg/kg)
GOC•TFA (10 mg/kg)
GOC•TFA (0.1 mg/kg)
d
OSC-GCDI(P) (10 mg/kg)
OSC-GCDI(P) (1 mg/kg)
OSC-GCDI(P) (0.1 mg/kg)

Fig. S8. Therapeutic efficacy of OS-P, GOC·TFA and non-formulated OSC-GCDI(P) with varying doses in mice infected with A/H1N1 influenza virus. (**a**) Schematic representation illustrating the regimen for oral administration of antiviral agents into A/H1N1 influenza virus-infected mice. Six to seven weeks old female BALB/c mice were orally treated with increasing doses of **OSC-GCDI(P)** (0.1, 1 and 10 mg/kg) using **OS-P** and **GOC·TFA** as controls (*n* = 5 per group). Four hours later, mouse-adapted PR8 (maPR8) was intranasally given to the mice. The same doses of each compound were orally administered again four hours post-infection. This twice-a-day (b.i.d.) administration continued for the next four days. Body weight changes and mortality were recorded daily for 14 days. Mock-infected ('No virus') and maPR8-infected groups ('A/H1N1 infection') served as controls. (**b**) Body weight changes (left) and survival rates (right) of maPR8-infected mice after **OS-P** administration. (**c**) Body weight changes (left) and survival rates (right) of maPR8-infected mice after **GOC**·TFA administration. (**d**) Body weight changes (left) and survival rates (right) of maPR8-infected mice after **OSC-GCDI(P)** administration. In panels (**b**) to (**d**), blue and red represent mock infection and maPR8 virus infection, respectively. White, grey and block circles represent 0.1, 1 and 10 mg/kg compound treatments, respectively, for five days into maPR8 virus-infected mice. Mice with a body weight reduction exceeding 30% were euthanized and counted as dead. In the left panels, values are presented as means ± SD from five mice. In the right panels, groups with overlapping survival rate curve are indicated with blue brackets. Statistical significance was determined by comparing time-course survival rates to the maPR8-infected group. *, $P < 0.05$; **, $P < 0.01$.

a

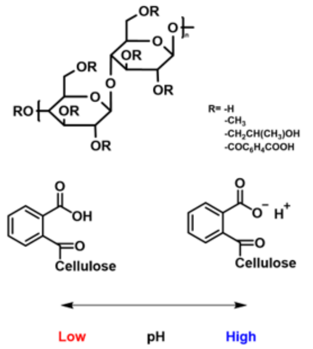

b

	Drug content (μg/mg)	Encapsulation efficiency (%)
**OSC-GCDI(C)**	327±38	31±4
**OSC-GCDI(P)**	503±21	66±3

$$\text{Drug content } (\mu g/mg) = \frac{\text{Mass of drug loaded in particles}}{\text{Mass of particles}}$$

$$\text{Encapsulation efficiency } (\%) = \frac{\text{Mass of drug loaded in particles}}{\text{Mass of drug in the preparation solution}} \times 100$$

c

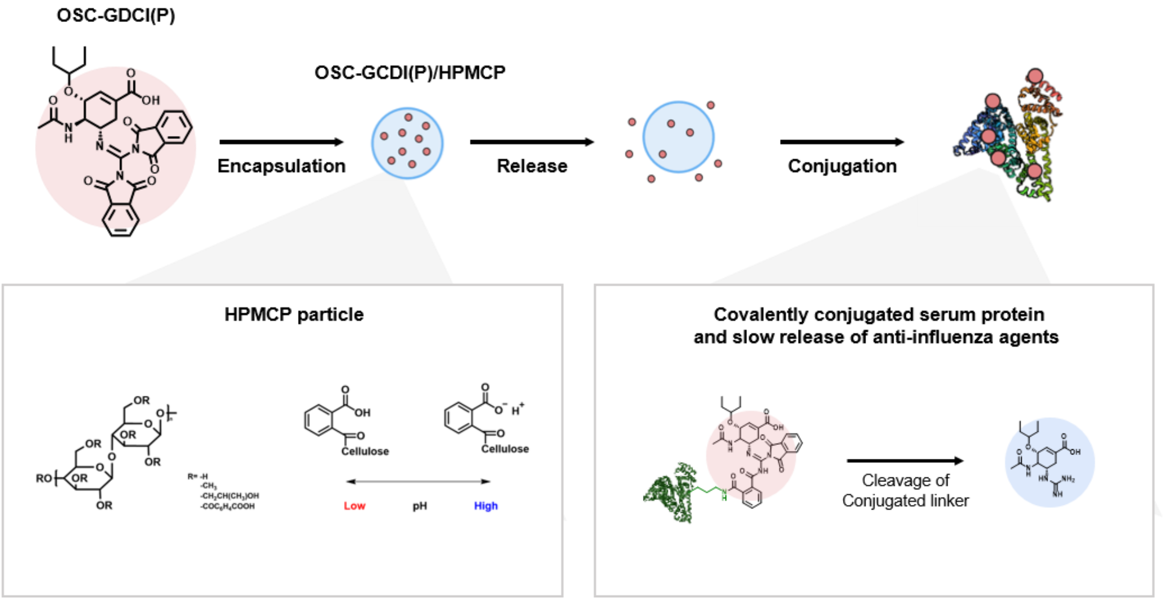

Fig. S9. (**a**) Chemical structures and ionization of hypromellose phthalate (HPMCP) (**b**) Drug contents and encapsulation efficiency of **OSC-GCDI(C)**/HPMCP and **OSC-GCDI(P)**/HPMCP microparticles prepared by coprecipitation (**c**) A schematic diagram of **OSC-GCDI(P)** released from HPMCP particles and binding to serum proteins.

Table S2. Effective permeability through the artificial membrane of **GOC** and **OSC-GCDI(P)** with and without HPMCP encapsulation.

	**GOC**	**GOC**/HPMCP	**OSC-GCDI(P)**	**OSC-GCDI(P)**/HPMCP
P_e x 10^{-6} (cm/s)	n.d.	n.d.	3.88±0.31	2.63±0.20

The data are presented as means ± standard deviations (SD) (n = 3). n.d., not detected.

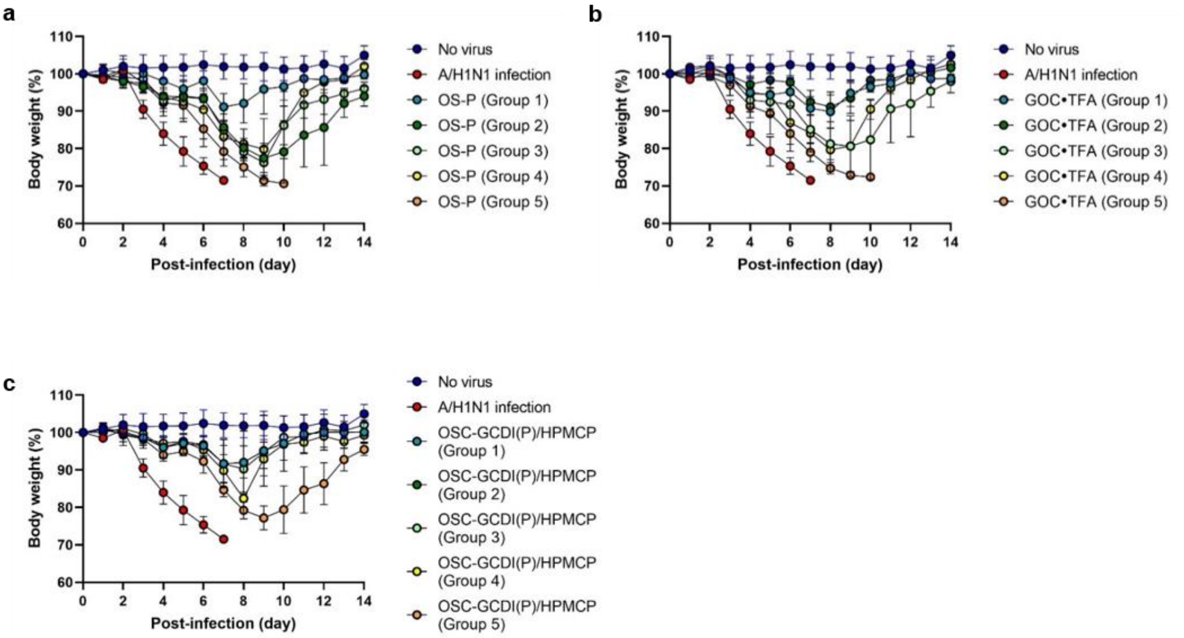

Fig. S10. Effect of oral administration frequency on therapeutic efficacy of OSC-GCDI(P)/HPMCP in mice infected with wild-type influenza virus (**a**) Body weight changes of maPR8-infected mice after **OS-P** administration. (**b**) Body weight changes of maPR8-infected mice after **GOC·TFA** administration. (**c**) Body weight changes of maPR8-infected mice after **OSC-GCDI(P)** administration. In panels (**a**) to (**c**), blue and red circles represent mock infection and maPR8 virus infection, respectively. Sky blue, dark green, bright green, yellow and orange circles represent Groups 1 to 5, respectively. Mice with a body weight reduction exceeding 30% were euthanized and counted as dead. In the left panels, values are presented as means ± SD from five mice. In the right panels, groups with overlapping survival rate curve are indicated with blue brackets. Statistical significance was determined by comparing time-course survival rates to the maPR8-infected group. **, $P < 0.01$.

a

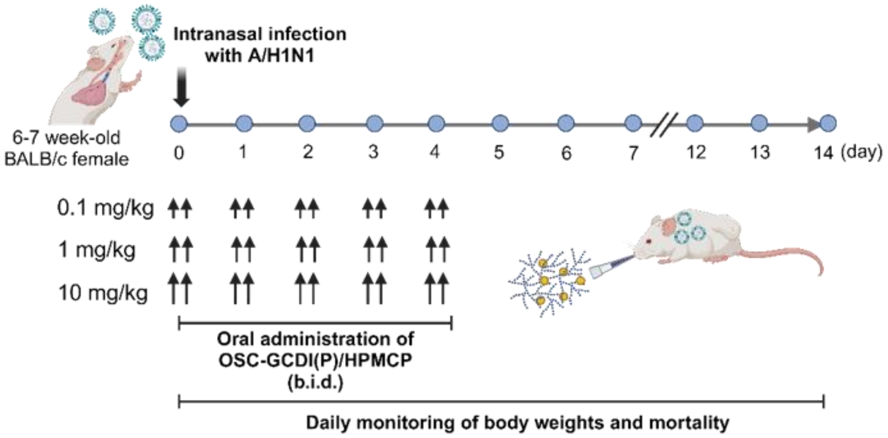

b

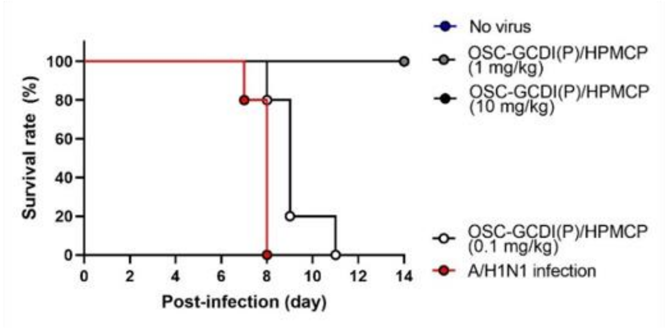

Fig. S11. Antiviral efficacy of orally administered OSC-GCDI(P)/HPMCP in mice. (**a**) Schematic representation illustrating the regimen for oral administration of antiviral agents into A/H1N1 influenza virus-infected mice. Six to seven weeks old female BALB/c mice were orally treated with increasing doses of **OSC-GCDI(P)** at doses of 0.1, 1 and 10 mg/kg (n = 5 per group). (**b**) *In vivo* therapeutic efficacy of **OSC-GCDI(P)** formulated with HPMCP (**OSC-GCDI(P)**/HPMCP) in A/H1N1 virus-infected mice. Female BALB/c mice (six to seven weeks old) were orally treated with escalating doses of **OSC-GCDI(P)**/HPMCP, including 0.1 (white circles), 1 (grey circles) and 10 mg/kg (black circles) (n = 5 per group). Four hours later, A/H1N1 virus (maPR8) was intranasally challenged to the mice. The same doses of **OSC-GCDI(P)**/HPMCP were orally administered again four hours post-infection. The twice-a-day (b.i.d.) administration continued for the next four days. Daily monitoring of body weight changes (left) and survival rates (right) were maintained for 14 days. Mock-infected (blue circles) and maPR8-infected groups (red circles) served as controls. In the right panel, groups with overlapping survival rate curve are indicated with a blue bracket.

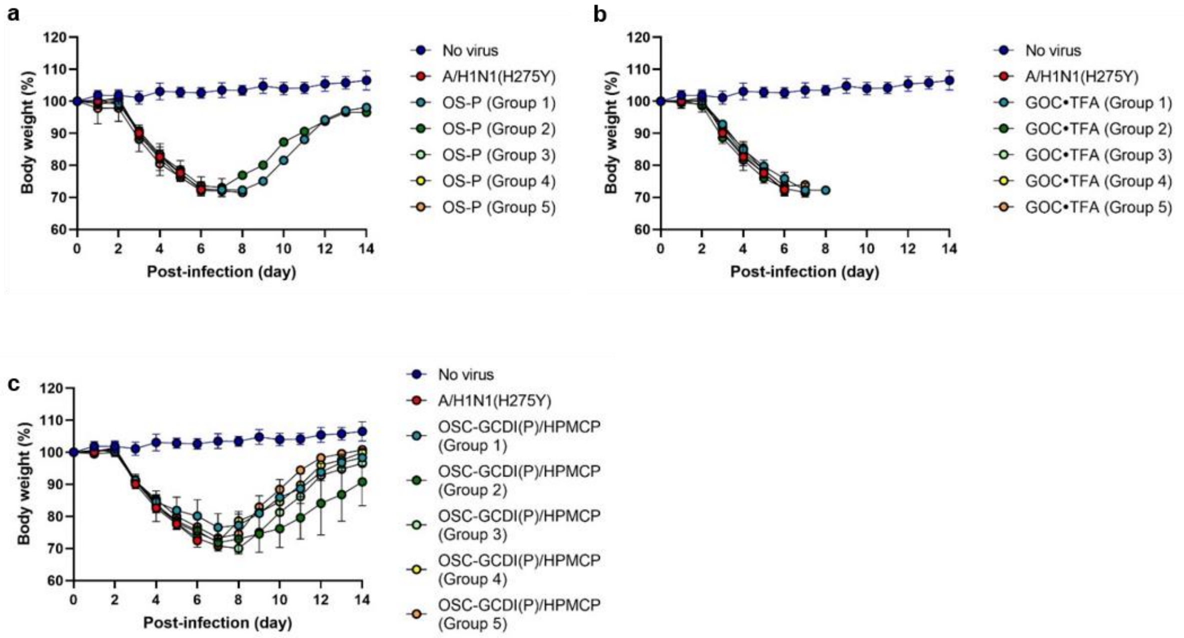

Fig. S12. Effect of oral administration frequency on therapeutic efficacy of OSC-GCDI(P)/HPMCP in mice infected with an OS-resistant influenza virus harboring the H275Y mutation in NA. Female BALB/c mice were orally treated with **OSC-GCDI(P)**/HPMCP at a dose 10 mg/kg, with **OS-P** and **GOC·TFA** serving as controls (n = 5 per group). Four hours later, the **OS**-resistant A/H1N1 virus (rgA/Korea/09/2009Δ53-60(H275Y), referred to as A/H1N1(H275Y)) was intranasally challenged to the mice. Each compound was additionally administered at different intervals: days 1 to 4 post-infection in Group 1, days 2 and 4 in Group 2, day 3 in Group 3 and, day 4 in Group 4. In Group 5, there was no additional treatment after the single administration. Daily recordings of body weight changes and mortality were maintained for 14 days. Mock-infected and A/H1N1(H275Y)-infected groups were included as controls. (**a**) Body weight changes of A/H1N1(H275Y)-infected mice after administration with **OS-P**. (**b**) Body weight changes of A/H1N1(H275Y)-infected mice after administration with **GOC·TFA**. (**c**) Body weight changes of A/H1N1(H275Y)-infected mice after administration with **OSC-GCDI(P).** In panels (**a**) to (**c**), blue and red circles represent mock infection and A/H1N1(H275Y) virus infection, respectively. Sky blue, dark green, bright green, yellow and orange circles represent Groups 1 to 5, respectively. Mice with a body weight reduction exceeding 30% were euthanized and counted as dead. In the left panels, values are presented as means ± SD from five mice. In the right panels, groups with overlapping survival rate curve are indicated with blue brackets. Statistical significance was determined by comparing time-course survival rates to the A/H1N1(H275Y)-infected group. *, $P < 0.05$; **, $P < 0.01$.

Supplemental Experimental Procedures

Lipophilicity.

To assess of octanol-water partition coefficient (log *P*), the solution of each **OSC-GCDI** prodrug (5.0, 10, and 20 mM) was prepared in deionized water (DIW)-saturated *n*-octanol. 1.0 mL of these solutions were then equilibrated at room temperature with an equivalent volume (1.0 mL) of octanol-saturated DIW by vigorous shaking for 1 min. After shaking, the octanol and aqueous phases were separated by centrifugation at 14,000 rpm for 10 min at 4°C. The aqueous phase was collected and filtered by PVDF filter (0.20 µm pore size). The total prodrug concentration in the aqueous phase ($[prodrug]_{aq}$) was determined by HPLC analysis; To assess of octanol-buffer distribution coefficients at pH 7.4 (log $D_{7.4}$), the solution of each prodrug (5.0, 10, and 20 mM) were prepared in sodium phosphate buffer (50 mM phosphate, pH 7.4)-saturated *n*-octanol. 1.0 mL of these solutions were then equilibrated at room temperature with an equivalent volume (1.0 mL) of octanol-saturated sodium phosphate buffer by vigorous shaking for 1 min. After shaking, the octanol and buffer phases were separated by centrifugation at 14,000 rpm for 10 min at 4°C. The aqueous buffer phase was collected and filtered by a polyvinylidene difluoride (PVDF) filter (0.20 µm pore size). The total prodrug concentration in the aqueous buffer phase ($[prodrug]_{pH\ 7.4}$) was determined by HPLC analysis. The total prodrug concentration in the octanol phase ($[prodrug]_{oct}$) was obtained by mass balance. From these data, the octanol-water partition coefficient, $P = [prodrug]_{oct}/[prodrug]_{aq}$, and the octanol-buffer distribution coefficient at pH 7.4, $D_{7.4} = [prodrug]_{oct}/[prodrug]_{pH\ 7.4}$, were determined.

In vitro Parallel Artificial Membrane Permeability Assay (PAMPA)

Membrane permeability of **OSC-GCDI** prodrugs, **GOC**/HPMCP and **OSC-GCDI(P)**/HPMCP were evaluated using Parallel Artificial Membrane Permeability Assay Kit (PAMPA-096). Each prodrug and the supplied permeability controls were dissolved in DMSO at the concentration of 10 mM. Then 25 µL of each prodrug solution was mixed with 475 µL of 50 mM sodium phosphate buffer (pH 6.5) to be used as the test solution. **GOC**/HPMCP and **OSC-GCDI**/HPCMP particles are dissolved in 50 mM sodium phosphate buffer (5% DMSO, pH 6.5) to achieve a final concentration of 200 µM. In the acceptor plate, 300 µL of 50 mM sodium phosphate buffer (pH 7.4) was added. Subsequently, before adding 200 µL of each test solution to the donor plate, 5 µL of 4% lecithin in dodecane was directly applied to the well membrane of the donor plate. Donor plate was carefully placed into acceptor plate and incubate at r.t. for 16 – 24 h. Donor plate was removed and each solution was collected and filtered by a PVDF filter (0.20 µm pore size). The total prodrug concentration in each plate was determined by HPLC analysis. In the case of **OSC-GCDI(P)**, partial degradation occurred due to the instability of **OSC-GCDI(P)** in the buffer. Therefore, the collected solution was further incubated in a 1 M KOH solution for complete hydrolysis. Then, the concentration of **GOC** was quantified instead.

Stability of OSC-GCDIs in buffers

The stability of the GCDI prodrugs were examined by incubating prodrugs in 50 mM sodium phosphate buffer (pH 2.0, 6.5, 7.4, and 8.0) at 37 °C. Briefly, **OSC-GCDI** prodrug was dissolved in DMSO to be 10 mM concentration, then 10 µL of each prodrug solution was mixed with 990 µL of the phosphate buffer. After incubation for 0 h, 1 h, 2 h, 4h, 8h, 12 h, and 24 h, each sample was filtered through a PVDF filter (0.20 µm pore size), and analyzed by HPLC.

Stability of OSC-GCDIs in serum

Stability of the GCDI prodrugs were examined by incubating prodrugs in human and BALB/c serum at 37 °C and 200 μM final concentration. Briefly, each prodrug was dissolved in DMSO to be 20 mM concentration, then 10 μL of each prodrug solution was mixed with 990 μL of serum. At different time intervals, 100 μL of the samples were taken and mixed with 100 μL of MeCN. Samples were centrifuged at 14,000 rpm for 10 min at 4 °C and the supernatant was immediately frozen (−80 °C). After thawing and shaking, the samples were filtered using a polytetrafluoroethylene (PTFE) filter (0.20 μm pore size) and analyzed by HPLC.

Albumin binding of OSC-GCDIs

OSC-GCDI(P) (0.2 mg/mL or 10 mg/mL) was mixed with HSA (1 mg/mL) in 50 mM Tris-HCl buffer (pH 6.8). The sample was shaken for 20 min at room temperature. Unbound **OSC-GCDI(P)** was removed by washing three times with distilled water (DW) using Vivaspin 500 ultrafiltration units (10 kDa cutoff). Following centrifugation, the HSA samples were placed in the condition of 8 M urea, 50 mM Tris-HCl (pH 6.8), and 5 mM DTT at 37 °C for 60 min. Then, 50 mM Tris-HCl (pH 6.8) was added until the urea concentration was less than 1 M. Trypsin Gold was then added to achieve a final protease-to-protein ratio of 1:20 (w/w). The sample was incubated at 37 °C for 60 min. The mass-to-charge ratio of the digested sample was measured by MALDI-TOF (Matrix-Assisted Laser Desorption/Ionization Time-of-Flight) MS using α-cyano-4-hydroxycinnamic acid as the matrix.

Release profiles of GOC from the OSC-GCDI prodrugs conjugated to human serum albumin

OSC-GCDIs were dissolved in Dulbecco's Phosphate-Buffered Saline (DPBS) containing HSA (20 mg/mL) to be concentration of 1 mM, then the mixture was shaken for 10 min at 37 °C. Unbound **OSC-GCDI**s were removed by washing twice with DPBS using Vivaspin 500 ultrafiltration units (10 kDa cutoff). Following centrifugation, the samples were redissolved in 1 mL DPBS. Before incubation at 37 °C, 50 μL sample was taken and mixed with 50 μL of 1 M KOH in methanol solution to completely hydrolyze the conjugates and determine the initial drug concentration bound to HSA. Subsequently, the samples were incubated at 37 °C and after 1, 2, 4, 24, and 72 h incubation, 50 μL of the samples were taken and mixed with 50 μL MeCN. Then, the concentration of the released **GOC** was quantified by HPLC.

***In vitro* microsomal stability**

Sprague Dawley rat microsomes were obtained from Gibco™. NADPH was purchased from Sigma-Aldrich (Munich, Germany). Liver microsomes at 0.5 mg/mL concentration (final volume of 200 μL) were incubated in phosphate buffer (pH 7.4), containing 3 mM $MgCl_2$. **OSC-GCDI** prodrug was dissolved in DMSO to be 40 mM concentration, then each prodrug solution was mixed with prepared buffer to achieve a final concentration of 100 μM (0.25% DMSO). Test compounds were incubated at 37 °C and started by the addition of the 1mM NADPH as cofactor. The samples were taken at 0, 30, 60, and 90 min. The reaction was terminated by addition of ice-cold acetonitrile/methanol (1:1, v/v). After collection, samples were centrifuged (10 min, 16,000 rpm), then the centrifuged supernatant was directly analyzed by using HPLC analysis. Metabolic half-time ($t_{1/2}$) was calculated by using the equation of first order kinetics.

Cells and viruses

Madin-Darby canine kidney (MDCK) cells were purchased from the American Type Culture Collection (ATCC) and cultured in Minimum Essential Medium (MEM; Invitrogen) supplemented with 10% fetal bovine serum (FBS; Atlas Biologicals).

Influenza viruses, namely A/Puerto Rico/8/1934 (PR8; H1N1), A/Hong Kong/8/1968 (HK; H3N2), and B/Lee/1940 (Lee), were acquired from ATCC. A/Korea/2785/2009 carrying the H275Y mutation was obtained from the Korea Centers for Disease Control and Prevention (KCDC), now known as the Korea Disease Control Agency (KDCA). Mouse-adapted PR8 (maPR8) and mouse-infectious oseltamivir-resistant recombinant virus, rgA/Korea/09/2009Δ53-60(H275Y), were graciously provided by Prof. H. J. Kim from Chung-Ang University (Seoul, South Korea) and Korea University (Seoul, South Korea), respectively. A pair of recombinant PR8 viruses, wild-type (rgPR8) and H275Y mutant (rgPR8(H275Y)), were generated by reverse genetics, according to our previous report.[1] The viruses, including PR8, HK, maPR8, rgPR8 and rgPR8(H275Y), were propagated in 10-11-day-old embryonated chicken eggs for 3 days, while the remaining viruses were inoculated in MDCK cells for 3 days. Egg embryonic fluids and cell culture supernatants were collected and filtered using 0.45-μm pore-sized filters to remove debris. The aliquots were stored at -70 °C, and their titers were quantified by plaque assay before use.

***In vitro* antiviral assay and cytotoxicity test**

MDCK cells were seeded on 96-well plates at a density of 3×10^4 cells per well and cultured overnight. The cells were infected with various influenza viruses at a multiplicity of infection (MOI) of 0.001 for 1 h at 33 °C (for PR8, HK, and Lee), or 35 °C (for rgPR8, rgPR8(H275Y), A/Korea/2785/2009 and rgA/Korea/09/2009Δ53-60(H275Y)) for 1 h. Following virus absorption, unbound virus was removed by washing with PBS. The virus-infected cells were then incubated with 3-fold serial dilutions of each compound (ranging from 100 to 0.005 μM) at the same temperatures as during the virus absorption. On day 3 post-infection, cytopathic effect was quantified by assessing cell viability using 3-(4,5-dimethyl-2-thiazolyl)-2,5-diphenyl-2*H*-tetrazolium bromide (MTT; Sigma). The absorbance at 540 nm from mock-infected, DMSO-treated cells was defined as 100% cells viable, while it from virus-infected, DMSO-treated cells was defined as 0% cells viable for each virus-infected case. The fifty percent effective concentration (EC_{50}) was determined by estimating compound concentrations required to improve the viability of virus-infected cells to 50%. Concurrently, mock-infected cells were treated with the same concentrations of each compound for 3 days at 33°C, followed by an MTT assay. The fifty percent cytotoxic concentration (CC_{50}) was calculated by determining the compound concentrations that reduced MDCK cell viability by 50%. The selectivity index (SI) was defined as the ratio of the CC_{50} value and the EC_{50} value.

Preparation of OSC-GCDI/HPMCP microparticles

HPMCP was dissolved in DMSO to achieve a concentration of 50 mg/mL. 150 mg of **OSC-GCDI(P)** was dissolved in 3 mL of the HPMCP solution. The HPMCP solution containing **OSC-GCDI(P)** was slowly dropped into 40 mL of 0.1 M HCl. The mixture was centrifuged (3000 rpm, 4°C, 10 min) to precipitate the HPMCP microparticles and the supernatant was discarded. Afterwards, 30 mL of pure distilled water was added and washed three times. **OSC-GCDI(P)**/HPMCP microparticles were finally obtained through freeze-drying. The **OSC-GCDI(P)** content in the microparticles was determined by HPLC.

Pharmacokinetic analysis

Non-formulated **OSC-GCDI**s and **GOC·TFA** were dissolved in phosphate buffered saline (PBS) containing 0.5% carboxymethylcellulose (CMC) and **OSC-GCDI(P)**/HPMCP was dissolved in water containing 0.5% carboxymethylcellulose (CMC). Three female ICR mice (7-9 weeks old; Orient Bio) were intravenously administered **GOC·TFA** at a dose of 5 mg/kg, or orally treated with **GOC·TFA**, **OSC-GCDI(C)**, **OSC-GCDI(P)**, or **OSC-GCDI(P)**/HPMCP at the same dose (*n* = 3). Blood sample collection commenced at 0.083 h after intravenous administration and at 0.25 h after oral administration, with subsequent collections at 0.5, 1, 2, 4, 8, and 24 h. For pharmacokinetic studies in rats, 8-week-old female Sprague-Dawley rats were administered **GOC·TFA** either intravenously (5 mg/kg) or orally (10 mg/kg), or orally treated with **OSC-GCDI(P)**/HPMCP at a dose of 10 mg/kg (based on the mass of **OSC-GCDI(P)**). Blood sample collection began at the same time points specified earlier and continued to include additional time points at 0.5, 1, 2, 4, 6, 8, 24, 32 and 48 h. The collected samples were centrifuged at 13,000 rpm for 10 min at 4 °C to remove debris. In **GOC·TFA**-treated plasma samples, **GOC** concentration was determined using LC-MS/MS with an HPLC apparatus (Agilent 1260; Agilent) and a mass spectrometer (Agilent 6460; Agilent). For the **OSC-GCDI**-treated samples, concentrations of both **OSC-GCDI** and its metabolite **GOC** were measured. Analyte quantification was achieved by constructing calibration curves through spiking highly purified, concentration-determined **GOC·TFA** (purity, >99%) or **OSC-GCDI**s (purity, >99%).

Pharmacokinetic parameters were estimated using non-compartmental analysis implemented with the Pheonix WonNonlin software (Pharsight Corporation).[2] Key parameters, including time point indicating maximum concentration (T_{max}), maximum plasma concentration (C_{max}), terminal half-life ($T_{1/2}$), area under the concentration-time curve to the last measurable time point (AUC_{last}), or extrapolated to infinity (AUC_{∞}), clearance (CL), steady-state volume of distribution (Vss), mean residence time within the detection period (MRT_{last}) or extrapolated to infinity (MRT_{∞}) were calculated.

In vivo efficacy study

Female BALB/c mice (Orient Bio) aged six to seven weeks old were orally administered with **OS-P**, **GOC·TFA**, **OSC-GCDI(P)** or **OSC-GCDI(P)**/HPMCP at doses of 0.1, 1 and 10 mg/kg (*n* = 5). Four hours later, the mice were intranasally infected with maPR8 at a 50% mouse lethal dose (MLD_{50}) of 10 for an additional 4 h. Subsequently, the mice were orally administered again with each antiviral agent at the same dose. These treatments (*i.e.*, oral, b.i.d., doses of 0.1, 1 and 10 mg/kg) were repeated every day for the next four days. To assess in vivo therapeutic efficacy, both body weight changes and mortality were daily recorded for 14 days after virus challenge. Mock-infected, vehicle (0.5% CMC in PBS)-treated mice and influenza virus-infected, vehicle-treated mice were included as controls.

For the comparison analysis of therapeutic efficacy with varying treatment frequencies, BALB/c mice were orally administered with **OS-P**, **GOC·TFA** or **OSC-GCDI(P)**/HPMCP at a dose of 10 mg/kg (*n* = 5). Four hours later, they were intranasally challenged with either maPR8 or with rgA/Korea/2009Δ53-60, harboring the H275Y mutation in NA, at an MLD_{50} of 5. The mice were then subjected to the same treatment regimen every day or twice a day for an additional 4 days. In separate groups, oral administration was additively conducted once on day 3 or 4 post-infection, or discontinued after the first single administration. Body weight changes and mortality were recorded daily for 14 days after virus challenge. The two groups, comprising mock-infected, vehicle (0.5% CMC in distilled water)-treated mice and influenza virus-infected, vehicle-treated mice, were included as controls.

In all animal experiments related to antiviral efficacy evaluation, mice exhibiting body weight decrease exceeding 30% were euthanized by CO_2. These experiments were conducted in accordance with the guidelines of the Institutional Animal Care and Use Committee, with approval numbers 2023-6D-07-01, 2023-6D-09-03 and 2023-6D-12-01.

Synthetic Procedures

General considerations

^1H and ^{13}C NMR spectra were obtained on a Varian NMR System 500 MHz spectrometer and Bruker System 500 MHz spectrometer (500 MHz for ^1H and 125 MHz for ^{13}C). ^1H chemical shifts were referenced from the chemical shifts of residual solvent peaks (7.26 ppm for $CDCl_3$, 3.31 ppm for CD_3OD). ^{13}C NMR spectra were recorded with complete proton decoupling. ^{13}C chemical shifts were referenced from the chemical shifts of $CDCl_3$ (77.16 ppm) and CD_3OD (49.00 ppm). Recorded spectral data were processed with the MestReNova (Version 14.0.0). Chemical shifts were reported in parts per million and the following abbreviations (or combinations thereof) were used to explain multiplicities: s = singlet, d = doublet, t = triplet, q = quartet, m = multiplet, brs = broad singlet. Coupling constants, *J*, were reported in Hertz unit (Hz). High performance liquid chromatography (HPLC) was performed on the Shimadzu HPLC system equipped with an LC-20AD pump and an SPD-20A UV detector. Agilent Eclipse XDB-C18 (4.6 × 250 mm, 5 µm) column and Agilent ZORBAX 300SB-C18 (9.4 × 250 mm, 5 µm) column were used for analysis. For the mobile phase, Solution A (water with 0.1% v/v trifluoroacetate (TFA)) and Solution B (MeCN with 0.1% v/v TFA) were used as eluents. Mass spectra (MS) were recorded on an Agilent 6120 Quadrupole LCMS System and Thermofisher Scientific (LTQ XL) System using ESI-TOF (electrospray ionization-time of flight) mass spectrometer. Analytical thin layer chromatography (TLC) was carried out on pre-coated glass silica gel plates with F254 indicator, and the spots were visualized under 254 nm UV irradiation and/or staining by $KMnO_4$ solution or ninhydrin solution. Flash column chromatography was performed on silica gel (230–400 mesh) using an appropriate eluent system. Commercially available reagents were obtained from Sigma-Aldrich, United States Biological, Merck, Alfa Aesar or TCI and used without further purification. Parallel Artificial Membrane Permeability Assay (PAMPA) Kit was obtained from BioAssay Systems. Human serum from male AB plasma was obtained from Sigma-Aldrich. BALB/c mouse serum was obtained from Innovative Research. Hypromellose Phthalate was gratefully provided by RICHWOOD TRADING COMPANY.

Ethyl (3R,4R,5S)-4-acetamido-5-((Z)-2,3-bis(tert-butoxycarbonyl)guanidino)-3-(pentan-3-yloxy)cyclohex-1-ene-1-carboxylate) (Boc-protected guanidino oseltamivir)

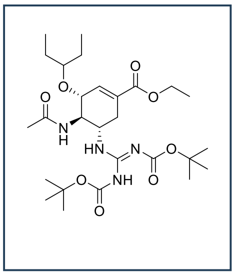

To a solution of oseltamivir phosphate (419 mg, 1.00 mmol, 1.00 equiv.) and *N*,*N'*-di-Boc-1*H*-pyrazole-1-carboxamidine (380 mg, 1.20 mmol, 1.20 equiv.) in acetonitrile (MeCN) (4.00 mL), triethylamine (TEA) (700 µL, 5.00 mmol, 5.00 equiv.) was added, then the reaction mixture was stirred at 50 °C for 18 h. After the reaction, the crude mixture was concentrated under reduced pressure. **Boc-protected guanidino oseltamivir** was afforded (554 mg, >99%) as a white solid after purification by flash column chromatography (SiO_2, hexane/ethyl acetate 3:1 → hexane/ethyl acetate 1:1). ^1H NMR (500 MHz, $CDCl_3$) δ 11.32 (brs, 1H), 8.61 (brs, 1H), 6.85 (d, J = 8.9 Hz, 1H), 6.68 (s, 1H), 4.40 – 4.23 (m, 1H), 4.15 – 3.97 (m, 3H), 3.95 – 3.83 (m, 1H), 3.30 – 3.18 (m, 1H), 2.68 (dd, J = 17.6, 4.3 Hz, 1H), 2.28 (dd, J = 17.6, 8.7 Hz, 1H), 1.81 (s, 3H), 1.56 – 1.23 (m, 22H), 1.16 (t, J = 7.1 Hz, 3H), 0.85 – 0.66 (m, J = 12.1, 7.3 Hz, 6H). Spectral data match those previously reported.[3]

Ethyl (3R,4R,5S)-4-acetamido-5-guanidino-3-(pentan-3-yloxy)cyclohex-1-ene-1-carboxylate trifluoroacetate salt (Guanidino oseltamivir; GO·TFA)

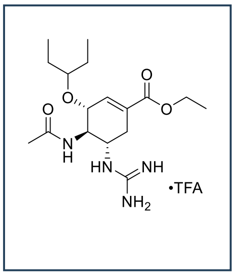

To a stirred solution of Boc-protected guanidino oseltamivir (426 mg, 0.768 mmol) in dichloromethane (DCM) (2.00 mL), trifluoroacetic acid (TFA) (2.00 mL) was added, then the reaction mixture was stirred at ambient temperature for 3 h. The solution was concentrated under reduced pressure and **GO·TFA** was afforded (360 mg, >99%) as a pale yellow solid. ^{1}H NMR (500 MHz, CD_3OD): δ 6.79 (s, 1H), 4.27 – 4.12 (m, 3H), 3.99 – 3.83 (m, 2H), 3.45 – 3.36 (m, 1H), 2.84 (dd, 1H), 2.37 (dd, 1H), 2.00 (s, 3H), 1.58 – 1.43 (m, 4H), 1.28 (t, J = 7.0 Hz, 3H), 0.96 – 0.81 (m, 6H). Spectral data match those previously reported.[4]

(3R,4R,5S)-4-acetamido-5-guanidino-3-(pentan-3-yloxy)cyclohex-1-ene-1-carboxylic acid--2,2,2-trifluoroacetaldehyde *(Guanidino oseltamivir carboxylate; GOC·TFA)*

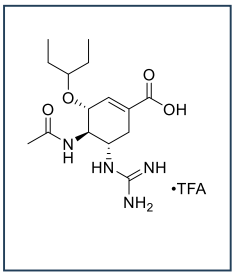

To a stirred solution of GO·TFA in THF (10 mL), aqueous KOH (1 M, 1.00 mL, 1.00 mmol) was added at 0 °C for 17 h. The reaction mixture was acidified to pH 5 with Amberlite IR-120, filtered and rinsed with ethanol. Subsequently, the Boc group was deprotected using 50% DCM/TFA. The solution was concentrated under reduced pressure and purified by HPLC. **GOC·TFA** was afforded (338 mg, >99%) as white solid. ^{1}H NMR (500 MHz, CD_3OD) δ 6.85 (s, 1H), 4.20 (d, 1H), 4.00 – 3.71 (m, 2H), 3.43 (m, 1H), 2.82 (dd, 1H), 2.35 (dd, 1H), 1.99 (s, 3H), 1.64 – 1.45 (m, 4H), 0.92 (m, 6H). Spectral data match those previously reported.[5]

General procedures for the preparation of OSC-GCDIs

To a round-bottom flask, **GOC·TFA** (1.00 mmol; 1.00 equiv.), anhydride (6.00 mmol; 6.00 equiv.) and DMF (5 mL) was added, then *N*,*N*-diisopropylethylamine (DIPEA) (5.00 mmol; 5.00 equiv.) was added. The reaction mixture was stirred at 50 °C for 14 h. After the reaction, the crude mixture was concentrated under reduced pressure and diluted with 50 mL of ethyl acetate and 50 mL of aqueous HCl (0.01 M). The organic layer was further washed with 50 mL of brine twice. The combined organic layers were dried over anhydrous $MgSO_4$, filtered and concentrated under reduced pressure. The product was purified by flash column chromatography (SiO_2, hexane/ethyl acetate).

(3R,4R,5S)-4-acetamido-5-((bis(2,5-dioxopyrrolidin-1-yl)methylene)amino)-3-(pentan-3-yloxy)cyclohex-1-ene-1-carboxylic acid (*OSC-GCDI(S)*)

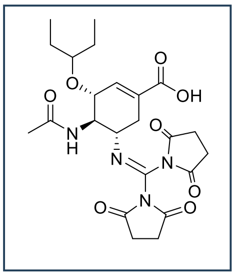

OSC-GCDI(S) was prepared following the general procedure (purification by hexane/ethyl acetate 1:1 → ethyl acetate only, white solid, 275 mg, 56%). ^{1}H NMR (500 MHz, $CDCl_3$) δ 6.85 (s, 1H), 6.17 (d, 1H), 4.91 (s, 1H), 4.40 (s, 1H), 3.29 (p, J = 6.0 Hz, 1H), 3.15 (t, 1H), 2.95 (dd, 1H), 2.77 (m, 6H), 2.44 (dd, 1H), 1.84 (s, 3H), 1.46 (m, 4H), 0.86 (m, 6H). ^{13}C NMR (125 MHz, $CDCl_3$) δ 174.12, 172.83, 170.02, 139.79, 130.45, 128.38, 82.08, 71.17, 59.48, 56.68, 30.08, 28.42, 28.29, 26.47, 25.61, 23.75, 9.77, 9.36. MS (ESI)$^{+}$ calculated for $C_{23}H_{30}N_4O_8$ $[M+H]^{+}$: m/z 491.2, found 491.3.

(3R,4R,5S)-4-acetamido-5-((bis(3-methyl-2,5-dioxopyrrolidin-1-yl)methylene)amino)-3-(pentan-3-yloxy)cyclohex-1-ene-1-carboxylic acid (*OSC-GCDI(M)*)

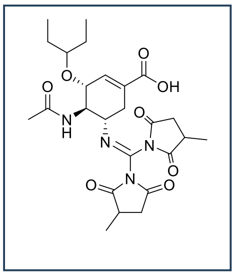

OSC-GCDI(M) was prepared following the general procedure (purification by hexane/ethyl acetate 2:1 → ethyl acetate only, white solid, 270 mg, 52%). ^{1}H NMR (500 MHz, $CDCl_3$) δ 6.90 (s, 1H), 6.10 – 5.94 (d, 1H), 4.97 (s, 1H), 4.52 – 4.30 (s, 1H), 3.30 (t, 1H), 3.12 (d, 1H), 3.05 – 2.89 (m, 4H), 2.54 – 2.38 (m, 3H), 1.84 (s, 3H), 1.55 – 1.20 (m, 12H), 0.89 (m, 6H). ^{13}C NMR (125 MHz, $CDCl_3$) δ 177.66, 173.45, 172.85, 169.95, 139.65, 130.56, 128.38, 82.05, 71.11, 59.45, 56.55, 36.41, 36.35, 36.31, 35.13, 34.93, 34.71, 30.11, 26.40, 25.54, 23.62, 16.43, 9.72, 9.29. MS (ESI)$^+$ calculated for $C_{25}H_{34}N_4O_8$ $[M+H]^+$: m/z 519.2, found 519.2.

(3R,4R,5S)-4-acetamido-5-((bis(3,3-dimethyl-2,5-dioxopyrrolidin-1-yl)methylene)amino)-3-(pentan-3-yloxy)cyclohex-1-ene-1-carboxylic acid (*OSC-GCDI(D)*)

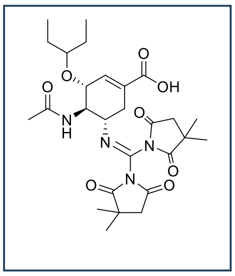

OSC-GCDI(D) was prepared following the general procedure (purification by hexane/ethyl acetate 2:1 → ethyl acetate only, white solid, 372 mg, 68%). ^{1}H NMR (500 MHz, $CDCl_3$) δ 6.87 (s, 1H), 6.26 (d, 1H), 4.98 (s, 1H), 4.38 (s, 1H), 3.30 (t, 1H), 3.20 – 3.05 (m, 1H), 2.98 (dd, 1H), 2.63 (m, 4H), 2.47 (dd, 1H), 1.83 (s, 3H), 1.59 – 1.15 (m, 20H), 0.87 (m, 6H). ^{13}C NMR (125 MHz, $CDCl_3$) δ 172.90, 170.48, 140.35, 130.30, 128.18, 82.25, 71.12, 59.63, 56.66, 43.70, 43.43, 40.42, 26.54, 25.80, 25.68, 23.90, 9.74, 9.48. MS (ESI)$^+$ calculated for $C_{27}H_{38}N_4O_8$ $[M+H]^+$: m/z 547.3, found 547.8.

(3R,4R,5S)-4-acetamido-5-((bis(3-butyl-2,5-dioxopyrrolidin-1-yl)methylene)amino)-3-(pentan-3-yloxy)cyclohex-1-ene-1-carboxylic acid (*OSC-GCDI(B)*)

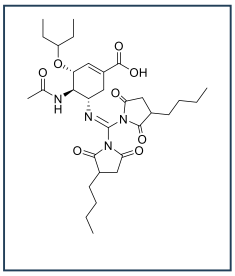

OSC-GCDI(B) was prepared following the general procedure (purification by hexane/ethyl acetate 2:1 → ethyl acetate only, white solid, 253 mg, 42%). ^{1}H NMR (500 MHz, $CDCl_3$) δ 6.90 (s, 1H), 6.08 (d, 1H), 4.96 (s, 1H), 4.40 (td, 1H), 3.30 (t, 1H), 3.13 (dd, 1H), 3.05 – 2.77 (m, 4H), 2.73 (dd, 1H), 2.51 (dd, *J* = 17.0, 4.2 Hz, 3H), 1.84 (s, 3H), 1.70 (dt, 1H), 1.51 (m, 5H), 1.42 – 1.20 (m, 12H), 0.89 (m, 12H). ^{13}C NMR (126 MHz, $CDCl_3$) δ 177.05, 173.54, 172.56, 170.44, 140.23, 130.49, 128.31, 82.17, 71.14, 59.59, 56.70, 40.34, 40.16, 39.99, 34.54, 31.05, 30.98, 30.86, 30.80, 30.13, 29.95, 29.82, 29.13, 29.07, 28.98, 26.53, 25.70, 23.91, 22.44, 13.93, 9.77, 9.45. MS (ESI)$^+$ calculated for $C_{31}H_{46}N_4O_8$ $[M+H]^+$: m/z 603.3, found 603.7.

(3R,4R,5S)-4-acetamido-5-((bis((3aR,7aS)-1,3-dioxooctahydro-2H-isoindol-2-yl)methylene)amino)-3-(pentan-3-yloxy)cyclohex-1-ene-1-carboxylic acid (*OSC-GCDI(C)*)

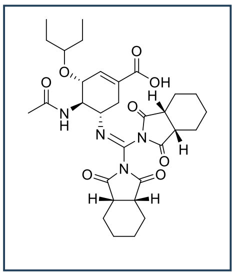

OSC-GCDI(C) was prepared following the general procedure (purification by hexane/ethyl acetate 2:1 → ethyl acetate only, white solid, 497 mg, 83%). ^{1}H NMR (500 MHz, $CDCl_3$) δ 6.89 (s, 1H), 6.06 (d, 1H), 3.45 – 2.75 (m, 7H), 2.55 – 2.33 (m, 1H), 1.85 (m, 13H), 1.68 – 1.21 (m, 15H), 0.89 (m, 6H). ^{13}C NMR (125 MHz, $CDCl_3$) δ 176.60, 175.39, 172.65, 170.27, 139.93, 130.83, 128.27, 82.07, 71.03, 59.57, 56.54, 40.11, 29.78, 26.41, 25.59, 23.92, 23.75, 22.10, 21.96, 9.60, 9.33. MS (ESI)$^+$ calculated for $C_{31}H_{42}N_4O_8$ $[M+H]^+$: m/z 599.3, found 599.4.

(3R,4R,5S)-4-acetamido-5-((bis((3aS,7aR)-5-methyl-1,3-dioxooctahydro-2H-isoindol-2-yl)methylene)amino)-3-(pentan-3-yloxy)cyclohex-1-ene-1-carboxylic acid (*OSC-GCDI(Ce)*)

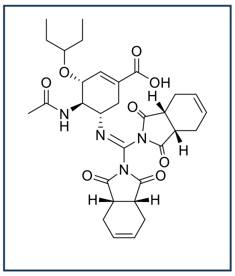

OSC-GCDI(Ce) was prepared following the general procedure (purification by hexane/ethyl acetate 2:1 → ethyl acetate only, white solid, 505 mg, 85%). ^{1}H NMR (500 MHz, $CDCl_3$) δ 6.8 (s, 1H), 6.05 (d, 1H), 6.03 – 5.82 (m, 4H), 3.36 – 2.85 (m, 7H), 2.50 (m, 5H), 2.42 – 2.21 (m, 5H), 1.84 (s, 3H), 1.57 – 1.36 (m, 4H), 0.88 (m, 6H). ^{13}C NMR (125 MHz, $CDCl_3$) δ 177.53, 176.98, 176.44, 173.82, 170.41, 140.33, 128.22, 127.86, 127.75, 127.62, 127.12, 126.68, 82.27, 71.24, 59.58, 56.91, 39.27, 29.97, 26.54, 25.74, 23.79, 23.25, 9.73, 9.46. MS (ESI)$^+$ calculated for $C_{33}H_{46}N_4O_8$ [M+H]$^+$: m/z 627.3, found 595.3.

(3R,4R,5S)-4-acetamido-5-((bis(5-methyl-1,3-dioxooctahydro-2H-isoindol-2-yl)methylene)amino)-3-(pentan-3-yloxy)cyclohex-1-ene-1-carboxylic acid (*OSC-GCDI(CM)*)

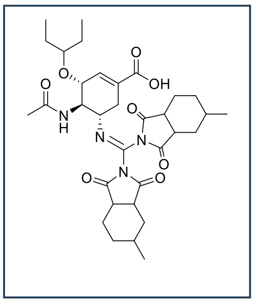

OSC-GCDI(CM) was prepared following the general procedure (purification by hexane/ethyl acetate 2:1 → ethyl acetate only, white solid, 520 mg, 83%). ^{1}H NMR (500 MHz, $CDCl_3$) δ 6.89 (s, 1H), 6.29 (s, 1H), 4.92 (s, 1H), 4.39 (s, 1H), 3.31 (s, 1H), 3.19 (s, 1H), 3.13 – 2.74 (m, 4H), 2.48 (s, 1H), 2.39 – 1.99 (m, 4H), 1.90 (m, 3H), 1.71 – 1.48 (m, 4H), 1.45 (m, 4H), 1.30 – 1.06 (m, 2H), 0.98 – 0.80 (m, 12H). ^{13}C NMR (125 MHz, $CDCl_3$) δ 181.26, 181.04, 180.24, 180.04, 179.86, 140.13, 131.47, 128.41, 82.39, 71.19, 65.93, 44.88, 44.52, 43.15, 42.79, 41.79, 41.10, 36.95, 36.26, 33.84, 32.03, 31.90, 30.66, 28.84, 28.29, 28.17, 26.37, 23.71, 22.33, 22.04, 21.93, 9.73, 9.25. MS (ESI)$^+$ calculated for $C_{31}H_{38}N_4O_8$ [M+H]$^+$: m/z 595.3, found 627.4.

(3R,4R,5S)-4-acetamido-5-((bis(1,3-dioxoisoindolin-2-yl)methylene)amino)-3-(pentan-3-yloxy)cyclohex-1-ene-1-carboxylic acid (*OSC-GCDI(P)*)

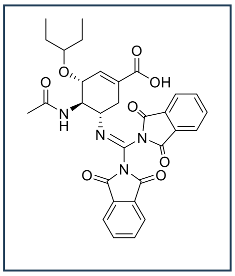

OSC-GCDI(P) was prepared following the general procedure (purification by hexane/ethyl acetate 2:1 → ethyl acetate only, white solid, 223 mg, 38%). ^{1}H NMR (500 MHz, $CDCl_3$) δ 8.04 – 7.66 (m, 8H), 6.85 (d, 1H), 6.22 (d, 1H), 4.60 (td, 1H), 3.42 – 3.21 (m, 2H), 3.11 (dd, 1H), 2.56 (dd, 1H), 1.94 (s, 3H), 1.47 (m, 4H), 0.88 (m, 6H). ^{13}C NMR (125 MHz, $CDCl_3$) δ 172.35, 170.35, 165.37, 140.16, 135.23, 131.24, 130.13, 128.46, 124.49, 82.20, 71.56, 59.70, 56.91, 30.65, 26.51, 25.69, 23.78, 9.79, 9.44. MS (ESI)$^+$ calculated for $C_{31}H_{30}N_4O_8$ [M+H]$^+$: m/z 587.6, found 587.3.

NMR Spectra of Compounds

^{1}H NMR (500 MHz, $CDCl_3$)

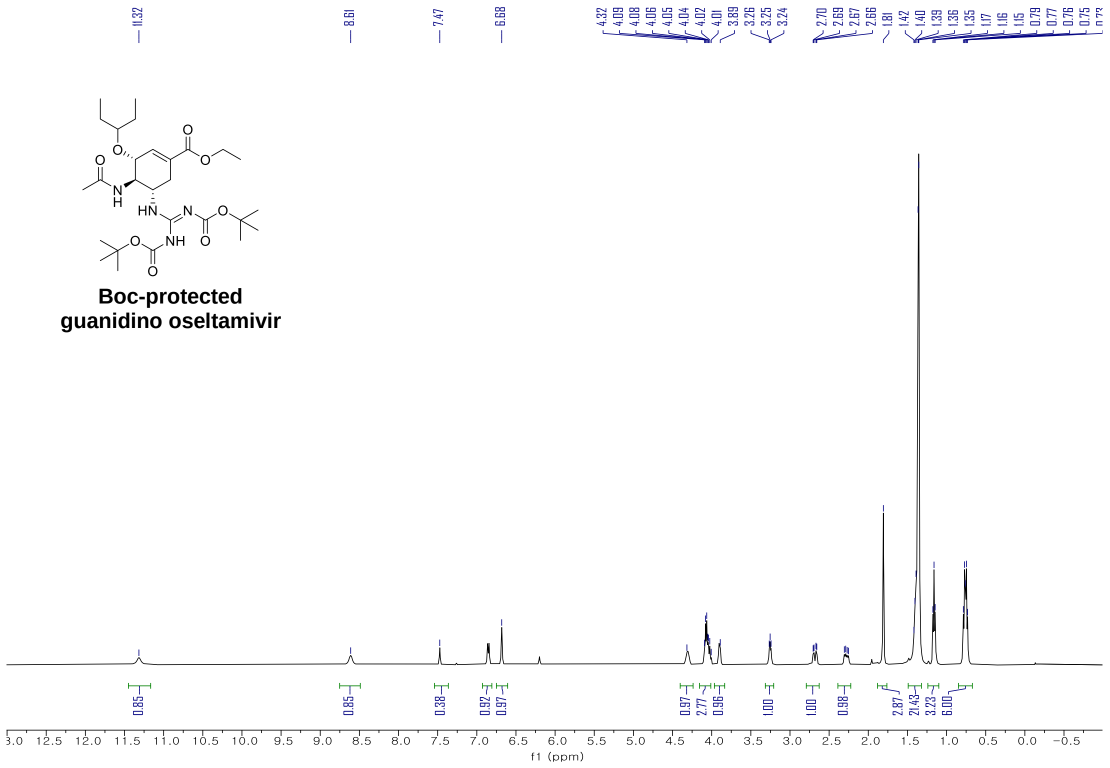

^{1}H NMR (500 MHz, MeOD)

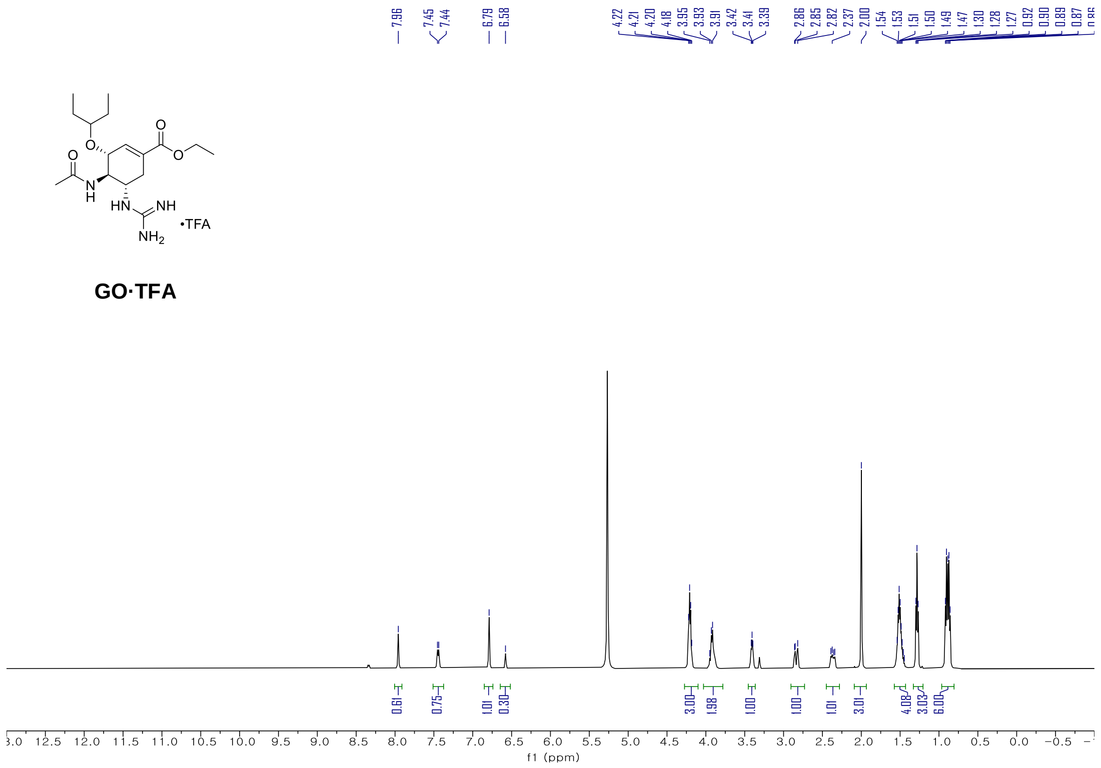

^{1}H NMR (500 MHz, MeOD)

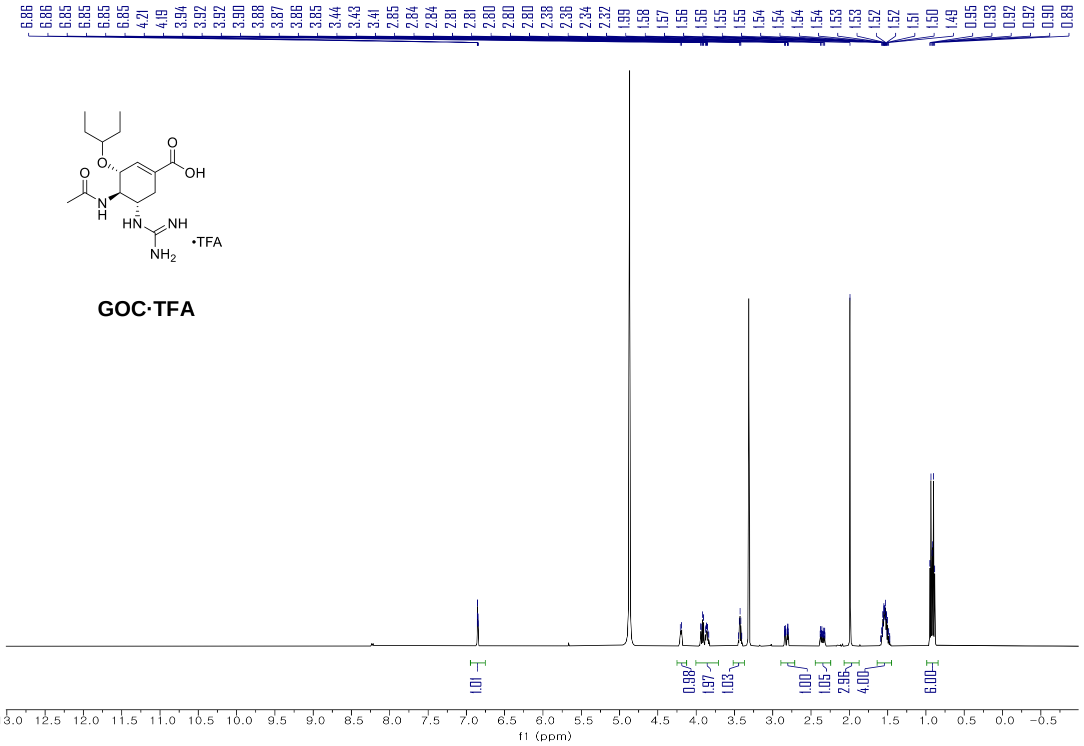

^{1}H NMR (500 MHz, $CDCl_3$)

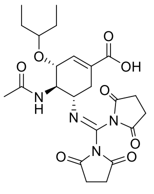

OSC-GCDI(S)

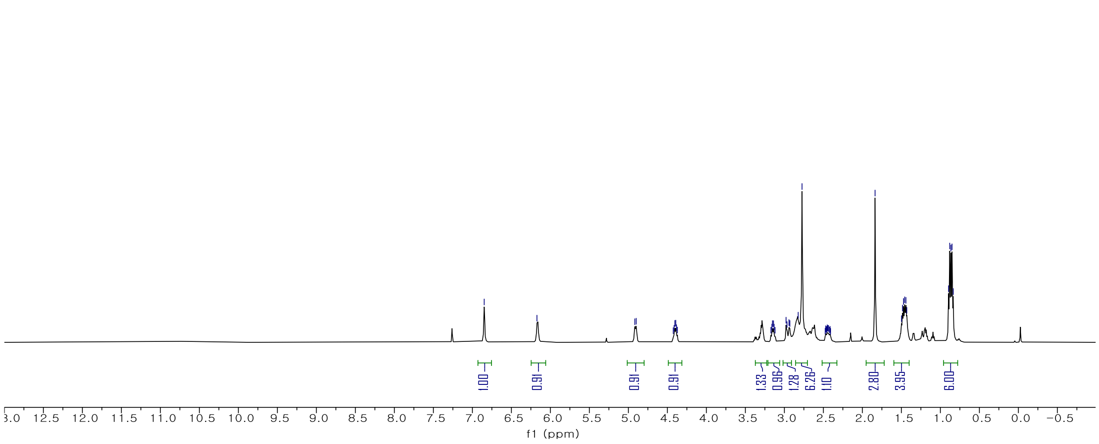

^{13}C NMR (125 MHz, $CDCl_3$)

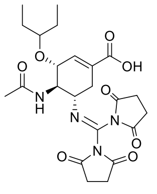

OSC-GCDI(S)

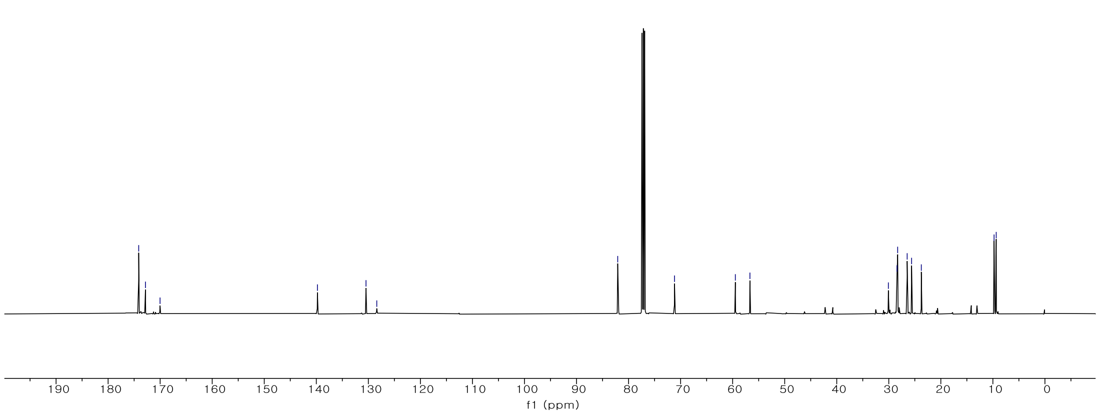

^{1}H NMR (500 MHz, $CDCl_3$)

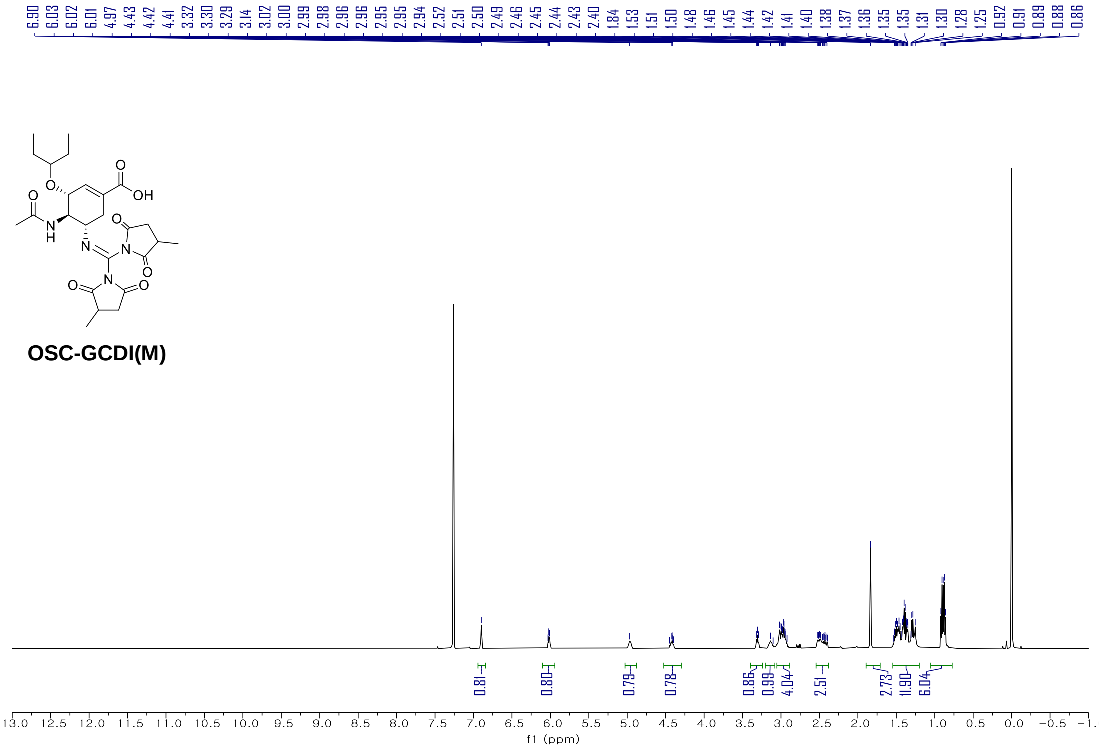

^{13}C NMR (125 MHz, $CDCl_3$)

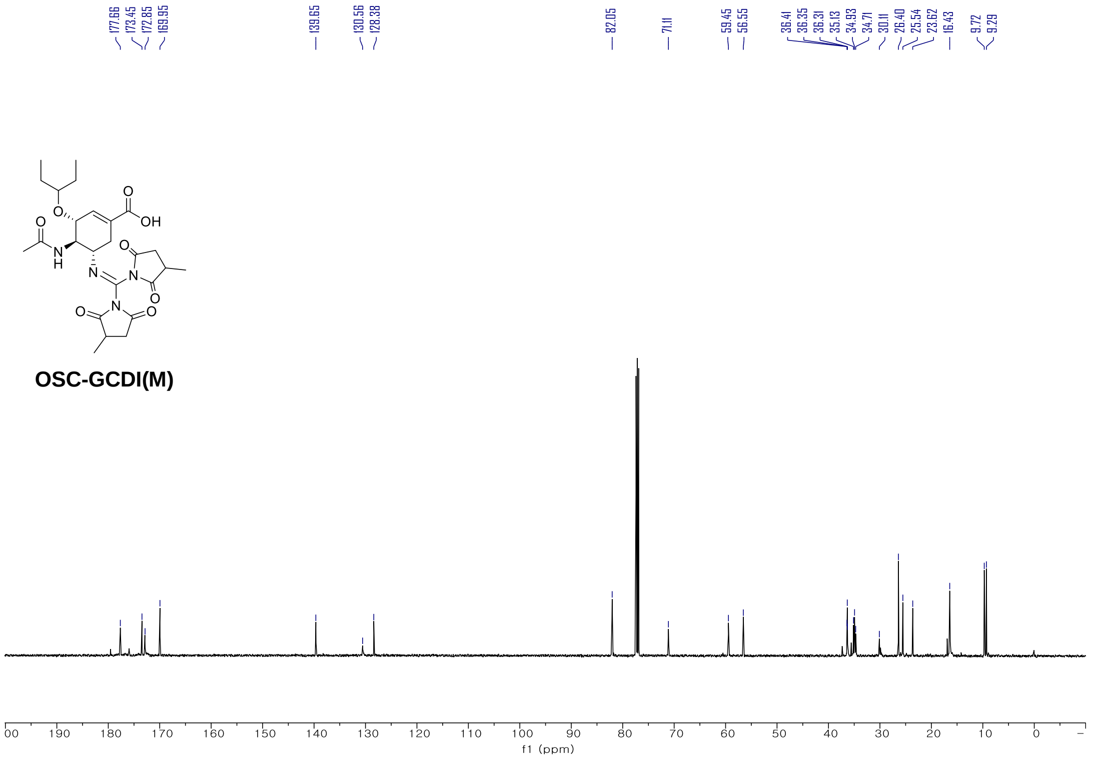

^{1}H NMR (500 MHz, $CDCl_3$)

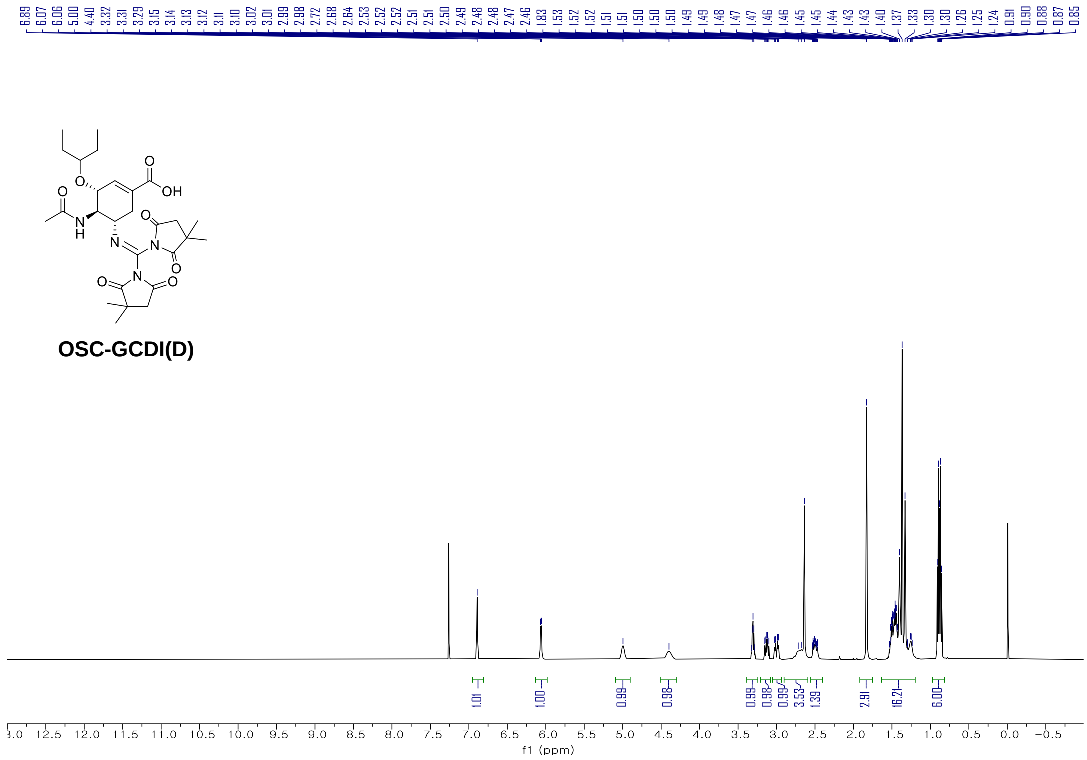

^{13}C NMR (125 MHz, $CDCl_3$)

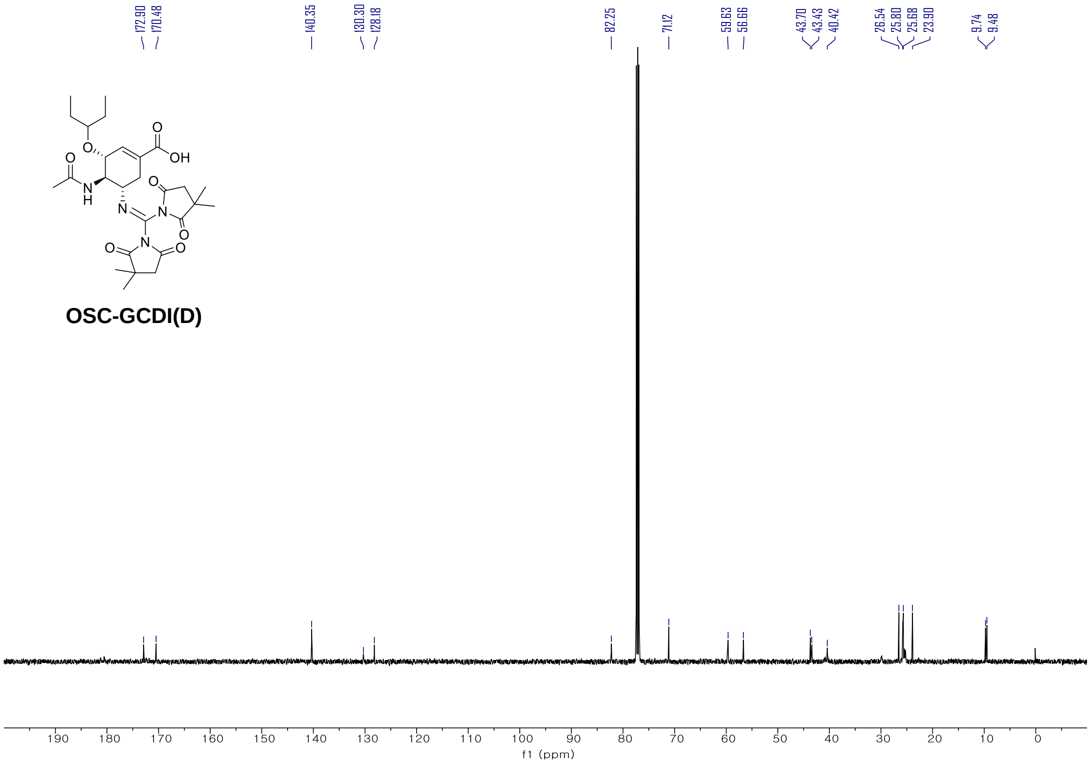

^{1}H NMR (500 MHz, $CDCl_3$)

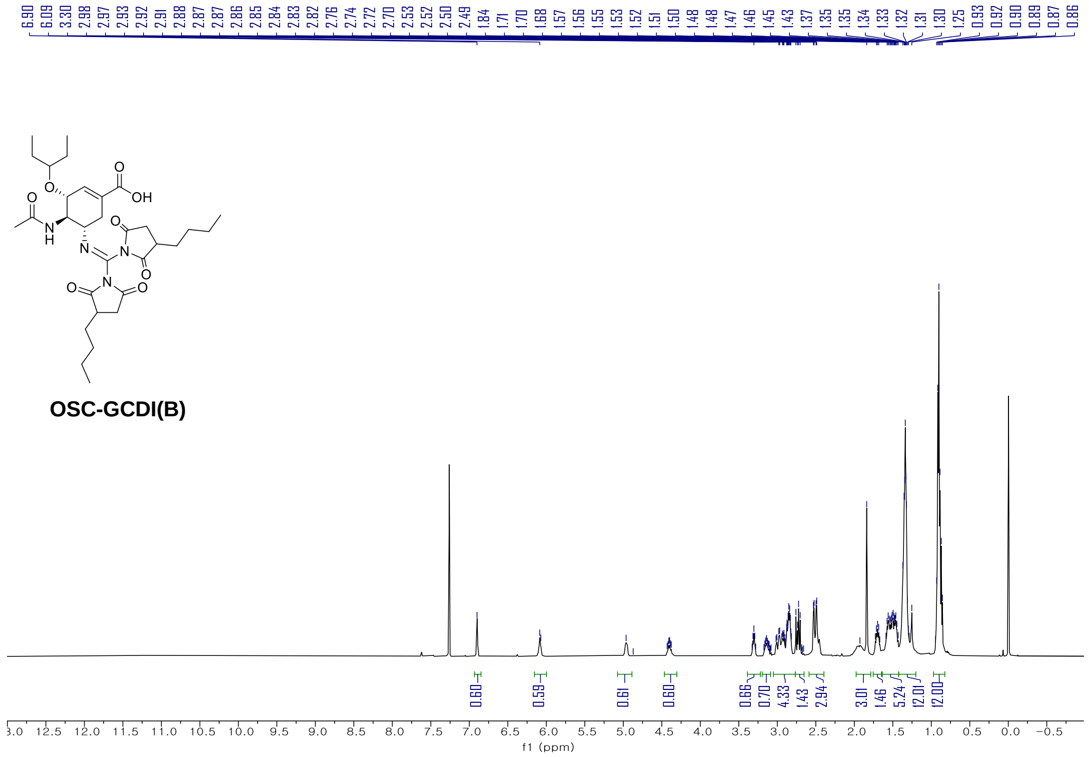

^{13}C NMR (125 MHz, $CDCl_3$)

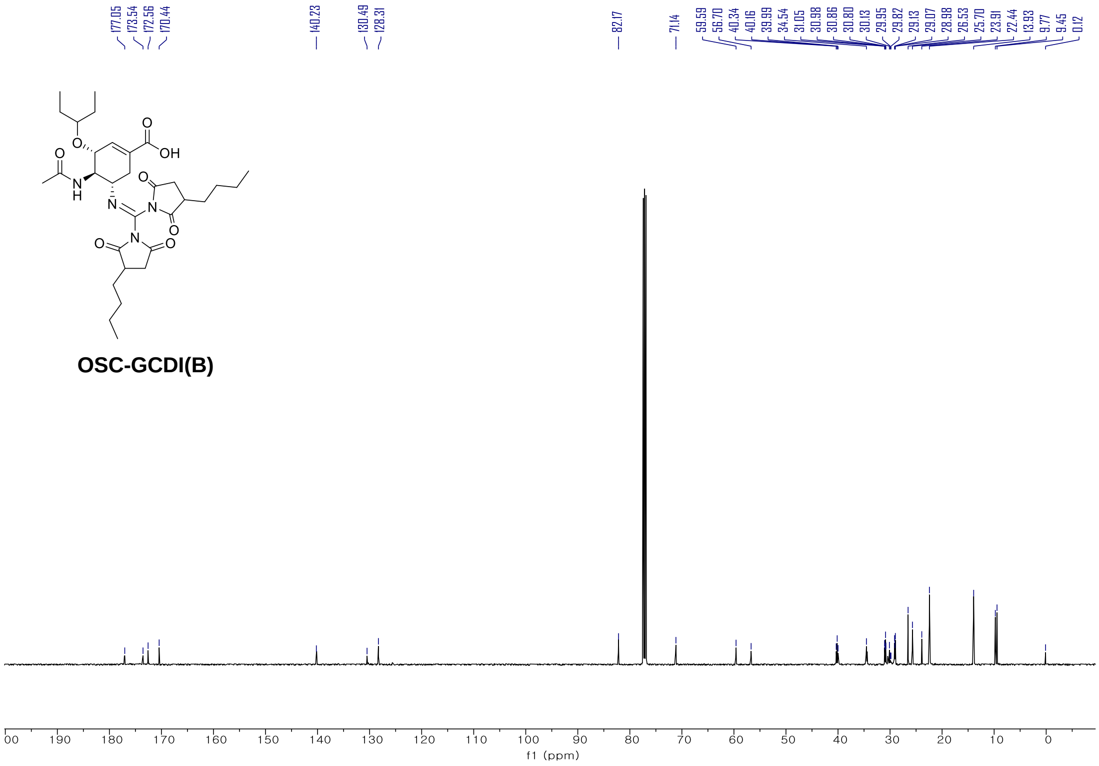

^{1}H NMR (500 MHz, $CDCl_3$)

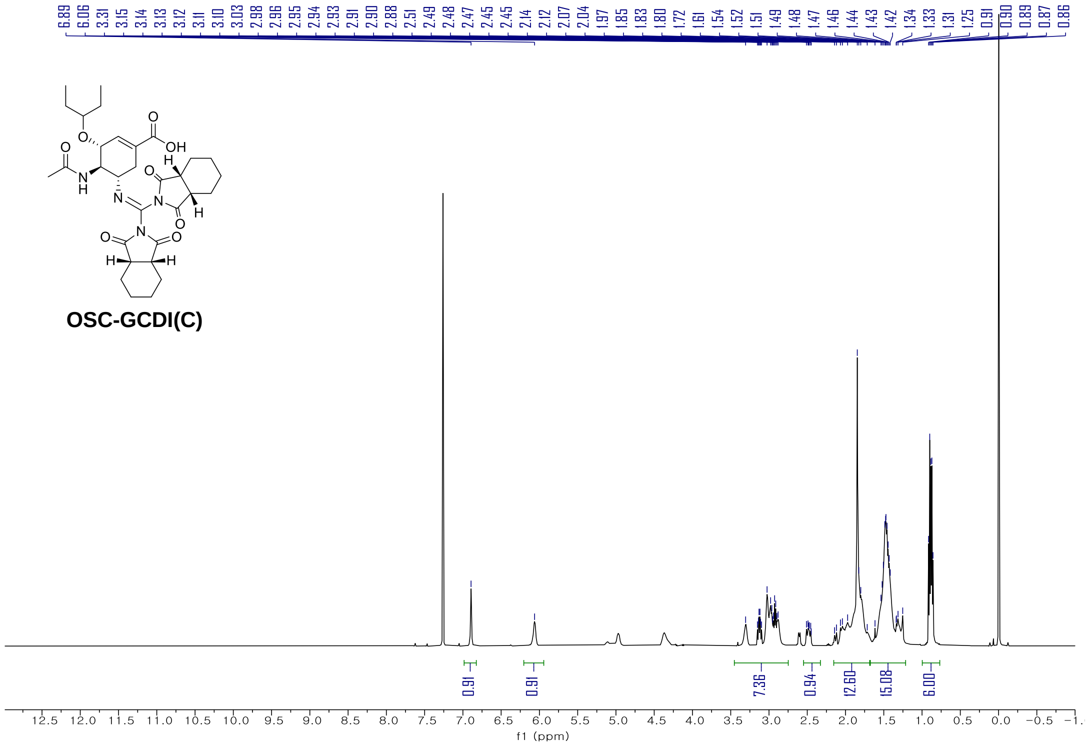

^{13}C NMR (125 MHz, $CDCl_3$)

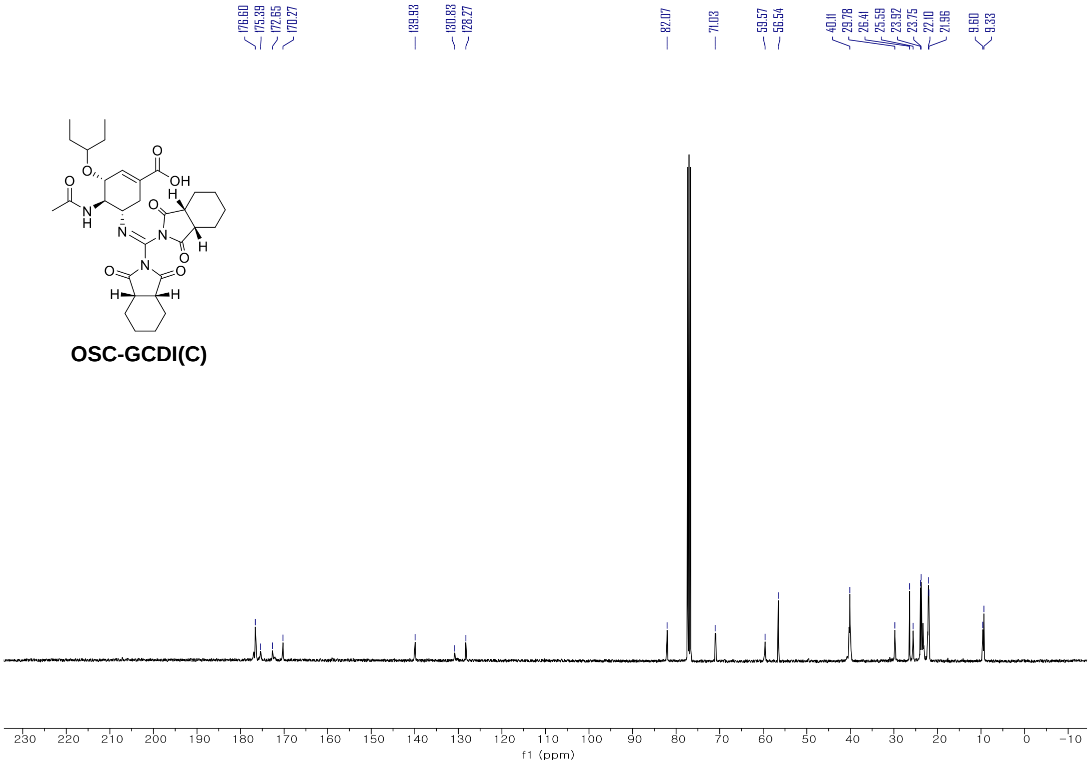

^{1}H NMR (500 MHz, $CDCl_3$)

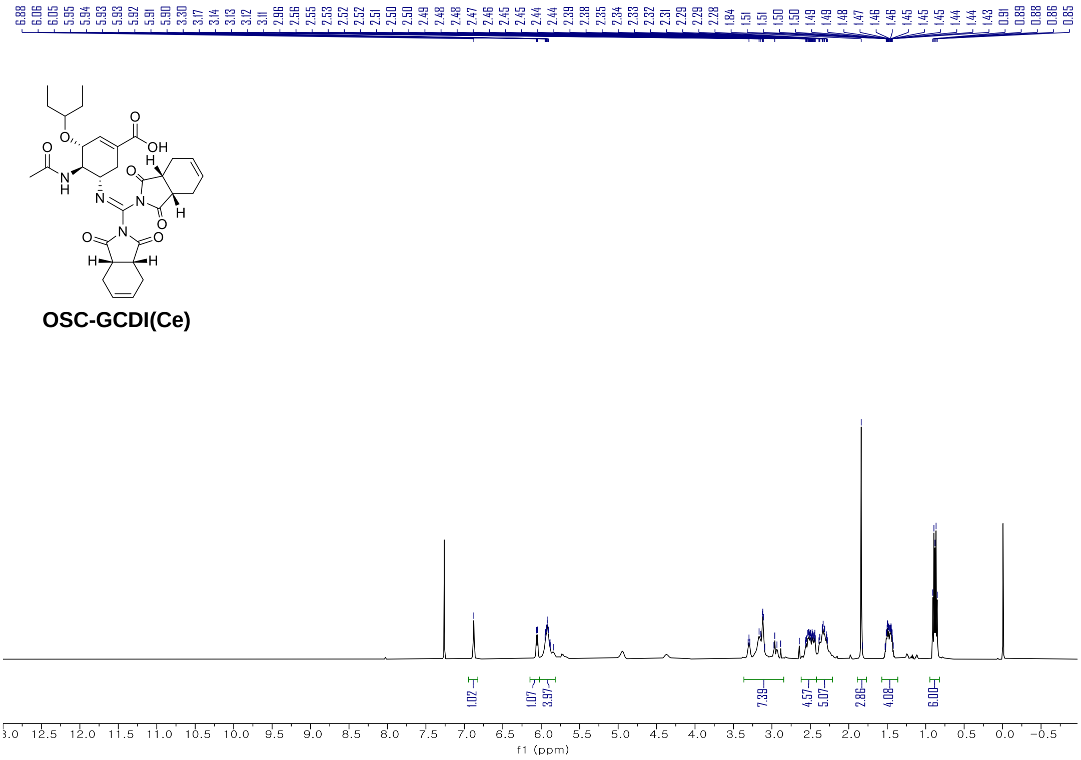

^{13}C NMR (125 MHz, $CDCl_3$)

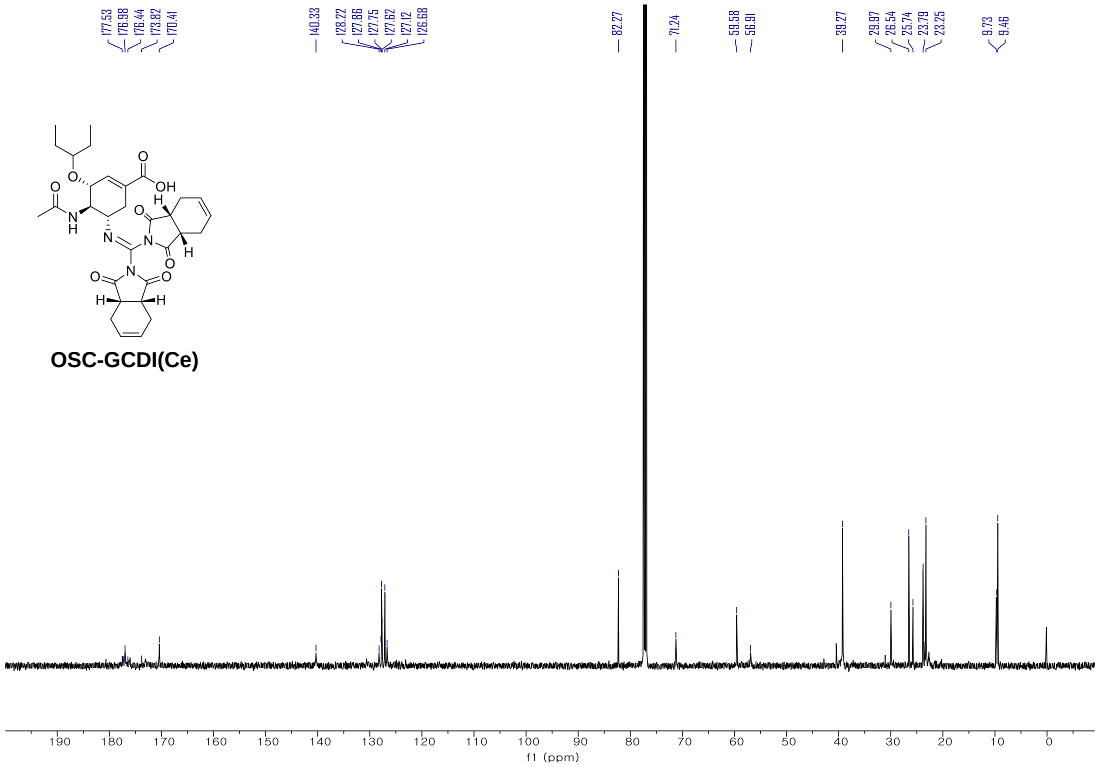

^1H NMR (500 MHz, $CDCl_3$)

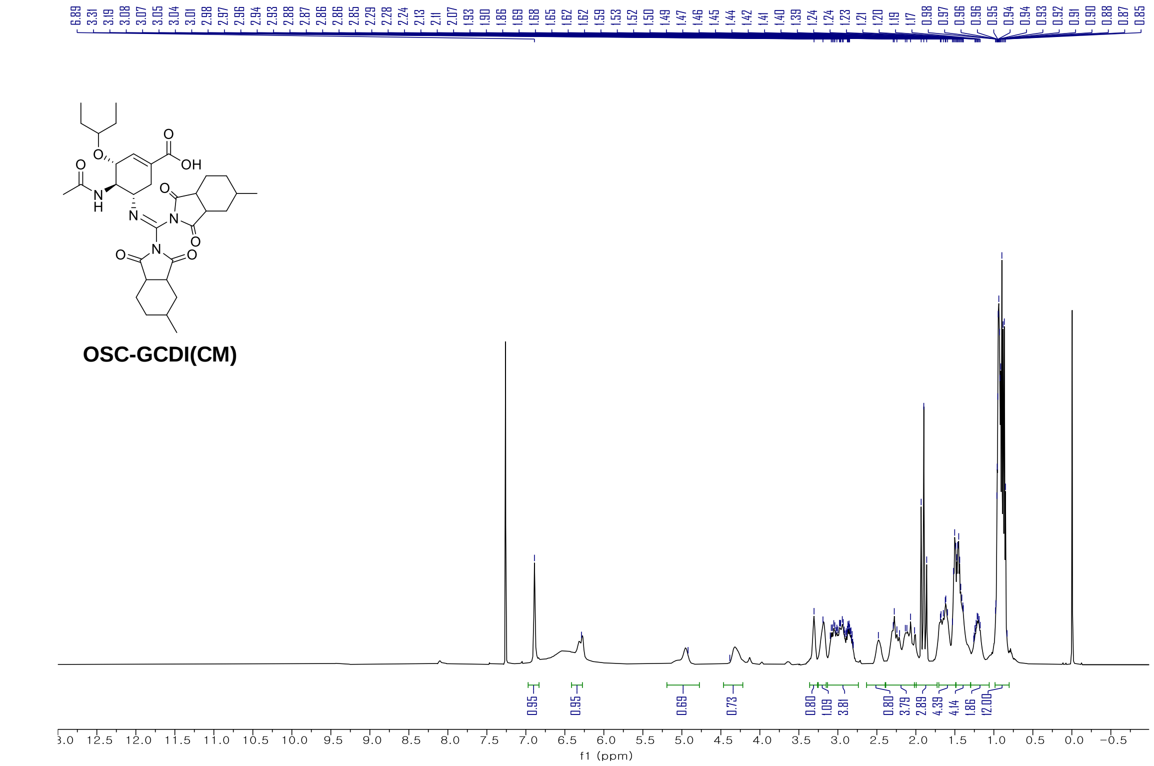

^{13}C NMR (125 MHz, $CDCl_3$)

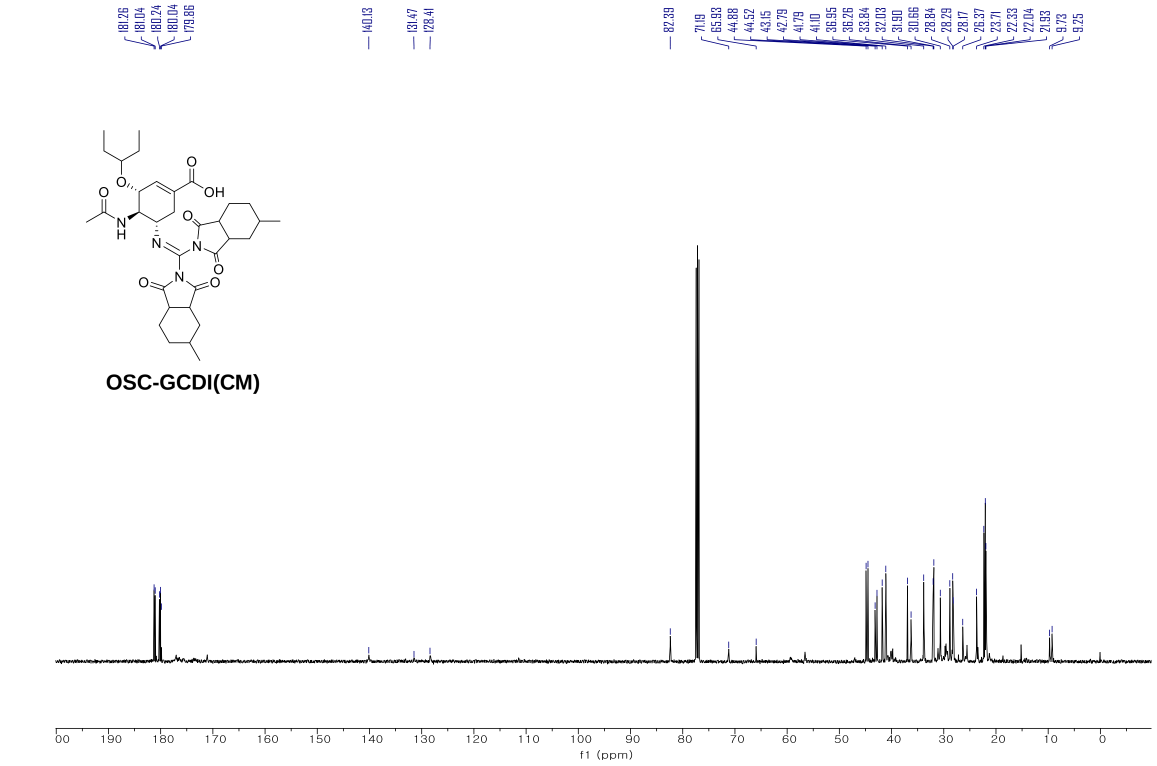

^{1}H NMR (500 MHz, $CDCl_3$)

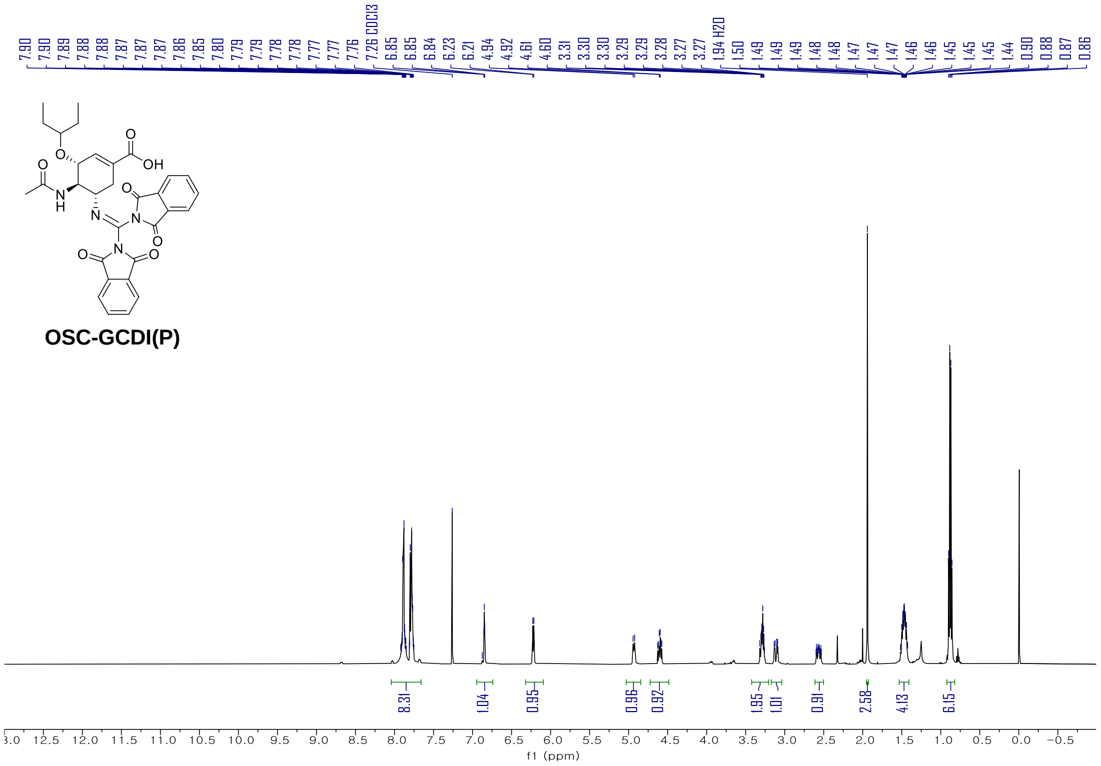

^{13}C NMR (125 MHz, $CDCl_3$)

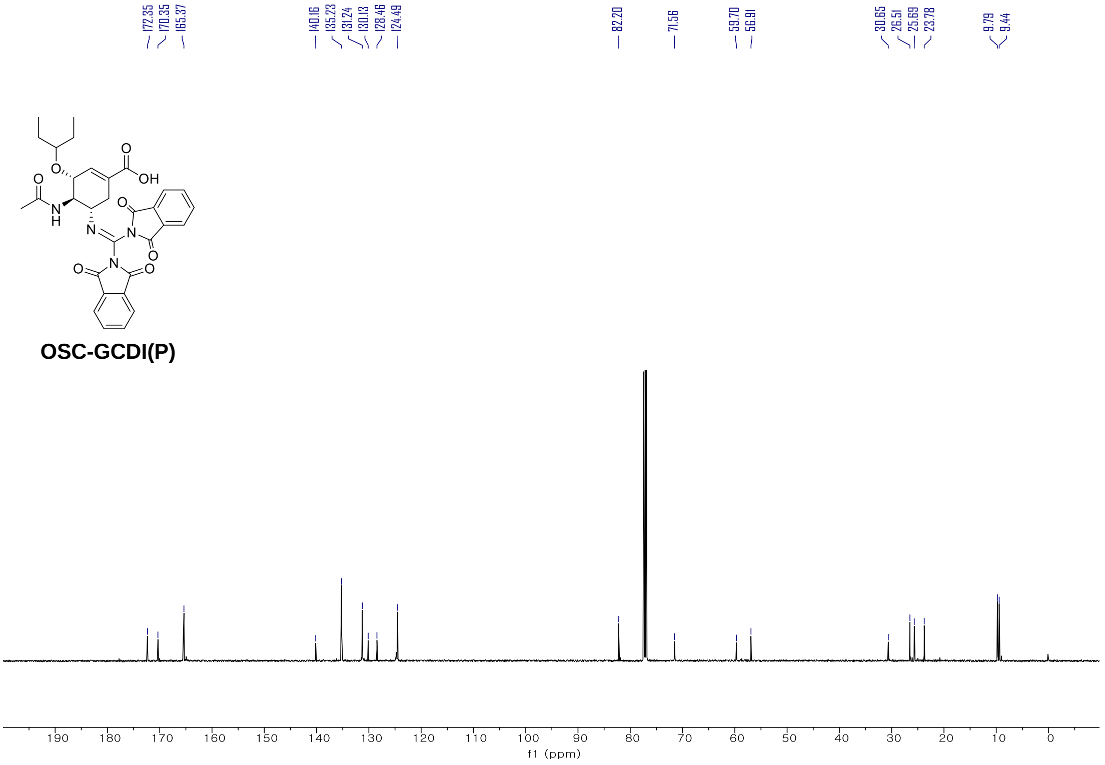

Supplemental References

(1) Jang, Y.; Shin, J. S.; Yoon, Y. S.; Go, Y. Y.; Lee, H. W.; Kwon, O. S.; Park, S.; Park, M. S.; Kim, M. Salinomycin inhibits influenza virus infection by disrupting endosomal acidification and viral matrix protein 2 function. *J. Virol.* **2018**, *92*, e01441-18.

(2) Feng, S.; Jiang, J.; Hu, P.; Zhang, J. Y.; Liu, T.; Zhao, Q.; Li, B. L. A phase I study on pharmacokinetics and pharmacodynamics of higenamine in healthy Chinese subjects. *Acta Pharmacol. Sin.* **2012**, *33*, 1353-1358.

(3) Shie, J.-J.; Fang, J.-M.; Wang, S.-Y.; Tsai, K.-C.; Cheng, Y.-S. E.; Yang, A.-S.; Hsiao, S.-C.; Su, C.-Y.; Wong, C.-H. Synthesis of tamiflu and its phosphonate congeners possessing potent anti-influenza activity. *J. Am. Chem. Soc.* **2007**, *129*, 11892-11893.

(4) Li, Z.; Meng, Y.; Xu, S.; Shen, W.; Meng, Z.; Wang, Z.; Ding, G.; Huang, W.; Xiao, W.; Xu, J. Discovery of acylguanidine oseltamivir carboxylate derivatives as potent neuraminidase inhibitors. *Bioorg. Med. Chem.* **2017**, *25*, 2772-2781.

(5) Albiñana, C. B.; Machara, A.; Řezáčová, P.; Pachl, P.; Konvalinka, J.; Kožíšek, M. Kinetic, thermodynamic and structural analysis of tamiphosphor binding to neuraminidase of H1N1 (2009) pandemic influenza. *Eur. J. Med. Chem.* **2016**, *121*, 100-109.
