# Supplementary material for: A Novel Prodrug Strategy Based on Reversibly Degradable Guanidine Imides for High Oral Bioavailability and Prolonged Pharmacokinetics of Broad-Spectrum Anti-influenza Agents
Source: ACS Cent Sci. 2024 Jul 4;10(8):1573–84. doi: 10.1021/acscentsci.4c00548 (PMC11363325; doi:10.1021/acscentsci.4c00548)
Supplement: Supplementary file 2 — oc4c00548_si_002.pdf [file oc4c00548_si_002.pdf]

oc-2024-005483.R1

Name: Peer Review Information for "A Novel Prodrug Strategy Based on Reversibly Degradable Guanidine Imides for High Oral Bioavailability and Prolonged Pharmacokinetics of Broad-Spectrum Anti-Influenza Agents"

First Round of Reviewer Comments

Reviewer: 1

Comments to the Author

In this study, a series of OSC prodrugs were synthesized from GOC and varying protective groups for guanidino group of GOC. The obtained results demonstrated that lipophilicity, effective permeability, and reversibility of OSC prodrugs could be tuned by altering the protective group. Ultimately, a formulation of OSC-GCDI(P) assisted with HPMCP achieved the enhanced antiviral activity compared with OS-P and GOC controls. However, compared with the initial ‘simple’ design hypothesis that a higher (or optimal) lipophilicity of prodrugs may improve their bioavailability for better therapeutic activity, the obtained results suggest a more complicated mechanism, which includes the protection of prodrugs by HPMCP and the covalent and/or non-covalent binding of prodrugs with serum proteins. Thus, the reviewer is unsure what type of protective groups is desired for the purpose. The CGDI(P) was readily degraded at neutral pH ($t_{1/2} < 1$ min). In this case, how was OSC-GCDI(P) bound to serum proteins to exhibit the long blood half-life? Can the prodrugs avoid metabolism associated with the first-pass effects in the liver? Overall, the reviewer requests major revision for better understanding.

1) The schematic illustration should be provided in the manuscript (or supporting information) to make readers readily understand the hypothesized transfer process of prodrugs in the body.

2) In Figure 2, OS-P should also be tested as a positive control that elicits a high bioavailability.

3) OSC-GCDI(P) showed a quite high degradability compared with the other prodrugs. The accelerated degradation mechanism should briefly be described in the manuscript.

4) The much lower bioavailability of OSC-GCDI(C) should be more clearly explained or discussed in the manuscript because the obtained result was contrary to the initial hypothesis.

5) The HPMCP coating may affect the bioavailability and therapeutic activity of the other control drugs, OS-P and GOC. Thus, the additional control experiments with OS-P/HPMCP and GOC/HPMCP should be further performed for better comparison with OSC-GCDI(P).

Reviewer: 2

Comments to the Author

In this study, scientists in South Korea developed a novel class of guanidine diimid-based guandinoseltamivir carboxylate prodrugs, compounds that exhibit excellent oral bioavailability and long pharmacokinetics against wild-type and OS-resistant influenza virus strains. The charge of the guanidine group is effectively temporarily masked by the GCDI group, increasing lipophilicity and promoting the absorption of the drug through the intestinal barrier. Based on a literature review, it can be conclusively considered that this is the first reported guanidine based prodrug that can covalently bind to serum proteins through biodegradable linkers. Guandinoseltamivir carboxylate is an active compound that can be continuously regenerated in the serum by hydrolyzing the linker. This study has pioneering significance. I think this study deserves to be published.

The study would be even more perfect if the authors could test the activity of the compound against more resistant strains.

Author's Response to Peer Review Comments:

Reviewers' comments and responses:

Reviewer #1

General comments:

In this study, a series of OSC prodrugs were synthesized from GOC and varying protective groups for guanidino group of GOC. The obtained results demonstrated that lipophilicity, effective permeability, and reversibility of OSC prodrugs could be tuned by altering the protective group. Ultimately, a formulation of OSC-GCDI(P) assisted with HPMCP achieved the enhanced antiviral activity compared with OS-P and GOC controls. However, compared with the initial 'simple' design hypothesis that a higher (or optimal) lipophilicity of prodrugs may improve their bioavailability for better therapeutic activity, the obtained results suggest a more complicated mechanism, which includes the protection of prodrugs by HPMCP and the covalent and/or non-covalent binding of prodrugs with serum proteins. Thus, the reviewer is unsure what type of protective groups is desired for the purpose. The CGDI(P) was readily degraded at neutral pH ($t1/2 < 1$ min). In this case, how was OSC-GCDI(P) bound to serum proteins to exhibit the long blood half-life? Can the prodrugs avoid metabolism associated with the first-pass effects in the liver? Overall, the reviewer requests major revision for better understanding.

>> **The stability of OSC-GCDI-protein conjugate**

We thank the reviewer for bringing out the very important point regarding the sustained release of **GOC** after conjugation of the **OSC-GCDI**s to the serum proteins. As noted by the reviewer, **OSC-GCDI(P)** shows rapid degradation kinetics in serum with a $t_{1/2}$ of 1.3 min (**Table 1**). However, the full activation of the prodrug, or the release of **GOC**, is much slower than the degradation kinetics in serum (**Fig. S2**). In the previous manuscript, we provided the evidence of the prodrug-protein conjugate (**Fig. 3** and **Fig. S4**), which supports significant stability of the conjugate.

To further support that **GOC** can be released from the prodrug-protein conjugates in a sustained manner, we additionally measured the **GOC** release kinetics from the **OSC-GCDI(P)**-HSA conjugate and **OSC-GCDI(C)**HSA conjugate. The result was included as **Fig. S5** in the revised manuscript. Consistent with our initial hypothesis, sustained release of **GOC** from the conjugates was observed for at least 72 h. The results support significant stability of the prodrug-protein conjugate for several days and slow release kinetics of **GOC** in the blood circulation. We also described the release kinetics in the main text as below.

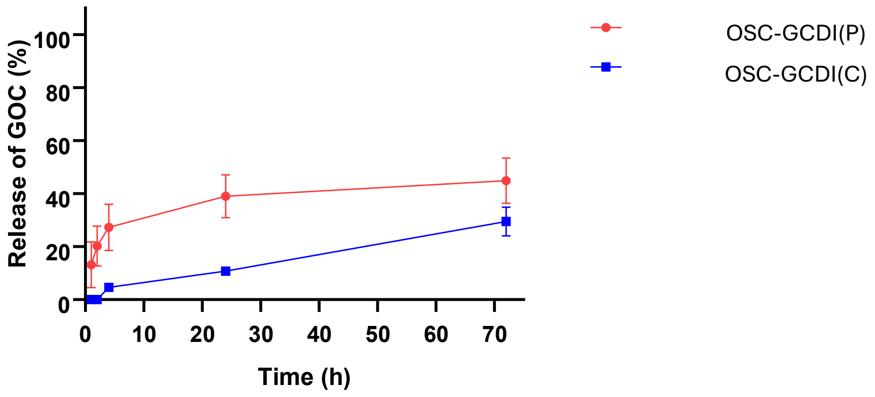

Fig. S5. The release profiles of **GOC** from the **OSC-GCDI**s conjugated to human serum albumin (HSA) at 37 ℃. Each data point represents the mean ± SD (*n* = 3).

>> Page 5, paragraph 3, line 10: Indeed, incubation of **OSC-GCDI(P)**-HSA and **OSC-GCDI(C)**-HSA conjugates resulted in the release of **GOC** in a sustained manner for at least 72 h (Fig. S5).

>> Page 8, paragraph 4, line 25: Therefore, the lower F_t of **OSC-GCDI(C)** may be due to slower activation of **OSC-GCDI(C)** into **GOC** in the bloodstream (Fig. S2 and S5), which may lead to incomplete activation within 48 h in the rat model.

>> The liver metabolism of OSC-GCDI

We also thank the reviewer for providing the constructive comments regarding the hepatic first-pass effects of the prodrugs. To estimate the potential metabolism of the prodrugs in the liver, we evaluated the half-lives of two representative prodrugs, **OSC-GCDI(P)** and **OSC-GCDI(D)**, as well as the active species, **GOC**, in the presence of Sprague Dawley rat liver microsomes (RLM).

Table S1. *In vitro* microsomal stability of **GOC**, **OSC-GCDI(D)** and **OSC-GCDI(P)**.

		GOC	OSC-GCDI(D)	OSC-GCDI(P)
$T_{1/2}$ (min)[a]	Rat liver microsome[b]	6930	385	17.7
	pH 7.4 phosphate buffer[c]	n.t.[d]	495	14.1
CL_{int} (μL/mg/mg)[e]		0.200	3.60	78.2

[a] Determined by HPLC analysis of the remaining **GOC** or **OSC-GCDI** prodrugs at various time points. The calculation of the half-lives was based on the assumption of pseudo-first order kinetics of the degradation. [b] Examined in Sprague Dawley rat microsomes at a prodrug concentration of 100 μM at 37 °C. [b] Examined in phosphate buffered saline (50 mM phosphate, 154 mM ionic strength, 37 °C). [d] Not tested. [e] Intrinsic clearance.

First, **GOC** showed negligible decomposition in the presence of RLM (98.8% remaining **GOC** after 90-min incubation), which can support the **GOC** structure can escape the hepatic first-pass effects. Next, the degradation of the **OSC-GCDI** prodrugs was barely affected by RLM. The half-lives of **OSC-GCDI(P)** were determined as 17.7 min and 14.1 min with and without RLM, respectively. Also, **OSC-GCDI(D)** showed 385 min and 495 min of the half-lives with and without RLM. Based on these results, we infer that the degradation of GCDI moieties in the prodrugs, or the release of the active species, **GOC**, is predominantly by non-hepatic mechanism, possibly by the attack of various nucleophiles in blood. Moreover, it is hypothesized that the conjugation of the GCDI prodrug onto serum proteins may reduce its permeability to hepatocytes. This reduction could potentially diminish hepatic intracellular metabolism, thereby prolonging the drug's presence period in systemic circulation. While these mechanisms suggest a reduced likelihood of the first-pass effect, further studies to fully elucidate the pharmacokinetic behavior of the protein-conjugated form of **OSC-GCDI**s.

We added these experimental data to Supporting Information (**Table S1**) and corresponding description to our revised manuscript.

>> Page 4, paragraph 4, line 10: The degradation rate of **OSC-GCDI**s was not accelerated by Sprague Dawley rat liver microsomes (Table S1). The results supported that the degradation of **OSC-GCDI**s or activation to **GOC** might be based primarily on reactions in serum, not by hepatic metabolism.

>> Page 5, paragraph 3, line 3: Similar to other albumin-conjugated drugs,[43-46] the GCDI prodrugs are likely to remain in the bloodstream in their conjugated form with albumin, thereby reducing their hepatic intracellular metabolism and excretion rates.

Specific comments:

1) The schematic illustration should be provided in the manuscript (or supporting information) to make readers readily understand the hypothesized transfer process of prodrugs in the body.

>> We thank the reviewer for the thoughtful suggestion. Following the comments, we edited the schematic illustration (**Fig. 1c**) to describe the mechanism to enhance the intestinal absorption

and the pharmacokinetic half-lives of guanidine prodrugs by the GCDI formation and provided it to the revised manuscript.

>> Page 2, **Fig. 1c**:

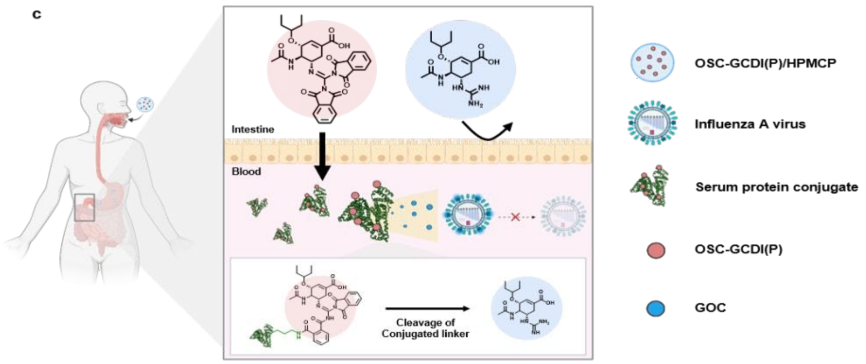

2) In Figure 2, OS-P should also be tested as a positive control that elicits a high bioavailability.

>> We agree with the reviewer's opinion. Following the comment, we evaluated log *P*, log $D_{7.4}$ and effective permeability in PAMPA assay of **OS-P**. **OS-P** exhibited much higher log *P* and log $D_{7.4}$ values than those of **GOC** (**Fig. 2a**, 0.22 vs -1.35 for log *P* and **Fig. 2b**, -0.42 vs -1.49 for log $D_{7.4}$). Also, **OS-P** showed moderate level of effective permeability through the artificial membrane (**Fig. 2c**, 2.27 × 10^{-6} cm/s) whereas **GOC** showed negligible permeability. On the other hand, the results show that the GCDI derivatization of **GOC** can lead to even higher lipophilicity and effective permeability of the **OSC-GCDI** prodrugs compared to **OS-P**. Given the efficient intestinal absorption of **OS-P** (oral bioavailability of 80%), these results are also consistent with high intestinal absorption of the **OSC-GCDI** prodrugs. We included the log *P*, log $D_{7.4}$ and effective permeability of **OS-P** in **Fig. 2** in our revised manuscript.

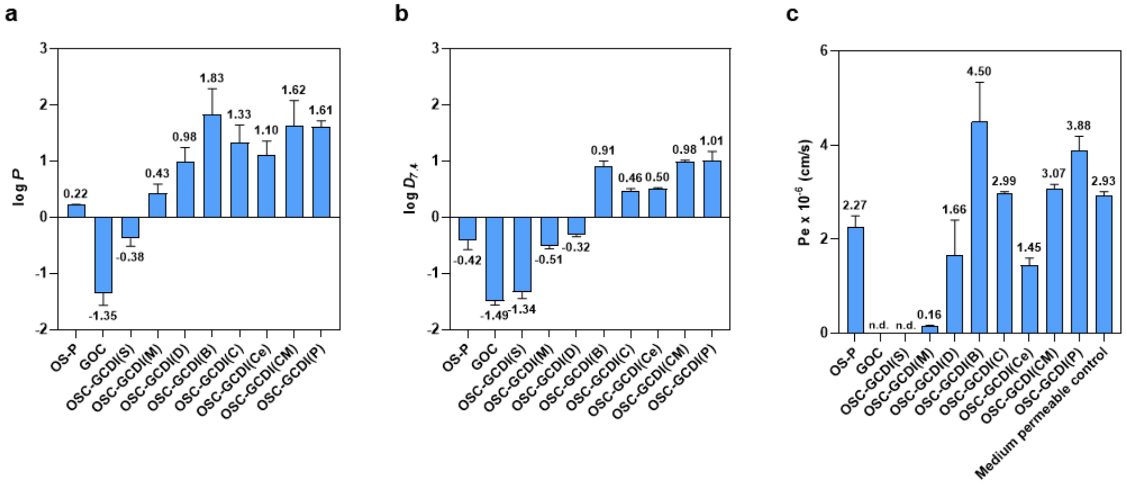

Figure 2. Lipophilicity and membrane permeability of OSC-GCDI prodrugs. (a) Octanol-water partition coefficients (log *P*) of **OS-P**, **GOC** and the **OSC-GCDI** prodrugs. (**b**) Octanol-phosphate buffer distribution coefficients at pH 7.4 (log $D_{7.4}$) of **OS-P**, **GOC** and the **OSC-GCDI** prodrugs. (**c**) Effective permeability through the artificial membrane of **OS-P**, **GOC** and the **OSC-**

GCDI prodrugs. The data are presented as means ± standard deviations (SD) (n = 3). n.d., not detected.

>> Page 3, paragraph 5, line 16: Notably, all the **OSC-GCDI**s except **OSC-GCDI(S)** and **OSC-GCDI(M)** exhibited even higher log P and log $D_{7.4}$ values than **OS-P**, which has excellent oral bioavailability.

>> Page 4, paragraph 2, line 7: It is noteworthy that four **OSC-GCDI**s, specifically **OSC-GCDI(B)**, **OSCGCDI(C)**, **OSC-GCDI(CM)**, and **OSC-GCDI(P)**, demonstrated P_e values that were comparable to or even exceeding those of **OS-P** (P_e = 2.27 × 10^{-6} cm/s) and a control compound with medium permeability (P_e = 2.93 × 10^{-6} cm/s).

3) **OSC-GCDI(P)** showed a quite high degradability compared with the other prodrugs. The accelerated degradation mechanism should briefly be described in the manuscript.

>> We thank the reviewer for the valuable comment. As the reviewer pointed out, **OSC-GCDI(P)** exhibits faster degradation kinetics compared to the other **OSC-GCDI** prodrugs. In case of amine-based imides, phthalimide and maleimide with sp^2-based α and β carbons show much faster hydrolysis rates compared to succinimide (100 and 300 times faster, respectively) (Buss, J. L.; Hasinoff, B. B. *J. Mol. Model.* **2001**, *7*, 438– 444). It has been suggested that this is due to additional ring strain of maleimide and phthalimide resulting from the rigidity of the imide rings required for maximal electron delocalization. In a similar manner, we carefully suspect that **OSC-GCDI(P)** with the phthalimide structure may be intrinsically less stable than other saturated **OSC-GCDI**s due to the higher ring strain of the imide rings.

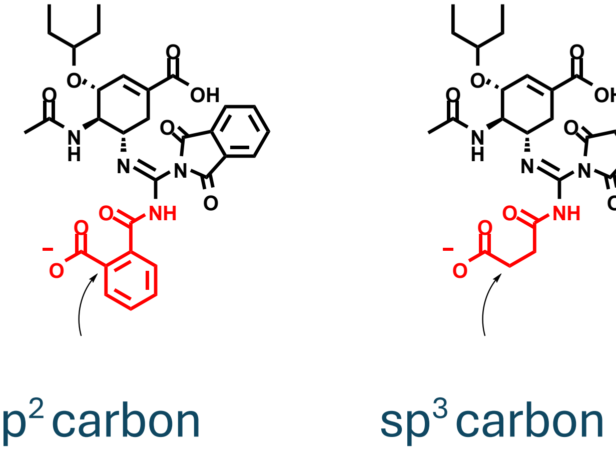

Additionally, we expect that the stability of the anionic amic acid intermediate, which can be generated by the hydrolysis of **OSC-GCDI**s, may further contribute to the different degradability of **OSC-GCDI**s. In general, aromatic carboxylic acids exhibit higher acidity than aliphatic acids since sp^2 carbon atoms show higher electronegativity than sp^3 carbon atoms, thereby stabilizing the negative charge of the carboxylate groups more efficiently. Indeed, benzoic acid (pK_a = 4.2) and acrylic acid (pK_a = 4.3) exhibit lower pK_a values compared to acetic acid (pK_a = 4.8) and cyclohexanecarboxylic acid (pK_a = 4.8). The better stabilization of the negative charge of the amic acid intermediate in the aromatic ring can contribute the faster degradation of **OSC-GCDI(P)** than other **OSC-GCDI**s with the aliphatic imide structures.

We included the discussion about the rapid degradability of **OSC-GCDI(P)** in the revised manuscript as below.

>> Page 4, paragraph 3, line 20: As suggested in previous studies,[42] we suspect that the exceptional hydrolysis rate of **OSC-GCDI(P)** can be attributed to additional ring strain of the phthalimide structure to maximize the electron delocalization, which may lead to intrinsic instability of **OSC-GCDI(P)**. Additionally, given that aromatic carboxylic acids generally have higher acidity than aliphatic acids, the negative charge of the amic acid intermediate, which is generated by the degradation of **OSC-GCDI(P)**, is expected to be stabilized more efficiently in the aromatic ring compared to those from other **OSC-GCDI**s with the aliphatic imide structures. These characteristics may contribute to the faster hydrolysis of **OSC-GCDI(P)**.

4) The much lower bioavailability of **OSC-GCDI(C)** should be more clearly explained or discussed in the manuscript because the obtained result was contrary to the initial hypothesis.

>> It is actually an important point. As shown in **Fig. 4**, the oral bioavailability (F_t) of **OSC-GCDI(C)**/HPMCP was calculated as 28% in rat, which is significantly lower than that of **OSC-GCDI(P)**/HPMCP although the passive diffusion rate of **OSC-GCDI(C)** through the intestinal barrier estimated in PAMPA assay is comparable to that of **OSC-GCDI(P)** (**Fig. 2c**, 2.99×10^{-6} cm/s vs 3.88×10^{-6} cm/s). From these results, we initially expected that **OSC-GCDI(C)** and **OSC-GCDI(P)** would have similar intestinal absorption rate into the bloodstream. However, given that oral bioavailability of a prodrug is defined as the molar ratio of the detected active species (**GOC** in this case) to the orally administered prodrug, not only intestinal absorption of the **OSC-GCDI**s, but also activation into **GOC** is crucial for high bioavailability.

In this regard, we carefully hypothesize that the oral bioavailability of **OSC-GCDI(C)** was calculated as lower values than the actual absorbed values in the PK analyses because **OSC-GCDI(C)** is activated to **GOC** much slower than **OSC-GCDI(P)** as shown in the activation profiles (**Fig. S2**). Consistent with the hypothesis, the F_t values of both the prodrugs were calculated higher in the 48 h-measurement in rats compared to in the 24h-measurment in mice. Indeed, the **GOC** release profile from **OSC-GCDI(C)**/HPMCP in rat models exhibits almost constant plateau even at the time point of 48 h (**Fig. 4c**). Taken together, we expect that the oral bioavailability of **OSC-GCDI(C)** may be calculated more accurately when the pharmacokinetic analysis is performed in a longer period of time (>48 h) possibly by using larger animal models.

In addition, faster release of **GOC** was observed from **OSC-GCDI(P)**-HSA conjugate compared to **OSCGCDI(C)**-HSA conjugate, which also agrees with the activation profiles of the **OSC-GCDI**s in serum (**Fig. S2**) and longer *in vivo* half-life of **OSC-GCDI(C)** than that of **OSC-GCDI(P)** in mice and rat models (**Fig. 4**).

We included short discussion about this matter in the revised manuscript as below.

>> Page 8, paragraph 4, line 17: In addition, given that the P_e value of **OSC-GCDI(C)** is comparable to that of **OSC-GCDI(P)** (**Fig. 2c**), we suspect that the lower F_t value of **OSC-GCDI(C)** compared to that of **OSCGCDI(P)** probably did not result from poor intestinal absorption of **OSC-GCDI(C)**.

Considering that F_t is defined as the molar ratio of detected active species (**GOC**) to orally administered prodrug (**OSC-GCDI**), not only intestinal absorption, but activation rate *in vivo* is also a crucial factor. Therefore, the lower F_t of **OSCGCDI(C)** may be due to slower activation of **OSC-GCDI(C)** into **GOC** in the bloodstream (**Fig. S2** and **S5**), which may lead to incomplete activation within 48 h in the rat model. Moreover, **OSC-GCDI(C)**/HPMCP and **OSC-GCDI(P)**/HPMCP exhibited $T_{1/2}$ of 40.97 h and 10.29 h, respectively, which were significantly longer than that of **GOC·TFA** (3.57 h). The four times longer $T_{1/2}$ of **OSC-GCDI(C)** than that of **OSC-GCDI(P)** also supports our argument for the slower activation of **OSC-GCDI(C)** than **OSC-GCDI(P)**.

5) The HPMCP coating may affect the bioavailability and therapeutic activity of the other control drugs, **OSP** and **GOC**. Thus, the additional control experiments with **OS-P**/HPMCP and **GOC**/HPMCP should be further performed for better comparison with **OSC-GCDI(P)**.

>> We agree with the reviewer's opinion for the need of the control experiments with **OS-P**/HPMCP and **GOC**/HPMCP to rule out the possibility that HPMCP itself may influence the bioavailability and activity of the (pro)drugs. Unfortunately, however, encapsulation of hydrophilic **GOC** into HPMCP was highly inefficient and the drug contents of the resulting **GOC**/HPMCP was calculated as 32 μg/mg, significantly lower than those of the **OSC-GCDI(C)**/HPMCP (327 μg/mg) and **OSC-GCDI(P)**/HPMCP (503 μg/mg) (**Fig. S9**). Even for **OSP**, encapsulation into HPMCP was extremely difficult. Due to the poor drug contents, direct evaluation of the oral bioavailability of **OS-P**/HPMCP and **GOC**/HPMCP in mice or rat models is technically difficult to be achieved.

Instead, we carried out the PAMPA assay of **GOC**/HPMCP and **OSC-GCDI(P)**/HPMCP to compare the effective permeability (P_e) of the (pro)drugs encapsulated in HPCMP with bare (pro)drugs (**Table S2**). As results, **GOC**/HPMCP showed negligible permeability through the artificial membrane. Additionally, P_e of **OSC-GCDI(P)**/HPMCP was measured as 2.63×10^{-6} cm/s, which is not much different from that of bare **OSC-GCDI(P)** (3.07×10^{-6} cm/s).

Table S2. Effective permeability through the artificial membrane of **GOC** and **OSC-GCDI(P)** with and without HPMCP encapsulation.

	**GOC**	**GOC**/HPMCP	**OSC-GCDI(P)**	**OSC-GCDI(P)**/HPMCP
$P_e \times 10^{-6}$ (cm/s)	n.d.	n.d.	3.88±0.31	2.63±0.20

The data are presented as means ± standard deviations (SD) (*n* = 3). n.d., not detected.

Taken together, we believe that these results support that HPMCP encapsulation could prevent premature degradation of the GCDI structure in the gastrointestinal tract but HPMCP itself could not improve the intestinal absorption and bioavailability of the (pro)drugs. We added these data to the Supporting Information (**Table S2**) and discussion to the revised manuscript as below.

>> Page 8, paragraph 3, line 6: The HPMCP encapsulation itself has no significant effect on the passive diffusion into membranes as shown in the PAMPA results of **GOC**/HPMCP and **OSC-GCDI(P)**/HPMCP (Table S2).

Reviewer #2

General comments:

In this study, scientists in South Korea developed a novel class of guanidine diimid-based guandinoseltamivir carboxylate prodrugs, compounds that exhibit excellent oral bioavailability and long pharmacokinetics against wild-type and OS-resistant influenza virus strains. The charge of the guanidine group is effectively temporarily masked by the GCDI group, increasing lipophilicity and promoting the absorption of the drug through the intestinal barrier. Based on a literature review, it can be conclusively considered that this is the first reported guanidine based prodrug that can covalently bind to serum proteins through biodegradable linkers. Guandinoseltamivir carboxylate is an active compound that can be continuously regenerated in the serum by hydrolyzing the linker. This study has pioneering significance. I think this study deserves to be published. The study would be even more perfect if the authors could test the activity of the compound against more resistant strains.

>> We thank the reviewer for the positive evaluation regarding the quality of the work and appreciate the constructive comments. In this study, we mainly focused on development of the GCDI prodrugs of **GOC** which targets H275Y mutation-bearing OS-resistant influenza virus and showed *in vivo* antiviral activity of **OSC-GCDI(P)** against one resistant strain (rgA/Korea/09/2009Δ53-60). Because our prodrugs also showed the *in vitro* activity against various influenza A and B strains with and without the H275Y mutation (**Table 2**), we believe that they also showed the effective therapeutic effect *in vivo*. We would test the activity of the compounds against more resistant strains *in vivo* in near future. Furthermore, since our strategy can be generally applied for the drugs or drug candidates containing guanidine groups in principle, we are continuing the research to expand the scope of our GCDI prodrug strategy to other guanidine drugs for treatment of various diseases.

oc-2024-005483.R2

Name: Peer Review Information for "A Novel Prodrug Strategy Based on Reversibly Degradable Guanidine Imides for High Oral Bioavailability and Prolonged Pharmacokinetics of Broad-Spectrum Anti-Influenza Agents"

Second Round of Reviewer Comments

Reviewer: 1

Comments to the Author

The manuscript has been revised well for publication.

Reviewer: 2

Comments to the Author

I am very satisfied with the author's reply
